# Proteomic and Ubiquitinated Proteome Insights Into ER Stress Responses in Chinese Hamster Ovary Cells Under Mild Hypothermic Conditions

**DOI:** 10.1002/bit.70081

**Published:** 2025-10-28

**Authors:** David Ryan, Christiana‐Kondylo Sideri, Michael Henry, Selvaprakash Karuppuchamy, Esen Efeoglu, Paula Meleady

**Affiliations:** ^1^ Life Sciences Institute Dublin City University Dublin Ireland; ^2^ School of Biotechnology Dublin City University Dublin Ireland; ^3^ School of Food Science and Environmental Health Technological University Dublin Dublin Ireland; ^4^ SSPC Research Ireland Centre for Pharmaceuticals Limerick Ireland

**Keywords:** Chinese hamster ovary (CHO) cells, endoplasmic reticulum (ER) stress, endoplasmic reticulum associated degradation (ERAD), hypothermia, protein folding, ubiquitination, unfolded protein response (UPR)

## Abstract

Chinese hamster ovary (CHO) cells, widely utilised in biopharmaceutical production, experience various stressors during cell culture that can affect protein expression and folding, particularly within the endoplasmic reticulum (ER). Mild hypothermia is widely employed in CHO cell bioproduction to improve recombinant protein yield and quality; however, its impact on ER‐associated pathways, particularly those governing protein folding and stress responses, remains insufficiently characterised. Mass spectrometry‐based proteomics allows for the identification and relative quantification of proteins, enabling detailed insights into protein expression, modifications, and functional networks. This study investigates the impact of mild hypothermic conditions (31°C) on the whole cell proteome and ubiquitinated proteome of CHO cells, with a specific focus on ER proteins and ER stress. Using high‐resolution mass spectrometry, we conducted a comprehensive proteomic and ubiquitinated proteomic analysis to quantify changes in protein abundance and ubiquitinated peptides under mild hypothermia. The downregulation of several proteins involved in the glycosylation of nascent polypeptides at 31°C, including DDOST, P4HB, PRKSCH and LMAN1, in all cell lines studied suggests that mild hypothermic shock disrupts the cell's normal ability to fold new proteins, leading to ER stress as the misfolded proteins build up. When this is coupled with the maintained cell viability and increased productivity at 31°C, it indicates the ER stress response can mitigate the build‐up of misfolded proteins. The differential regulation of the transcription factor eIF2α, downregulated in non‐producer cells but upregulated in producer cells at 31°C, suggests that recombinant protein‐producing CHO cells possess a more adaptive ER stress response, enabling more efficient function under hypothermic culture conditions. Enhanced ubiquitination of misfolded protein substrates highlights an increased reliance on ER‐associated degradation (ERAD) pathways to alleviate proteotoxic stress, as well as the wide range of biological processes that are regulated by ubiquitination as part of the hypothermic stress response. These findings provide new insights into the cellular adaptation mechanisms of CHO cells to mild hypothermia, with implications for optimising bioproduction strategies to improve yield and quality of therapeutic proteins. Our study highlights the importance of understanding the more complex aspects of the proteome and how this additional layer of detail can open new avenues for CHO cell engineering.

## Graphical abstract

Proteomic analysis of CHO cells under mild hypothermia (31°C) reveals ER stress responses, ubiquitination dynamics, and adaptive mechanisms. Magnetic immuno‐affinity enrichment and mass spectrometry highlight protein regulation differences in non‐producer versus producer cells, offering insights for optimising biopharmaceutical production of IgG1 and human erythropoietin with enhanced therapeutic protein quality and yield.

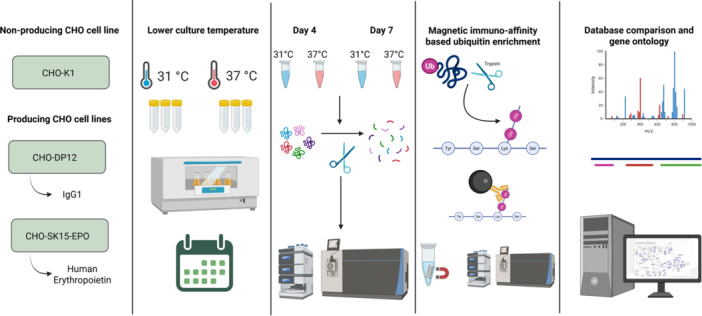

## 1 Introduction

The role of Chinese hamster ovary (CHO) cells in the biopharmaceutical industry is vital. Since the first CHO derived biotherapeautic, *Activase*, was given FDA approval in 1987, CHO cells have become the workhorse of the industry. Since then, CHO cells have become the preferred cell line for recombinant protein production, with 70% of recombinant protein therapies produced in CHO cells (Li et al. 2021). The popularity of CHO cells over other cell types is due to several key advantages, including: (i) reliable growth in chemically‐defined and serum‐free media, (ii) easily adapted to large‐scale suspension culture, (iii) the efficiency and accessibility of established gene editing and amplification techniques that allow for a specific productivity greater than other mammalian host cells, (iv) a well‐documented history as a safe host cell line, with approval to market achieved by many CHO derived therapeutics, and (v) the ability to replicate human‐like posttranslational modifications on the recombinant proteins they produce (J. Y. Kim et al. 2012). Research on several CHO cell lines has shown that lowering culture temperature to between 31°C and 33°C can have favourable effects on productivity. Lower culture temperature decreases cell growth rate, but this shift can result in an overall greater titre of protein product in the majority of CHO cell lines. It has been found that culture temperature of 35°C and the addition of 80 µM Zn^2+^ can increase monoclonal antibody (mAb) titre, and increase charge variants of the antibody, which is a crucial factor for product quality (Weng et al. 2020). CHO cells producing C‐terminal α‐amidating enzyme (Furukawa and Ohsuye 1999), secreted alkaline phosphatase (Kaufmann et al. 1999) and EPO (Yoon et al. 2003) all demonstrated an increased recombinant protein yield when cultured at temperatures lower than 37°C. Yoon et al. suggested that differences in culture response to lower temperatures could be due to differences in the integration sites of the antibody gene, which occur during gene amplification caused by the sometimes random nature of chromosomal rearrangement (Yoon et al. 2004). It has been shown that a lower culture temperature (32°C) allowed for greater protein processing and folding capacity in rCHO cells by upregulating genes involved in the IRE1/XBP1 branch of the Unfolded Protein Response (UPR), while a simultaneous downregulation of proteins related to ERAD activity occurred (Torres et al. 2021). This study identified several physiological changes in rCHO cells cultured at reduced temperature, including the upregulation of genes encoding key UPR‐specific transcriptional activators (e.g. *XBP1*s, *DDIT3*, *ATF5*) and ER‐resident proteins (e.g. *GRP78, GRP94, TRIB3*), which are critical for maintaining protein folding homeostasis in the ER (Torres et al. 2021). In another study, a 25% increase in specific productivity of a mAb was observed when the temperature was lowered to 32°C (Sou et al. 2015). However, a decreased proportion of more processed glycan structures, and a significant decrease in the expression of N‐glycosyltransferases responsible for N‐glycan branching and elongation of nascent peptides was observed in the lower temperature culture (Sou et al. 2015).

Protein folding within the ER is particularly important for glycoproteins, with 90% of glycoproteins undergoing the covalent addition of N‐linked glycans in the ER (Helenius et al. 1997). The co‐translational glycosylation of these polypeptides happens via the transfer of a preassembled oligosaccharide, Glc_3_Man_9_GlcNAc_2_, to the nascent polypeptides (Weerapana and Imperiali 2006). Glucosidases present in the ER continue to process the peptides to generate the monoglucosylated species (Glc_1_Man_9_Glc‐NAc_2_) that can be recognised by the membrane‐bound lectin‐chaperone calnexin (CNX) and its soluble orthologue calreticulin (CRT), both of which are needed to orchestrate the correct folding of the glycosylated polypeptides into glycoproteins (Rutkevich and Williams 2011). If the newly formed proteins have been correctly folded, then the chaperones are released from the protein, and are recirculated in the ER (Ito et al. 2015). If, however, the proteins have been incorrectly folded, the glucosyltransferase UGGT1 re‐glucosylates these proteins to create a peptide that will be recognised by CNX and CRT (Ito et al. 2015).

The disruption of normal ER function, termed ER stress, can be caused by internal or external factors such as high protein demand, viral infections, environmental toxins, inflammatory cytokines, and mutant protein expression (Oslowski and Urano 2011). In response to the build‐up of unfolded proteins, the cell activates the UPR (Bettigole and Glimcher 2015). The complex machinery of the ER can become exhausted by overexpression of recombinant human wild‐type proteins in CHO cells, proteins identical to those naturally produced in the human body, as shown in studies investigating blood coagulation factor VIII, a blood clotting factor that can be used in the treatment of haemophilia (Dorner et al. 1989) and antithrombin III, an anticoagulant that can be administered to treat deep vein thrombosis or pulmonary embolism (Schröder and Friedl 1997). This suggests that the extra workload CHO cells need to produce recombinant proteins may lead to an increase in misfolded proteins, inducing the conditions of ER stress. Three ER transmembrane sensors act as the main control of the UPR, inositol‐requiring enzyme 1 (IRE1), activating transcription factor 6 (ATF6) and protein kinase R‐like ER kinase (PERK) (Qu et al. 2021). When ER function is normal, these proteins are bound to the ER chaperone protein, BIP (GRP78), and are kept inactive; however, as misfolded proteins accumulate, BIP will dissociate from these proteins to bind to unfolded proteins, thus causing activation of the UPR (Gardner and Walter 2011). A detailed understanding of ER function enables targeted genetic engineering of CHO cells to improve recombinant protein productivity. For example, enhanced expression of BiP, ATF6c, and XBP1s has been shown to increase IgG specific productivity (Qp) in the transiently transfected CHO‐K1‐derived cell line EB27 (Pybus et al. 2014). Similarly, overexpression of ATF4 in CHO 13D‐35D cells significantly boosted the production of recombinant human antithrombin III (Ohya et al. 2008). Additionally, knocking out the PERK branch of the UPR, along with the apoptosis‐related factors Bax and Bak, in a DG44 mAb1‐producing CHO cell line resulted in a 2–3‐fold increase in specific productivity (Castellano et al. 2023).

The ERAD pathway is responsible for targeting misfolded proteins for degradation once they are recognised by the ER quality control machinery. Although the precise mechanisms of retro‐translocation remain incompletely understood, current evidence suggests that this process is primarily mediated by the HRD1 complex, composed of HRD1, HRD3, DER1, USA1 and YOS9, alongside several other essential proteins and cofactors (Wu et al. 2020). ER Degradation Enhancing α‐Mannosidase‐Like proteins (EDEMs) recognise misfolded glycoproteins by binding to exposed mannose residues, thus targeting these glycoproteins for retro‐translocation and subsequent degradation (Wang and Hebert 2003). The energy needed for retro‐translocation is provided by ATPases, such as p97/VCP (Ahlstedt et al. 2022). Ubiquitin‐regulatory X (UBX) proteins assist in directing substrates from the ERAD pathway towards autophagy (Kilgas and Ramadan 2023), an alternative degradation route involving lysosomes.

In eukaryotic cells, the ubiquitin family of proteins vary in their peptide sequence, but as a result of folding, all show similar final structures, with most ubiquitin proteins terminating with a di‐glycine sequence characteristic of this protein family, which is exposed following proteolytic processing (Pickart and Eddins 2004). Ubiquitin is found free in the cell but is more commonly seen as part of a protein‐ubiquitin conjugate (Bloom et al. 2003). This conjugation is formed via an isopeptide bond between the terminal glycine of ubiquitin and an amino group of the target protein, most commonly a lysine residue, but it is also possible for N‐terminal ubiquitination to occur (Bloom et al. 2003).

Ubiquitination plays a critical role in the cellular response to ER stress. Ubiquitination influences UPR signalling and outcomes by regulating protein degradation and trafficking (Lopata et al. 2020). Ubiquitination targets misfolded proteins, marking them for proteasomal degradation or autophagic clearance. Additionally, ubiquitination modulates the stability and activity of key UPR and ERAD components, such as transcription factors and chaperones, fine‐tuning the cellular response to ER stress (Carvalho et al. 2010; Denic et al. 2006). Although the ubiquitinated proteome of CHO cells is not yet well explored, this posttranslational modification appears to be an important factor in protein signalling and ER stress pathways in CHO cells (Jung et al. 2015; Selvaprakash et al. 2024). Several studies have highlighted the relevance of ubiquitin‐mediated mechanisms in the productivity of CHO cells. Xu et al. found that the addition of the ubiquitin‐ligase inhibitor thalidomide prevented the intracellular degradation of the recombinant protein HSA‐HGF at the later stages of CHO fed‐batch culture, allowing for more efficient and stable secretion of the recombinant product (Xu et al. 2017). Tang et al. characterised a novel proteolysis/proteasome‐dependent pathway involved in the degradation of antibody heavy chains by the ligases UBR4 and UBR5, and found that reducing UBR4/UBR5 expression before the production phase, mAb productivity in CHO cells was increased (D. Tang et al. 2020). The inhibition of proteasomal activity in two producer CHO cell lines, expressing a model mAb and a fusion protein, during CHO host and recombinant cell pool selection, yields pools with improved product titres and specific productivity, including for a difficult‐to‐express molecule. This suggests that reducing ubiquitin‐mediated clearance of recombinant proteins during selection favours cells with greater folding and secretion capacity (Knight et al. 2021). These findings indicate that ubiquitination is intrinsically linked with recombinant protein production in CHO cells. Investigating the ubiquitinated proteome further could identify additional degradation targets and pathways, possibly optimising production strategies across diverse biotherapeutic proteins. The importance of characterising other cellular posttranslational modifications (PTMs) in proteomic studies has also been highlighted in recent publications. Characterisation of the global phosphoproteome of high/low producing industrially relevant CHO cell lines revealed phosphorylation of the kinase GCN2 is increased in high Qp cells, suggesting that phosphorylation plays an important role the function of this protein (Bryan et al. 2021). Histone modifications in CHO‐K1 cells over a 9‐day batch culture show correlations with gene expression levels, demonstrating regulation beyond simple presence/absence switches (Hernandez et al. 2019). Through metabolomic engineering, the build‐up of toxic by‐products can be reduced and protein production increased in CHO cells (Richelle and Lewis 2017), and a detailed understanding of active PTMs in CHO cells could expand these results by increasing our knowledge of protein behaviour.

In‐depth knowledge of CHO cell stress responses enables the development of more resilient cell lines. For example, Jiang et al. (2016) generated a stably transfected CHO cell line (CHO‐GRP78) with a 1.5–2.5‐fold increase in BiP compared to the parental cell line. It was found that these modified cells had increased viability and reduced apoptosis when exposed to serum deprivation and oxidative stress (Jiang et al. 2016). It has also been shown that overexpression of BiP in CHO cells provides protection from ribose‐induced cytotoxicity, while BiP knockdown led to a marked decrease in cell viability (Wu et al. 2018).

In this study, the role of ER stress mechanisms triggered by exposure to mild hypothermic conditions on the whole cell proteome and ubiquitinated proteome was investigated in three CHO cell lines, CHO‐K1, CHO‐DP12 which produces an anti‐IL8‐IgG, and CHO‐SK15‐EPO which produces human erythropoietin (EPO) using quantitative label‐free mass spectrometry‐based proteomics and subsequent Gene Ontology (GO) analysis. By lowering the culture temperature to the lowest temperature commonly used in industrial setting (31°C), we aimed to trigger a pronounced ER stress response. Cells were subjected to a temperature downshift from 37°C to 31°C forty‐eight h after seeding. Differential expression (DE) analysis of the whole cell proteome was carried out at two time points during culture, day four and day seven, followed by GO analysis on the DE data to uncover which biological processes, molecular functions and KEGG pathways are enriched following a reduction in culture temperature. Ubiquitinated peptide enrichment was then carried out on the digested whole cell lysates, and DE and GO analysis performed on ubiquitinated peptides. With this first in‐depth ubiquitinated proteomic study on hypothermia induced ER‐stress mechanisms in CHO cells, we aim to improve our understanding of the role of ubiquitination in ER stress mechanisms, as well as protein folding processes, and highlight new possible targets for genetic engineering of CHO cells to enhance productivity and make cells more resilient to changes in culture conditions. While previous research has characterised global proteomic and transcriptomic changes in CHO cells subjected to temperature shifts, the specific impact of protein ubiquitination remains largely unexamined. By focusing on ubiquitin‐mediated regulation, this study provides new insights into posttranslational modifications that may underpin cellular adaptation and productivity changes during bioprocessing.

## 2 Methodology

### 2.1 Cell Culture Conditions

CHO‐K1 (ATCC CCL‐61), CHO‐DP12 cell line (ATCC CRL‐12445 clone #1934) which produces an anti‐IL8‐IgG, and CHO‐SK15‐EPO (an in‐house CHO‐K1 (ATCC CCL‐61) derived cell line, expressing recombinant human erythropoietin (EPO) in a pcDNA3.1 vector (Invitrogen) modified with puromycin resistance as selection system) (Costello et al. 2019) were grown at 37°C under 5% CO_2_ and 80% humidity at 170 rpm in BalanCD CHO Growth A (FujiFilm, 91128) cell culture medium supplemented with 4 mM l‐glutamine (Sigma Aldrich, G7513) in a Climo‐Shaker (Kühner). Cells were cultured in a working volume of 5 mL of cell culture media in 50 mL TPP® TubeSpin BioReactor Tubes (Merck, Z761028‐180EA), with routine passaging at 2 × 10^5^ cells/mL. The cells were routinely tested in‐house for Mycoplasma contamination and were found to be negative. CHO‐SK15‐EPO cultures were pulsed with 10 mg/mL puromycin (Sigma‐Aldrich/Merck, P8833) and CHO‐DP12 with 400 nM MTX every 2–3 weeks to ensure only producing cells remained in culture.

### 2.2 Temperature Shift Culture Conditions

CHO‐K1, CHO‐DP12, and CHO‐SK15‐EPO cells were seeded at 2 × 10^5^ cells/mL in triplicate tubes, cultured as described above and assayed using the COUNTESS II (Invitrogen, Thermo Scientific) and trypan blue staining (Invitrogen, Thermo Scientific) daily to collect growth and viability data. After 2 days, a subset of samples was transferred into an additional Kuhner incubator set at 31°C. Cell pellets for MS analysis were collected on day 4 and day 7 from the triplicate cultures from each of the three cell lines, and media samples for titre evaluation using ELISA were collected every second day, from separate triplicate cultures used for each measurement, so cell number or media volume was not impacted by sampling.

### 2.3 ELISA

hEPO titre from CHO‐SK15‐EPO cells and IgG titre from CHO‐DP12 cells was measured using ELISA, as previously described (Ryan et al. 2023).

Specific productivity (pg/cell/day) was calculated as follows ‐ productivity per viable cell on second time point minus productivity per viable cell on first time point, all divided by difference between time points.

Specificproductivity(pg/cell/day)=(((P1−P0)/(((X1−X0)/2))))/(T1−T0)

Where,

P0 = productivity (pg/mL) by first point of analysis

P1 = productivity (pg/mL) by second point of analysis

X0 = Viable cell density (×10^6^ cells/mL) by first point of analysis

X1 = Viable cell density (×10^6^ cells/mL) by second point of analysis

T0 = Day of first point of analysis

T1 = Day of second point of analysis

### 2.4 Protein Extraction From Whole Cell Lysates

Cells were collected and pelleted at 3 × 10^6^ cells each, in triplicate, with three PBS wash steps between pelleting by centrifugation, then snap frozen and stored at −80°C. Each sample was prepared for LC‐MS/MS using the PreOmics iST kit, essentially as per manufacturer's instructions (PreOmics, P.O.00001). 100 µg of cell material was lysed with the lysis buffer (Preomics) at 95°C for 10 min at 1000 rpm. Following this, the Trypsin/LysC mix was added (as supplied in the iST kit), and the sample incubated at 37°C for 3 h at 500 rpm to allow proteolytic digestion to take place. Once the digestion was stopped, the peptide samples were washed and filtered through the cartridge. Once filtered, the peptide samples were placed in the vacuum evaporator at 45°C until fully dry. Dried peptide samples were then stored at −80°C.

### 2.5 Ubiquitinated Peptide Enrichment

A 1.5 mL volume of 8 M urea, 50 mM Tris, 100 mM NaCl (pH 8.5) buffer was used to lyse cells followed by sonication using an ultrasonicator with 30 s sonication pulses (×3 cycles) per sample, on ice. Cell debris was removed by centrifugation of the sample at 14,000×g for 10 min at 4°C. The supernatant was collected, and the protein concentration was measured using the Bradford Assay (Thermo Scientific) at 660 nm. Five milligrams of protein from each sample is required for enrichment. Protein samples were adjusted to a volume of 1 mL with 50 mM ammonium bicarbonate and 0.04% ProteaseMax (Promega). Samples were reduced at a final concentration of 5 mM DTT (Sigma‐Aldrich/Merck) at 56°C, at 500 rpm for 30 min. Samples were cooled and iodoacetamide (IAA) (Cell Signalling Technology) was added to a final concentration of 25 mM and incubated at room temperature in the dark, at 500 rpm for 1 h. Samples were then diluted to achieve a final concentration of urea < 1.6 M. Lys‐C (Cell Signalling Technology) was added at a 1:200 (w/w) ratio and incubated for 4 h at 37°C, with mixing every 30 min. Trypsin (Thermo Scientific) was added at a 1:100 (w/w) ratio and incubated for 18 h at 37°C.

Before beginning C‐18 clean up, each sample was acidified using Trifluoroacetic Acid (TFA) at a 0.5% final concentration, giving a pH ~ 2. C‐18 columns (Sep Pak, Waters) were used as per manufacturer's instructions. Columns were activated with 5 mL of 100% acetonitrile (ACN) (Thermo Scientific), then equilibrated with 0.1% TFA. Sample was then added, using 1 mL of 0.1% TFA to rinse the incubation tube. Elution was performed with 50% ACN:0.1% TFA, first with 4 mL, then with 2 mL. Eluates were flash frozen, and then vacuum dried over 24 h.

Magnetic bead immunoaffinity precipitation was completed using the PTMScan® HS Ubiquitin/SUMO Remnant Motif (K‐ε‐GG) Kit (Cell Signalling Technology) as per manufacturer's instructions. In a deviation from the manufacturer's protocol, C‐18 spin columns (Pierce, Thermo Scientific) were substituted for C‐18 tips for greater certainty that the peptide amount is within the binding capacity of the column and used per manufacturer's instructions. After obtaining an eluate, the process was repeated with the remaining peptide solution to ensure the most peptides possible were obtained. The combined eluates were dried using a Speed‐Vac for approximately 2 h, until entirely dried.

### 2.6 LC‐MS/MS for Label Free Quantification (LFQ)

For LC‐MS/MS analysis, an UltiMate 3000 nano RSLC (Thermo Scientific) system interfaced with an Orbitrap Fusion Tribrid Mass Spectrometer (Thermo Scientific) was used. Using nano‐flow HPLC separation, 1 µg of tryptic peptide from each sample was loaded onto the trapping column (PepMap100, C18, 300 μm × 5 mm) at a flow rate of 25 μL/min with 2% (v/v) ACN, 0.1% (v/v) TFA for a 3 min desalting and concentration step. Each sample was then resolved onto an analytical column (Acclaim PepMap 100, 75 μm × 50 cm, 3 μm bead diameter column) into the mass spectrometer. A binary gradient of: solvent A (0.1% (v/v) formic acid in LC‐MS grade water) and solvent B (80% (v/v) ACN, 0.08% (v/v) formic acid in LC‐MS grade water) using 2%–32% B for 75 min, 32%–90% B in 5 min and holding at 90% for 5 min at a flow rate of 300 nL/min was used to elute peptides.

For MS and MS/MS analysis, a temperature of 320°C and a voltage of 2.0 kV was used for peptide ionisation. The Orbitrap mass analyser with a resolution of 120,000 (at m/z 200), a maximum injection time of 50 ms and an automatic gain control (AGC) value of 4 × 10^5^ was used to perform full MS scans for peptide mass information. Data‐dependent acquisition was performed using a full scan range of 380–1500 m/z. A top‐speed MS/MS acquisition algorithm was used to determine the number of selected precursor ions for fragmentation. A dynamic exclusion was applied to the analysed peptides after 60 s and peptides with a charge state between 2+ and 7+ were analysed. Peptides were fragmented using higher energy collision‐induced dissociation (HCD) with a normalised collision energy of 29%, with a maximum injection time of 35 ms and an AGC value of 2 × 10^4^. The resulting MS/MS fragment ions were measured in the low‐resolution linear ion trap.

### 2.7 Protein Identification and Differential Expression Analysis Using Quantitative Label‐Free LC‐MS/MS

Quantitative label‐free analysis was performed using Progenesis QI for Proteomics to compare cells cultured at 31°C to 37°C on day 4 and day 7. Only peptide ions with charge states +2 and +3 were allowed and normalised. The normalised peptide abundance data was transformed before statistical analysis, using an arcsinh transformation to meet the assumptions of the one‐way ANOVA test. Peptides with a one‐way ANOVA *p*‐value ≤ 0.05 between experimental groups were exported and identified using Proteome Discoverer as described. Protein identifications were imported into Progenesis and considered differentially expressed proteins if they passed the following criteria: (i) a protein level one‐way ANOVA *p*‐value ≤ 0.05 between experimental groups, (ii) a minimum of 1 peptide contributing to a protein identification and (iii) a minimum fold change in abundance of ± 1.5‐fold between culture temperatures.

Peptides were identified using Proteome Discoverer 2.5 (Thermo Scientific) against a *Cricetulus griseus* UniProt database (downloaded September 2022) using the SEQUEST HT algorithm and exported to Progenesis QI for Proteomics (NonLinear Dynamics, a Waters Company, UK) for relative quantitative analysis between experimental groups.

The following search parameters were set for protein identifications: (i) MS/MS mass tolerance set at 0.6 Da; (ii) peptide mass tolerance set to 10 ppm; (iii) trypsin as the enzyme; (iv) up to two missed cleavages were allowed; (v) cysteine carbamidomethylation as a static modification and (vi) methionine oxidation as a dynamic modification.

### 2.8 LC‐MS/MS of Ubiquitinated Peptides and Identification of Differentially Expressed Ubiquitinated Proteins

Ubiquitinated peptide LC‐MS/MS and identification was carried out as previously described (Selvaprakash et al. 2024). 1 µg of ubiquitinated peptides from each sample were loaded onto the trapping column at a flow rate of 25 µL/min with 2% (v/v) ACN and 0.1% (v/v) TFA for 3 min. Samples were resolved in the analytical column using a binary gradient of formic acid solution and ACN/formic acid solution using a flow rate of 280 nL/min to elute peptides. Data‐dependent acquisition was used for all MS runs, using a full scan range of 380–1500 *m*/*z*, over 140 min. MS1 spectra were collected at a resolution of 120,000 with an AGC target of 2 × 10^5^ with a maximum injection time of 50 ms. Dynamic exclusion was applied to analyse peptides after 60 s, and peptides with a charge state between 2+ and 7+ were analysed. Peptides were fragmented using higher energy collision‐induced dissociation with a collision energy of 29%, an AGC value of 1 × 10^5^, and a maximum injection time of 90 ms in the ion trap. Peptides were identified using Proteome Discoverer 2.5 (Thermo Scientific) against a *Cricetulus griseus* UniProt database (downloaded September 2022) using the SEQUEST HT algorithm and exported to Progenesis QI for Proteomics for relative quantitative analysis between experimental groups. The following search parameters were set for protein identifications: (i) MS/MS mass tolerance set at 0.6 Da; (ii) peptide mass tolerance set to 10 ppm; (iii) trypsin as the enzyme; (iv) up to two missed cleavages were allowed; (v) cysteine carbamidomethylation as a static modification; (vi) methionine oxidation as a dynamic modification; and (vii) lysine with a K‐ε‐GG remnant (114.0429 Da) as a variable modification. Protein identifications were imported into Progenesis and considered differentially expressed proteins if they passed the following criteria: (i) a protein level one‐way ANOVA *p*‐value ≤ 0.05 between experimental groups, (ii) a minimum of 1 ubiquitinated peptide contributing to a protein identification and (iii) a minimum fold change in abundance of ± 1.5‐fold between culture temperatures.

### 2.9 KEGG Pathway and STRING Association Analysis

Whole cell proteins which were differentially expressed between control and test samples were inputted into STRING (https://www.string-db.org) (Szklarczyk et al. 2021), which was used to group the proteins based on protein‐protein interactions and to provide groupings within KEGG biological pathways (https://www.genome.jp/kegg) (Kanehisa et al. 2023). The differentially expressed ubiquitinated proteins were also analysed using this same methodology.

### 2.10 Graph Plotting

All data plotting was performed using R, https://www.R-project.org/, package *ggplot2* (Wickham 2016).

## 3 Results and Discussion

### 3.1 Culture Viability and Recombinant Protein Production

**Figure 1:**
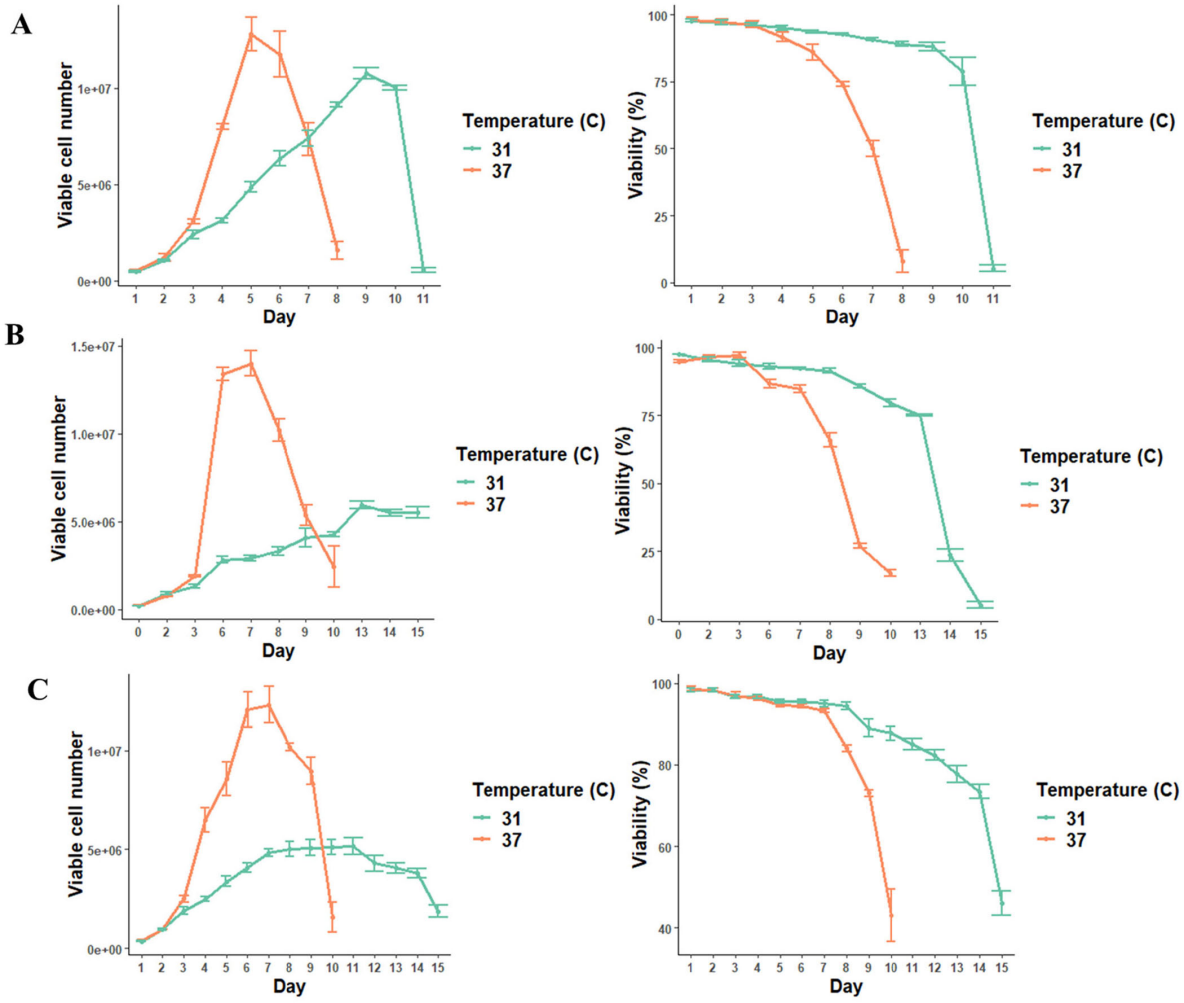
Viable cell density (cells/mL) and percentage cell viability for (A) CHO‐K1, (B) CHO‐DP12 and (C) CHO‐SK15‐EPO cells cultured at 37°C and 31°C, where culture temperature downshift occurs at 48 h post cell seeding. In all plots, orange represents 37°C, and blue represents 31°C. The data lines are the mean value of triplicate results, with error bars representing the standard deviation from the mean value at each time point.

A reduction in CHO culture temperature is regularly used in the biopharma industry after the initial growth phase to increase productivity of cells or enhance product quality attributes of the recombinant protein or antibody (Torres et al. 2021). In this study, non‐producer (CHO‐K1) and producer (CHO‐SK15 EPO and CHO DP‐12) CHO cells were exposed to mild hypothermic conditions, and the ER stress response was analysed at the proteomic level. These cell lines allow for comparison between the native stress response in CHO‐K1and the stress response in cells engineered for recombinant protein production (hEPO from CHO‐SK15‐EPO) or a more complex antibody (human IgG1 from CHO‐DP12). Previous studies have reported on the lowering of culture temperatures from 37°C to between 35°C and 31°C. In our study we reduced the culture temperature to 31°C as this was the most extreme condition observed in these previous studies, in pursuit of the most comprehensive view of ER stress induced by this method. Previous studies have also indicated that a moderate shift to 35°C decreased final titre relative to the unshifted control whereas a temperature shift to 32°C can significantly increase final titre (McHugh et al. 2020), suggesting a greater temperature change can be more beneficial to productivity. Enhanced productivity in CHO cells has been linked to pathways involving mRNA stability (Oguchi et al. 2006), cell cycle arrest (Chen et al. 2004), and protein secretion (Yee et al. 2009), with additional studies implicating reduced apoptosis (Moore et al. 1997), increased secretory capacity (Smales et al. 2004), and elevated recombinant gene transcription (Kou et al. 2011). Figure 1 shows the viable cell count and viability for the three cell lines studied, where cultures maintained at 37°C show an increase in cell density at a faster rate than cells cultured at 31°C, and reach a greater peak viable cell density than the cells cultured at 31°C, CHO‐K1 between days 3–5, and between days 5–7 in CHO‐DP12 and CHO‐SK15‐EPO. CHO‐K1 starts to show a steady reduction in viability at 37°C by day 3 of culture, while at 31°C, there is a more gradual reduction over the culture period, with a noticeable drop only seen at day 9. Both CHO‐DP12 and CHO‐SK15‐EPO cultures show a slower decline in percentage viability at 37°C at the earlier stages of culture, but a more rapid decline in later stages, in particular in CHO‐SK15‐EPO, where viability drops from 93% at day 7 to 43% at day 10. Viability remains steady in both producer cell lines at 31°C until later stages of culture, with a far more gradual decline than the rapid decline seen at 37°C. The two producer cell lines reach a peak viable cell count of approximately 5 × 10^6^ cells/mL at 31°C, less than half of the count reached by the 37°C cultures. These results align with previous studies, with all three cell lines maintaining culture viability for longer at 31°C than at 37°C, but with a much‐reduced growth rate (Goey et al. 2017; Smales et al. 2004; Torres et al. 2021).

**Figure 2:**
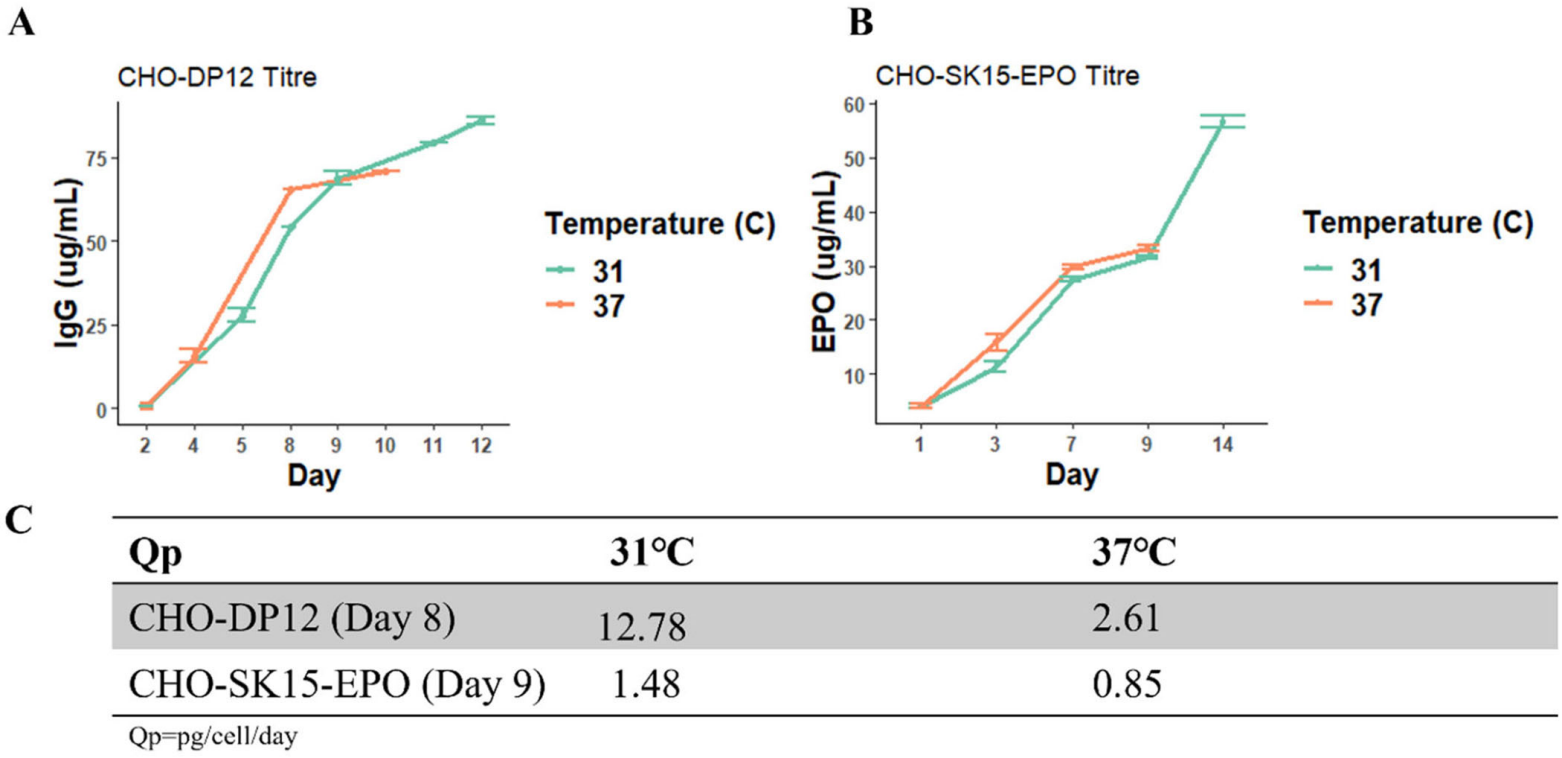
(A) IgG titre from CHO‐DP12 cells cultured at 31°C and 37°C, (B) hEPO titre from CHO‐SK15‐EPO cells cultured at 37°C and 31°C, and (C) specific cell productivity from both cell lines measured at a later time point in the 37°C‐culture lifespan. Titre was measure by means of ELISA assay. In all plots, orange represents 37°C, and blue represents 31°C. The data lines are the mean value of triplicate results, with error bars representing the standard deviation from the mean value at each time point.

The different culture temperatures can also affect productivity in the two producer cell lines, as shown in Figure 2. Although product titre is initially higher in cultures maintained at 37°C, the extended viability of cells at 31°C ultimately results in a greater final titre following the temperature shift. Comparisons of the specific cell productivity (Qp) show that cells subjected to the temperature shift are producing more recombinant protein than cells cultured at 37°C, with CHO‐DP12 having a 4.4‐fold increase in Qp at 31°C, and CHO‐SK15‐EPO having a smaller 1.7‐fold increase at 31°C. The increase in productivity as a result of lowering the culture temperature is in line with previous hypothermia studies of CHO cells (Sou et al. 2015; Torres et al. 2021; Yoon et al. 2003), with previous publications suggesting that this increase may be due to increased transcriptional activity (Smales et al. 2004) or enhanced mRNA stability (Nguyen et al. 2020).

### 3.2 Gene Ontology Analysis of Differentially Expressed Proteins in CHO Cells Under Mild Hypothermic Conditions

In this study, whole‐cell proteome analysis was performed on the three CHO cell lines subjected to a temperature shift from 37°C to 31°C to investigate temperature‐dependent variations in protein abundance. Samples were collected at two time points, day 4 and day 7, to study changes over the culture period. Quantitative label‐free mass spectrometry was used to compare proteomic profiles, with the aim to gain insights into how culture temperature impacts culture viability and productivity. The total number of differentially expressed proteins for each cell line is shown in Table 1. All differentially expressed proteins are listed in Supporting Materials 1.

**Table 1:** The number of proteins identified to be upregulated at each culture temperature on day 4 and day 7, in nonproducing cell line CHO‐K1, hEPO producer CHO‐SK15‐EPO and IgG producer CHO‐DP12.

	CHO‐K1		CHO‐SK15‐EPO		CHO‐DP12	
	31°C	37°C	31°C	37°C	31°C	37°C
**Day 4**	526	380	347	514	561	547
**Day 7**	650	624	408	420	650	624

*Note:* Proteins identified show at least 1.5‐fold change between conditions, an ANOVA *p*‐value of less than or equal to 0.05 and are identified by at least one peptide matching the database sequence.

The differentially expressed proteins were subjected to GO analysis, focusing on KEGG pathways that are enriched in these proteins. All enriched KEGG pathways for all three cell lines can be found in Supporting Materials 2 (Table 1). At both time points, metabolism‐related pathways including, ‘Metabolic pathways’ (cge0110), ‘Carbon metabolism’ (cge01200) and ‘Purine metabolism’ (cge0230), are shown to have changes in protein expression following a reduction in culture temperature, indicating that culture temperature affects how cells metabolise nutrients, in particular carbon, which may account for the reduction in growth. It was observed that a higher number of differentially expressed proteins mapped to the ‘Proteasome’ (cge03050) pathway at day 7 than day 4, with this pathway not showing up as enriched in CHO‐SK15‐EPO on day 4. The majority of these proteins showed an increase in levels of abundance on day 7 in the temperature shifted cultures, suggesting there are more misfolded proteins being degraded in these cultures. This is most likely due to the cells at the lower temperature producing more of the recombinant protein, whereas at 37°C cell viability is starting to decrease, so cells are likely producing less recombinant protein than earlier in culture. ‘Apoptosis’ (cge04210) is also enriched, suggesting some cells are unable to restore ER homeostasis and apoptotic cell death is triggered.

‘Protein processing in Endoplasmic reticulum’ (cge04141) is a KEGG pathway which maps the proper functioning and homeostasis of the ER in CHO cells via multiple mechanisms such as the UPR, ERAD and proteasomal degradation. This pathway appears enriched at both time points in all three cell lines. This pathway is one of the main focuses of this study as the differentially expressed proteins allow for investigation of both the normal folding and quality control process, as well as those involved in the ER stress response. This allows for a greater focus on ER proteins that may otherwise be overlooked if a broader scope study was undertaken, looking at all differentially expressed proteins. The number of differentially expressed proteins for each cell line at day 4 and day 7 which contribute to the enrichment of this pathway are shown in Table 3, along with the strength and false discovery rate associated with this pathway according to STRING analysis. The KEGG pathways ‘Apoptosis’ (cge04210) is also enriched at day 4 in all three cell lines, with proteins related to caspase‐mediated apoptosis differentially expressed (BAK1, EIF2α, CASP7), suggesting that cells where ER homeostasis cannot be restored will undergo apoptosis via these specific pathways.

### 3.3 Gene Ontology Analysis of Differentially Expressed Ubiquitinated Proteins in CHO Cells Under Mild Hypothermic Conditions

The ubiquitinated proteome represents an under investigated aspect of the CHO proteome. The importance of ubiquitination in various cell processes, in particular relating to protein production, makes it a promising area for new insights into CHO productivity. Studies have shown that using targeted miRNAs to affect levels of some ubiquitin‐proteasome related proteins has phenotypic effects on growth (Costello et al. 2018) and productivity (Fischer et al. 2015). Following tryptic digestion of proteins, a Lys‐ε‐diglycine (diGly) remnant is left on a peptide, indicating a site of ubiquitination. This tag has become the target for an immunoaffinity‐based methods of enrichment of ubiquitinated peptides (using an anti‐K‐ε‐GG antibody), allowing these peptides to be detected using mass spectrometry (van der Wal et al. 2018). This is the method used here to investigate the effects of temperature shift on the ubiquitinated proteome of non‐producer CHO‐K1, hEPO producer CHO‐SK15‐EPO and IgG producer CHO‐DP12. Differentially expressed ubiquitinated proteins are defined here as protein identified by at least one peptide with a diGly remnant matching the database sequence, at least 1.5‐fold change between conditions and an ANOVA *p*‐value of less than or equal to 0.05. The number of proteins matching these parameters for each cell line at day 4 and day 7 is listed in Table 3. Complete lists of all differentially expressed ubiquitinated proteins can be found in Supporting Materials 3.

These proteins map to many cellular pathways, functions and processes, including some that are not identified as enriched in the whole cell proteome data. Complete lists of these enriched features are available in Supporting Materials 2 (Table 2). The KEGG pathways, molecular functions and processes that are shared between the cell lines on day 4 and day 7 are listed in Table 4. On day 4, processes related to cellular organisation may hint at the cells attempt to reorganise its functions in response to the shock of the downshift in culture temperature. On day 4, the ‘Proteasome’ (cge03050) pathway is enriched in differentially expressed ubiquitinated proteins in both producer lines CHO‐SK15‐EPO and CHO‐DP12. This suggests that both these cell lines use ubiquitination in the regulation of their response to the ER stress induced by temperature downshift, given the crucial role played by the proteasome in degradation of misfolded proteins (Roos‐Mattjus and Sistonen 2004). On day 7, this pathway is enriched in differentially expressed ubiquitinated proteins in CHO‐SK15‐EPO and CHO‐K1, but not in CHO‐DP12, suggesting that CHO‐DP12 no longer requires such tight regulation of this pathway, and that CHO‐K1 now needs greater control of proteasomal activity than at day 4.

**Table 2:** Comparison of the number of differentially expressed proteins which contribute to the enrichment of the KEGG Pathway: ‘Processing in Endoplasmic Reticulum’ (cge04141).

	Count	Strength	False discovery rate
**Day 4**			
CHO‐K1	24/128	0.7	2.84e‐08
CHO‐SK15‐EPO	27/128	0.77	4.45e‐10
CHO‐DP12	27/128	0.66	2.12e‐08
**Day 7**			
CHO‐K1	33/128	0.69	1.25e‐08
CHO‐SK15‐EPO	22/128	0.7	7.06e‐07
CHO‐DP12	33/128	0.81	1.79e‐13

**Table 3:** The number of ubiquitinated proteins identified to be upregulated at each culture temperature on day 4 and day 7, in nonproducing cell line CHO‐K1, hEPO producer CHO‐SK15‐EPO and IgG producer CHO‐DP12.

	CHO‐K1		CHO‐SK15‐EPO		CHO‐DP12	
	31°C	37°C	31°C	37°C	31°C	37°C
**Day 4**	76	209	124	405	33	85
**Day 7**	423	58	91	25	28	27

**Table 4:** The KEGG pathways, molecular functions and processes enriched in ubiquitinated differentially expressed proteins shared between CHO‐K1, CHO‐SK15‐EPO and CHO‐DP12 on day 4 (A) and day 7 (B).

A							
		CHO‐SK15‐EPO			CHO‐DP12		
KEGG	Description	Count	Strength	FDR	Count	Strength	FDR
cge05012	Parkinson disease	7	0.87	0.02	9	1.01	4.51E‐05
cge04520	Adherens junction	4	1.2	0.0271	3	1.1	0.0432
cge04810	Regulation of actin cytoskeleton	6	0.88	0.0271	5	0.83	0.0246
cge05014	Amyotrophic lateral sclerosis	7	0.69	0.0271	9	0.82	0.00046
cge03050	Proteasome	3	1.19	0.0295	8	1.65	1.44E‐08

		CHO‐K1			CHO‐SK15‐EPO			CHO‐DP12		
Term ID	Function	Count	Strength	FDR	Count	Strength	FDR	Count	Strength	FDR
GO:0005488	Binding	293	0.13	1.20E‐11	111	0.19	2.28E‐11	98	0.17	1.76E‐06
GO:0005515	Protein binding	183	0.17	2.64E‐07	79	0.3	1.32E‐09	67	0.25	2.50E‐05
GO:0097159	Organic cyclic compound binding	140	0.15	0.00057	55	0.24	0.0051	52	0.24	0.0041
GO:1901363	Heterocyclic compound binding	139	0.16	0.00057	54	0.23	0.0067	52	0.24	0.0035
GO:0019899	Enzyme binding	67	0.24	0.0018	34	0.43	6.93E‐05	27	0.36	0.0167

		CHO‐K1			CHO‐SK15‐EPO			CHO‐DP12		
Term ID	Process	Count	Strength	FDR	Count	Strength	FDR	Count	Strength	FDR
GO:0009987	Cellular process	322	0.1	110	15994	0.12	0.00026	106	0.13	7.61E‐06
GO:0016043	Cellular component organization	132	0.17	49	5621	0.22	0.0279	48	0.24	0.007
GO:0071840	Cellular component organization or biogenesis	137	0.16	52	5887	0.23	0.0146	51	0.25	0.0031
GO:0006996	Organelle organization	92	0.2	39	3632	0.32	0.0098	41	0.37	0.00017

B										
		CHO‐K1			CHO‐SK15‐EPO			CHO‐DP12		
KEGG	Description	Count	Strength	FDR	Count	Strength	FDR	Count	Strength	FDR
cge04530	Tight junction	16	0.88	3.36E‐07	5	0.99	0.0328	6	1.44	3.44E‐05
cge05012	Parkinson disease	16	0.64	9.61E‐05	8	0.95	0.0015	4	1.03	0.0193
cge04670	Leukocyte transendothelial migration	10	0.78	0.00042	4	0.99	0.0328	4	1.37	0.0034
cge04971	Gastric acid secretion	6	0.77	0.0071	3	1.08	0.0498	3	1.46	0.0124
cge04810	Regulation of actin cytoskeleton	9	0.47	0.0226	5	0.82	0.0328	4	1.11	0.0143
cge05034	Alcoholism	7	0.51	0.0299	4	0.88	0.0498	4	1.26	0.0066

		CHO‐K1			CHO‐SK15‐EPO			CHO‐DP12		
Term ID	Function	Count	Strength	FDR	Count	Strength	FDR	Count	Strength	FDR
GO:0005488	Binding	400	0.16	1.62E‐29	96	0.15	2.95E‐05	44	0.19	0.003
GO:0005515	Protein binding	254	0.22	1.56E‐17	69	0.26	9.24E‐06	30	0.28	0.0194
GO:0019899	Enzyme binding	104	0.33	4.66E‐11	30	0.4	0.0011	16	0.51	0.014

		CHO‐K1			CHO‐SK15‐EPO			CHO‐DP12		
Term ID	Process	Count	Strength	FDR	Count	Strength	FDR	Count	Strength	FDR
GO:0032879	Regulation of localization	108	0.37	5.99E‐14	29	0.41	0.0021	16	0.53	0.0067
GO:0051179	Localization	165	0.26	4.58E‐13	45	0.31	0.0017	22	0.38	0.0144
GO:0051234	Establishment of localization	146	0.28	3.63E‐12	42	0.35	0.00072	21	0.43	0.0071
GO:0008104	Protein localization	91	0.37	1.81E‐11	24	0.41	0.0147	16	0.61	0.0025
GO:0006810	Transport	137	0.27	7.83E‐11	39	0.34	0.0021	20	0.43	0.0106
GO:0045184	Establishment of protein localization	63	0.43	1.07E‐09	17	0.47	0.0358	12	0.7	0.0044
GO:0051641	Cellular localization	106	0.3	2.06E‐09	NA	NA	NA	17	0.49	0.0092
GO:0051049	Regulation of transport	79	0.31	2.12E‐07	24	0.41	0.0134	13	0.52	0.0389

*Note:* No KEGG pathways were enriched in CHO‐K1 at day 4.

On day 7, all cell lines show several enriched processes related to protein localisation in the cell, as well as protein transport. This may be linked to the localisation of proteins to the ER as part of the UPR, or the movement of ubiquitinated proteins out of the ER and suggests that the organisation within the cell is more established and requires less regulation than at day 4. While none of the shared KEGG pathways at day 7 link to ER stress, the prevalence of protein binding as a shared molecular function could be attributed to stress, as many UPR and ERAD proteins are activated by either the binding or un‐binding of other proteins. Binding functions are also shared at day 4, further suggesting the role of binding in the ER stress response.

In all three cell lines, KEGG pathways, biological processes and molecular functions related to protein localisation and cellular organisation are enriched in ubiquitinated protein DE data. This suggests the importance of ubiquitin in the regulation of fundamental cell processes in mild hypothermic conditions. The biological processes “Cellular component organisation”, “Cellular component organisation or biogenesis”, and “Organelle organisation” (GO:0016043, GO:0071840, GO:0006996) were enriched in all three cell lines on day 4. These show the extent of ubiquitination in key cellular functions, as the cells adapt following temperature downshift. These processes are not found across all cell lines at day 7, only in CHO‐K1. This suggests that cells may undergo some rearrangement of organelles and other subcellular components in response to mild hypothermia. A reduction in culture temperature has been shown to arrest cells in the G1 phase of the cell cycle (Becerra et al. 2012), which is the phase of the cell cycle during which the cell grows, copies organelles and accumulates nutrients (Csikász‐Nagy et al. 2006). The interruption of these processes may require ubiquitin as a crucial regulatory factor in this process or to target the proteins no longer needed in these processes for degradation. A study has found that 64% of cells remain in the G1 phase following a temperature downshift (Shahabi et al. 2023). This correlates with the reduction in cell replication, suggesting that cells arrested in the G1 phase may focus resources on protein production rather than cell replication, with producer cells focusing on recombinant protein production.

Protein localisation is another essential biological process that is seen to be enriched in the differentially expressed ubiquitinated proteins. The movement of proteins to the relevant subcellular location is crucial for correct cell function. Cellular signalling pathways are highly dependent on ubiquitin for responses to extracellular influences, and have been shown to modulate growth, cell cycle regulation, receptor function, development and the stress response (Wilkinson 1999). A 2011 review by Grabbe et al. highlighted the critical role of ubiquitin in regulating protein activity and localisation over time. Using the NF‐κB pathway as an example, the review demonstrated how different ubiquitin species coordinate the activation of kinase complexes across distinct branches of the pathway (Grabbe et al. 2011). The enrichment of localisation pathways would indicate that the effect of an external temperature downshift requires changes in the cellular organisation, and those changes require mediation by ubiquitination.

### 3.4 Differential Expression of Ubiquitinated Proteins Following a Downshift in Culture Temperature

In all three cell lines, at both time points, there are multiple proteins found in the whole cell proteome data that do not show up as ubiquitinated in that data but are found to be ubiquitinated in the enriched data, as shown in Table 5. All shared proteins can be found in Supporting Materials 1. These proteins come from a range of cellular pathways, indicating the expansive role ubiquitination plays in the proteome. It also highlights the transient nature of some proteins, with their function only required for a brief period, then being targeted for degradation. Pathways relating to proteasome function (KEGG: ‘Proteasome’, Cellular component: ‘Proteasome complex, Proteasome regulatory particle’, Process: ‘Proteasomal protein catabolic process’) are enriched in common between whole cell and ubiquitinated DE proteins of CHO‐DP12 and CHO‐SK15‐EPO, highlighting the role of ubiquitin in the regulation of ubiquitin‐mediated degradation. Across all cell lines, essential biological processes, including cellular metabolic process (‘GO:0044237’) and primary metabolic process (‘GO:0044238’), show the crucial role of ubiquitin in basic cellular function, and also demonstrates how standard proteomic studies can miss the post‐translation modifications present on detected proteins that are essential to normal cell function.

**Table 5:** The number of shared proteins between the whole cell proteome differentially expressed data and the ubiquitinated differentially expressed data, as well as the number of these proteins that are oppositely regulated between the data.

Day 4			Day 7		
CHO‐K1	CHO‐SK15‐EPO	CHO‐DP12	CHO‐K1	CHO‐SK15‐EPO	CHO‐DP12
48	105	34	163	7	19
24	55	3	45	5	9

*Note:* The presence of the double‐glycine ubiquitin protein remnant was detected on none of these proteins in the whole cell proteome data, only when the enriched peptides were analysed.

Only a minority of these common proteins are found at both day 4 and day 7 in the respective cell lines, so it is likely that those found at day 4 only are proteins that are no longer needed in cell activity and are then degraded. CHO‐K1 shows the greatest number of shared ubiquitinated differentially expressed proteins between timepoints, suggesting the need for a long‐term response to hypothermic stress than that seen in either of the producer cell lines. CHO‐K1 also shows the most differentially expressed proteins in common between the whole cell proteome and ubiquitinated proteome on day 7, more than on day 4, whereas in both producer cell lines the number of shared ubiquitinated proteins decreases between the time points. This may be suggestive of a lesser need for long term ubiquitin‐mediated degradation and regulation in the producer cell lines, suggesting a greater ability of the ER stress response in these cells to restore ER homeostasis than the nonproducing CHO‐K1 line.

There is a subset of these proteins where they are oppositely regulated between the whole cell and ubiquitinated data sets, the number of which is also included in Table 5. These proteins have a range of biological functions, and from a range of cellular locations. This suggests that these proteins exist within the cell in both ubiquitinated and non‐ubiquitinated states, again highlighting the role of ubiquitin in the regulation of biological processes, and that the differing forms of these proteins may serve different biological functions. GJA1 is one of such differentially regulated proteins, being differentially regulated in both producer cell lines on day 4 (CHO‐DP12: WCP = +5.1, Ubi = −1.5; CHO‐SK15‐EPO: WCP = −1.5, Ubi = +24.5). This gap junction protein has been linked to protein localisation (Bazzoun et al. 2019), cell‐cell signalling and signal transduction (Fishman et al. 1990). It has been found that the ubiquitination of GJA1 is crucial to its degradation via autophagy, with E3 ligase NEDD4 being responsible for this ubiquitination (Bejarano et al. 2012). NEDD4 is found to be upregulated at 31°C in both CHO‐K1 and CHO‐DP12.

The greatest difference in expression is in reticulocalbin in CHO‐SK15‐EPO, on day 4. Downregulated in the whole cell proteome data (−1.52‐fold) and upregulated in the ubiquitinated data (+30.73‐fold), reticulocalbin is a calcium‐binding protein located in the lumen of the ER (Weis et al. 1994). This suggests that while reticulocalbin is more abundant at 37°C, it predominantly exists in a non‐ubiquitinated form. In contrast, when focusing solely on the ubiquitinated form, its levels are higher at 31°C, indicating temperature‐dependent regulation of its posttranslational modification. Proteins exhibiting similar fold‐change patterns in both whole‐cell and ubiquitinated datasets may indicate that ubiquitination occurs uniformly across all protein molecules. In such cases, the observed changes in the ubiquitinated form likely reflect shifts in total protein abundance, rather than an increase in the extent of ubiquitination per molecule. The proteins that are regulated in the same direction in both data sets can also show some large differences in abundance. Plectin shows the largest difference, with a 68‐fold increase at 31°C of the ubiquitinated form in CHO‐K1 on day 7. This protein is part of the plakin family, which involved in maintaining cell integrity but also serve as scaffolding platforms for the regulation of signalling complexes (Sonnenberg and Liem 2007). Plectin has been studied in C2C12 myoblasts, where it was found to promote proliferation and inhibit apoptosis, and may act to inhibit ubiquitination of the Dvl‐2 protein, inhibiting autophagy (Yin et al. 2021). There is a lack of research on the ubiquitination of plectin itself, although studies of skin, muscle and nerve tissues suggest that phosphorylation of this protein is an important part of some of its functions (Castañón et al. 2013). This difference in expression can be explained by either differing functionality of the ubiquitinated and non‐ubiquitinated forms of the protein, or that at the time point studied, we are observing the end of the functional period of a protein, and so most copies of that protein are tagged for degradation.

### 3.5 Differential Expression of ER Stress Proteins Following a Downshift in Culture Temperature

**Figure 3:**
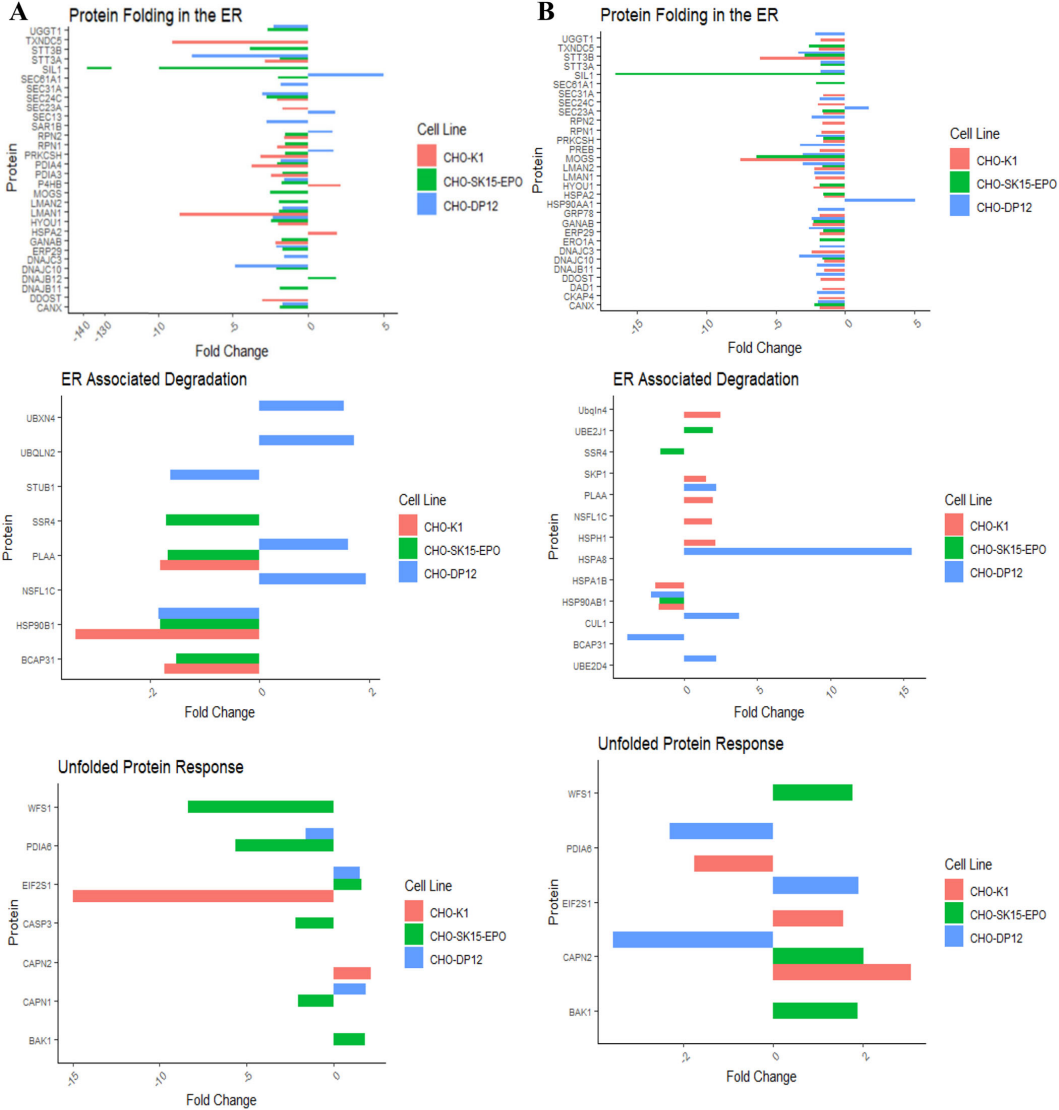
Fold changes of proteins in CHO‐K1 (pink), CHO‐SK15‐EPO (green) and CHO‐DP12 (blue) that are differentially expressed between cells culture at 31°C and cells cultured at 37°C, at (A) day 4 and (B) day 7. A minus value represents a protein that was less abundant in cells cultured at 31°C than in cells cultured at 37°C. In “Protein Folding in the ER” at day 4, where SEC. 61A1 is shown to have a +5‐fold change in CHO‐DP12, this value is for illustrative purposes, as this protein was only detected in the 31°C culture in this cell line, so has an “infinity” fold change between temperatures. In this same plot, an x‐axis break is employed to represent the −136.5‐fold change in the protein SIL1 in CHO‐SK15‐EPO, while keeping the other values within focus.

In this study, the pathway is further broken down into three areas, relating to the different mechanisms present within the ER, as detailed in our previous study (Ryan et al. 2023). These are *Protein Folding*; the ER function relating to protein synthesis, glycosylation, glucosylation and folding, *ERAD*; ER function related to the degradation of misfolded proteins and *Unfolded Protein Response*; ER function relating to the ER mechanism of coping with an accumulation of misfolded proteins. Figure 3 details the fold changes of the differentially expressed proteins in each of these three areas. The use of three different cell lines, an antibody producer, a protein producer and a non‐producer, shows the differing ER responses that can occur within cells of the same species, engineered for different purposes. The differences in cell line specific UPR and ERAD pathways may be part of the reason it has so far proven difficult to find a comprehensive remedy to ER stress across CHO cells.

#### 3.5.1 Protein Folding

Following protein translation, newly synthesised proteins enter the ER lumen, where these polypeptides undergo modifications such as glycosylation and disulfide bond formation, assisted by molecular chaperones and folding enzymes including BiP and several protein disulfide isomerases (PDI) (Okumura et al. 2015). Proper folding is critical for protein functionality, and misfolded proteins are targeted for degradation via the ERAD pathway to maintain cellular homeostasis. This processing ensures that only correctly folded proteins proceed to the Golgi apparatus for further maturation and secretion.

On day 4, there is down regulation of many proteins related to protein folding and synthesis at 31°C in CHO‐DP12, CHO‐K1 and CHO‐SK15‐EPO. This includes proteins involved in transport of nascent polypeptides into the ER. SEC. 61a1 is a component of the SEC. 61 channel‐forming translocon complex that mediates transport of signal peptide‐containing precursor polypeptides across the ER (Meacock et al. 2002) and is downregulated in CHO‐SK15‐EPO cells at 31°C, but is upregulated in CHO‐DP12 at 31°C. Other transport proteins, SEC. 24 C and SEC. 31α, are downregulated in CHO‐DP12 at 31°C. SEC. 13 is needed for vesicle biogenesis for transport of proteins out of the ER (B. L. Tang et al. 1997) and is upregulated at 31°C in CHO‐DP12, while SAR1B, which is a GTPase that functions in vesicle‐mediated ER to Golgi transport (Cutrona et al. 2013), is downregulated at 31°C. SIL1 is required for protein translocation and folding in the ER, and functions as a nucleotide exchange factor for the ER lumenal chaperone HSPA5 (Chung et al. 2002). At day 4, there is a prominent fold change in SIL1 in CHO‐SK15‐EPO, being downregulated 136.5‐fold. SIL1 is a nucleotide exchange factor that has been linked with the release of BiP‐clients in the ER lumen after sequestration with BiP, as well as suppression of PERK (Lemarié et al. 2021). Fellow HSP family member, HSPA2 is a molecular chaperone implicated in a wide variety of cellular processes including folding and transport of nascent polypeptides and the activation of proteolysis of misfolded proteins (Radons 2016). It is upregulated in CHO‐K1 cells at 31°C. LMAN2 is downregulated in CHO‐DP12 at 31°C and is important in the transportation of glycoproteins of unprocessed N‐glycans from the Golgi to the ER, working with BiP in the ER (K. Yamamoto 2021).

The glycosylation mechanism is also well represented in the differentially expressed proteins. Glucosidase GlcII (GANAB), required for cleaving glucose residues from immature glycoproteins, is a crucial part of the protein folding process and shows decreased expression at the lower temperature in CHO‐K1 and CHO‐SK‐15‐EPO but no differential expression between temperatures was found at day 4 in CHO‐DP12. The lectin chaperone Calnexin and the luminal PDI it interacts with, ERP29, show a similar trend of decreased expression at 31°C compared to 37°C in CHO‐SK15‐EPO and CHO‐DP12 but are not differentially expressed in CHO‐K1. DDOST, which is part of the oligosaccharyl transferase (OST) complex involved in glycan transfer is downregulated in CHO‐K1 at 31°C (Ramírez et al. 2019). UGGT1 also works as a quality control checkpoint for protein folding by detecting minor folding defects in glycoproteins and re‐glucosylating N‐glycans allowing recognition by lectin‐like molecular chaperones CNX and CRT (Ito et al. 2015), and similarly shows downregulation at 31°C in the producer cell lines CHO‐DP12 and CHO‐SK15‐EPO. In addition to changes in the expression levels of GANAB and UGGT1, the other glycosyltransferases PRKCSH, RPN2, STT3a showed differential expression in all three cell lines and STT3a is downregulated at 31°C compared to 37°C in all three, whereas PRKCSH and RPN2 are downregulated at 31°C in CHO‐K1 and CHO‐SK15‐EPO but upregulated at 31°C in CHO‐DP12. Glycosyltransferases MOGS and STT3b, found only in CHO‐SK15‐EPO, and RPN1, found in CHO‐SK15‐EPO and CHO‐K1 are all downregulated at 31°C. The downregulation of proteins related to folding and glucosylation in response to a temperature downshift is in line with the results reported by Bedoya‐López et al. (2016), who found a downregulation of UGGT1, MOGS, SSR1 and MAN1 (Bedoya‐López et al. 2016). These results contrast with findings from other studies, who find that folding pathway proteins are upregulated, and this is used to explain the increase in cell productivity (Torres et al. 2021; Yee et al. 2009). These differing results may be due to different cell lines, media, and the lower temperature of 31°C compared to 32/33°C. While the proteins involved in folding are downregulated, biological processes related to RNA activity are upregulated at 31°C (RNA splicing, mRNA processing, regulation of RNA splicing, mRNA metabolic processing).

Several PDIs were found to be downregulated in at least one of the cell lines at 31°C including P4HB, PDIA3 and PDIA4. The PDI family are crucial factors in both protein folding and quality control in the ER (Okumura et al. 2015). ERO1a is needed for proper function of PDIs and is similarly downregulated in CHO‐SK15‐EPO cells. DNAJB10, which is downregulated at 31°C, is an ER disulphide reductase required for efficient folding of proteins in the ER by catalysing the removal of nonnative disulphide bonds formed during the folding of proteins (Cunnea et al. 2003). DNAJB11 is a co‐chaperone for HSPA5 and it is required for proper folding, trafficking or degradation of proteins (Yu et al. 2000). No DNAJ‐protein family members involved in protein folding were found to be differentially expressed on day 4 in CHO‐K1. The DNAJ‐protein family is a subset of heat shock proteins that are involved in the assembly and disassembly of macromolecular complexes (Stetler et al. 2010). The functional diversity of HSP70 proteins is reliant on the co‐chaperone functions of DNAJ proteins (Hageman and Kampinga 2009). DNAJC3 and DNAJC10, as well as stress response protein HYOU1 (Ozawa et al. 1999), are all downregulated in CHO‐DP12 cells cultured at 31°C. It appears that in response to the arrest of cells in the G1 phase, the CHO cell lines reduce protein processing through the ER, possibly focusing on a reduction of the processing of cell cycle and growth‐related proteins that are not needed in the G1 phase.

On day 7, the three cell lines studied showed similar trends to day 4, with protein transport, folding and glycosylation related proteins downregulated at 31°C. UGGT1, PRKCSH, MOGS, STT3b, PDIA3, PDIA4, DNAJC10, LMAN1, ERP29 and CANX are all down regulated at 31°C in all cell lines. DNAJC3 and LMAN2 are downregulated in CHO‐DP12. HSPA2 and HYOU1 are downregulated in CHO‐SK15‐EPO and CHO‐K1. GANAB, STT3α and SIL1 are all downregulated in both CHO‐SK15‐EPO and CHO‐DP12, while glycotransferases DDOST and RPN2 (Ramírez et al. 2019) all downregulated in both CHO‐K1 and CHO‐DP12, and RPN1 and DAD1 are downregulated in only CHO‐K1. The chaperones BIP and DNAJB11 are both downregulated at 31°C in CHO‐K1 and CHO‐DP12. HSP90AA1 aids in the proper folding of specific target proteins through specific ATPase activity (Forsythe et al. 2001), and it is upregulated at 31°C in CHO‐DP12. This suggests that these cell lines are processing less proteins overall at 31°C than at 37°C, possibly due to the cells needing less proteins for cell replication, as reflected by the reduced growth rate at 31°C.

SEC. 23α is the only transport protein differentially expressed in all three lines on day 7, downregulated in CHO‐K1 and CHO‐SK15‐EPO but upregulated in CHO‐DP12, with SEC. 24 C downregulated in both CHO‐K1 and CHO‐DP12, SEC. 61α1 downregulated in CHO‐SK15‐EPO and SEC. 31α downregulated in CHO‐K1. SEC. 12 (PREB) is part of the regulation of coat protein complex II/COPII in ER to Golgi vesicle‐mediated transport (Melville et al. 2020) and is downregulated at 31°C in CHO‐DP12. The transmembrane protein CKAP4, which facilitates microtubule anchoring to the ER (Vedrenne et al. 2005), is downregulated at 31°C in CHO‐DP12. TXNDC5 introduces disulfide bonds into unfolded substrates rapidly, and then allows for more precise PDI‐mediated disulfide bond formation (Okumura et al. 2015), and is downregulated in both CHO‐K1 and CHO‐SK15‐EPO. This data suggests that the stress of temperature downshift affects the glycosylation ability of CHO cells, even at day 7, 5 days after the shift. The downregulation of so many proteins involved in the proper folding of nascent polypeptides would result in a mass build‐up of misfolded proteins, triggering the ERAD and UPR. Due to the limitations of bottom‐up proteomics, it is not possible to confirm which proteins may be misfolded and if they relate to specific processes, functions or pathways. While ubiquitinated peptides may come from proteins targeted for degradation, it cannot be confirmed that these are from misfolded proteins, and not just proteins no longer useful to cell activity. It is also impossible to rule out the regulatory function of ubiquitination on these proteins, due to a lack of research on ubiquitination in CHO cells.

There are few proteins common to both producer cell lines only, and not in CHO‐K1, with more being found in only one producer cell line. This indicates that CHO‐SK15‐EPO and CHO‐DP12 may have developed different responses to the stress of recombinant protein production, impacting their response to mild hypothermic shock. On day 4, neither producer cell line show Sec. 23α as differentially expressed, it is only downregulated (1.6‐fold) at 31°C in CHO‐K1. On day 7 however, CHO‐SK15‐EPO shows a similar response as CHO‐K1, downregulating this protein at 31°C (1.6‐fold and 1.5‐fold, respectively). On day 7, CHO‐DP12 upregulates Sec. 23α (1.7‐fold). This protein is part of the coat protein complex II (COPII) which promotes the formation of transport vesicles from the ER, for moving proteins out of the ER (Paccaud et al. 1996). It is possible that CHO‐DP12 may use an altered COPII to transport the increased IgG1 antibody being produced by cells at 31°C. Other proteins involved in transport (Sec. 24, Sec. 12, CKAP4) are downregulated at 31°C in CHO‐DP12 but are not differentially expressed in CHO‐SK15‐EPO, suggesting that lower culture temperature does not illicit the same changes in transport pathways in these cells as in CHO‐DP12. SIL1 (136‐fold) is downregulated at 31°C in only CHO‐SK15‐EPO on day 4, and is still downregulated at day 7, but to a lesser extent (16‐fold). On day 7 it also downregulated in CHO‐DP12 at 31°C (1.7‐fold) but is not differentially expressed in CHO‐K1 at either time point, suggesting that the downregulation of this protein may play a role in the increased productivity of producer cells at 31°C. Given the role of SIL1 in the release of BiP‐clients, as well as suppression of PERK (Lemarié et al. 2021), there is an indication that producer cells, in particular CHO‐SK15‐EPO, may use this protein as a major part of their normal protein processing, and in response to a build‐up of misfolded proteins, it must be downregulated to allow for an appropriate ER stress response.

On day 4, there are 6 DE proteins shared between the three cell lines, all downregulated at 31°C (ZC3HAV1, USP5, PI4KA, ARL2, PSMD13, CCT7). There is 9 DE proteins shared between all three cell lines on day 7. Four of these proteins are upregulated at 31°C in all cell lines (GJA1, PSMD2, UBA1, HUWE1). Three are upregulated at 31°C in CHO‐K1 and CHO‐SK15‐EPO but downregulated at 31°C in CHO‐DP12 (GNAI3, MAP1B, PPP2R1A). RDX is downregulated at 31°C in CHO‐K1 and CHO‐DP12 but upregulated in CHO‐SK15‐EPO, and USP5 is upregulated at 31°C in CHO‐K1 and CHO‐DP12 but downregulated in CHO‐SK15‐EPO. USP5 is the only protein found in all cell lines differentially expressed at both day 4 and day 7. It is a ubiquitin‐specific protease, involved in removing ubiquitin from the proximal end of the polyubiquitin chains not conjugated to a target protein, therefore maintaining the free ubiquitin pool (Clague et al. 2019).

#### 3.5.2 ERAD

The ERAD pathway plays a crucial role in maintaining protein quality control, especially in CHO cells producing recombinant proteins. As these cells are engineered to express high levels of foreign proteins, the ER is put under extra pressure. ERAD recognises misfolded or improperly modified proteins in the ER and targets them for retro‐translocation into the cytosol, where they are ubiquitinated and degraded by the proteasome. This process helps prevent the accumulation of defective proteins, mitigating the risk of apoptosis in cells, as well as contributing to quality of recombinant proteins produced.

On day 4, both Endoplasmin (HSP90AB1) and PLAA are differentially expressed at 31°C in all cell lines, with Endoplasmin being downregulated in all, whereas PLAA is downregulated in CHO‐K1 and CHO‐SK15‐EPO but upregulated in CHO‐DP12. BCAP31 is a chaperone protein that is one of the most abundant ER proteins and is involved in the export of secreted proteins in the ER, the recognition of abnormally folded proteins and their targeting to the ERAD (Annaert et al. 1997). It is downregulated in CHO‐SK15‐EPO but upregulated in CHO‐K1 at 31°C. SSR4 is downregulated in CHO‐SK15‐EPO at 31°C.

Several proteins related to ubiquitin's role in the ERAD pathway are differentially expressed on day 4 in only CHO‐DP12 cells. NSFL1C, also known as p47, has a C‐terminal domain which includes a UBX domain (Yuan et al. 2001) and is known to interact with VCP (Uchiyama et al. 2003). UBXD2 is a membrane protein of the ER that binds VCP and is crucial in the promotion of ERAD (Lim et al. 2009). UBQLN2 is a vital regulator of different protein degradation pathways including the ubiquitin‐proteasome system (UPS), autophagy and the ERAD pathway. It allows for proteasomal targeting of misfolded proteins for degradation by binding to their polyubiquitin chains and by interacting with subunits in the proteasome (Kleijnen et al. 2000). NSFL1C, UBXD2 and UBQLN2 are all upregulated at 31°C. STUB1, an E3 ubiquitin‐protein ligase, is involved in targeting misfolded proteins towards proteasomal degradation (Ballinger et al. 1999), and is downregulated at 31°C in CHO‐DP12. This indicates that there may be a temporal aspect to ERAD activity, given that most of the DE proteins from that pathway are upregulated in CHO‐DP12, but STUB1 is downregulated, suggesting that not all proteins are required throughout the process. The presence of these proteins as differentially expressed in CHO‐DP12 only highlights the differences between CHO cells line in their biological processes and response to environmental factors. CHO‐SK15‐EPO is a producer cell line derived directly from CHO‐K1, which may a reason why CHO‐DP12 has a different ERAD response to these two cell lines.

On day 7, only Endoplasmin shows differential expression in all cell lines, being downregulated. Endoplasmin engages with a wide variety of client proteins, and is hence, involved in several different cell functions (Retzlaff et al. 2009), and can be linked to transport of cytosolic protein peptides through the ERGIC (endoplasmic reticulum‐Golgi intermediate compartment) (Zhang et al. 2020). In CHO‐SK15‐EPO, only one other protein involved in ERAD is differentially expressed, UBE2J1, which is upregulated at 31°C. UBE2J1 catalyses the covalent attachment of ubiquitin to other proteins and allows for the selective degradation of misfolded proteins from the ER and is essential for cells to recover from ER stress (Elangovan et al. 2017). This is suggestive of a much‐reduced ERAD response in CHO‐SK15‐EPO by day 7, again highlighting cell line differences in response to temperature downshift. On day 7, BCAP31 is only differentially expressed in CHO‐DP12, being downregulated at 31°C. HSPA8, which is upregulated at 31°C in CHO‐DP12, is a versatile molecular chaperone involved in many cellular processes, including proteolysis of misfolded proteins, assembly of protein complexes, and is critical in the protein quality control system (Grove et al. 2011; Y. Yamamoto et al. 2010).

In CHO‐K1 cells, PLAA, NSFL1C, and SKP1 were all upregulated at 31°C versus 37°C on day 7. PLAA is involved in protein ubiquitination, sorting and degradation through its association with VCP (Papadopoulos et al. 2017). SKP1 is one component of the SCF (SKP1‐CUL1‐F‐box protein) ubiquitin ligase complex, which mediates ubiquitination of proteins (Man et al. 2015). Another component of this complex, CUL1, is upregulated in CHO‐DP12 at 31°C. PLAA continues to be upregulated at 31°C in CHO‐DP12. UBE2D4 is a ligase that enables ubiquitin conjugating enzyme activity, allowing protein polyubiquitination (David et al. 2010) and is also upregulated at 31°C in CHO‐DP12. The upregulation of these proteins is suggestive of an increase in ubiquitination, likely due to misfolded protein build up. The shift from downregulation of PLAA on day 4 at 31°C in CHO‐K1, to upregulation at 31°C on day 7 supports this conclusion given its function to help transfer ubiquitinated proteins to the proteasome for degradation (Qiu et al. 2010). The increase in number of proteins being differentially expressed at 31°C on day 7 compared to day 4 also indicates that the number of proteins requiring degradation is greater at day 7. This is also seen in the number of differentially expressed ubiquitinated proteins seen on day 7, in particular in CHO‐K1.

The upregulation of two ERAD proteins at 31°C, UBXD2 (1.5‐fold) and UBQLN2 (1.7‐fold), on day 4 in CHO‐DP12, when neither protein is differentially expressed in CHO‐K1 or CHO‐SK15‐EPO could suggest that the mild hypothermic conditions cause a build‐up of misfolded proteins faster in CHO‐DP12 than the other cell lines, requiring a more pronounced ERAD response than the other cell lines. Evidence for this is shown by 8 ubiquitinated DE proteins in the proteasome KEGG pathway in CHO‐DP12 at day 4, and 17 DE proteins in that pathway form the whole cell data, more than in either CHO‐K1 or CHO‐SK15‐EPO, again indicating that the difference in cell line lineage may cause differences in the ERAD pathway. There are also 15 DE proteins from the whole cell proteome identified in the KEGG pathway “Ubiquitin mediated proteolysis” pathway (cge04120).

#### 3.5.3 UPR

The UPR is a key adaptive mechanism activated in response to the accumulation of misfolded or unfolded proteins in the ER. When recombinant proteins are overexpressed, the ER becomes stressed, triggering the UPR to restore protein homeostasis. The UPR works through three main signalling pathways—IRE1, PERK, and ATF6—that collectively reduce protein synthesis, enhance the expression of molecular chaperones, and promote protein degradation via ERAD (Stolz and Wolf 2010). This balance helps cells cope with the increased protein load, ensuring optimal folding conditions and preventing cell death during large‐scale production of recombinant proteins.

On day 4, PDIA6 is downregulated at 31°C in CHO‐K1 and CHO‐DP12. PDIA6 can limit UPR activity through inhibition of the crucial UPR activator, IRE1α, by converting oligomeric IRE1α to a monomeric form via a process of forming and breaking of disulfide bonds (Eletto et al. 2014). The mitochondrial apoptogenic protein, BAK1, has the ability to localise to the ER during times of ER stress, allowing a progressive depletion of ER Ca^2+^ and induction of caspase 12 cleavage and has been proven to induce apoptosis through depletion of Ca^2+^ allowing for the caspase cascade to cause cell death (Zong et al. 2003). It is upregulated in CHO‐SK15‐EPO at 31°C. EIF2S1 is upregulated at 31°C in CHO‐SK15‐EPO and CHO‐DP12 but downregulated in CHO‐K1. EIF2α is associated with UPR‐induced apoptosis, with studies linking EIF2α to caspase cleavage and possibly the caspase cascade (He et al. 2019; Nagata et al. 2007). Calpain 2 (CAPN2) is upregulated in CHO‐DP12 at 31°C. EIF2α's function as a transcription factor can be limited during periods of ER stress, by being phosphorylated by the kinase PERK, limiting general protein translation in the cell, with the phosphorylation of EIF2α also inducing the ATF4 mediated branch of the UPR (Cnop et al. 2017).

On day 7, PDIA6 continues to be downregulated in CHO‐K1 and CHO‐DP12 cells at 31°C. BAK1 shows a similar level of upregulation at 31°C in CHO‐SK15‐EPO as it did on day 4. EIF2S1 continues to be upregulated at 31°C in CHO‐DP12 but is not differentially expressed in CHO‐SK15‐EPO and CHO‐K1 at day 7. The transmembrane protein Wolframin (WFS1) is downregulated on both day 4 and day 7 in CHO‐SK15‐EPO at 31°C, but with a greater fold‐change at day 4 (8.38‐fold) than day 7 (1.77‐fold). It has previously been shown to be a UPR component that mitigates ER stress response in cells (Fonseca et al. 2005). CAPN2 is upregulated in CHO‐SK15‐EPO and CHO‐K1 at 31°C on day 7, while CAPN1 is downregulated in CHO‐DP12. Calpains are a family of Ca^2+^‐dependent intracellular cysteine proteases that cleave protein substrates, in a fashion that links them to a wide variety of biological functions including cell migration, cell cycle regulation, differentiation, and apoptosis (Goll et al. 2003). The upregulation of proteins such as BAK1 and CAPN2 and the downregulation of PDIA6 and WSF1 suggests that the UPR is engaging some apoptotic activity within both cell lines at 31°C on both day 4 and day 7 with no fold‐change observed in proteins involved in mitigating apoptosis. The KEGG pathway ‘Apoptosis’ is enriched in the whole cell proteome data in all three cell lines. Caspase‐dependent apoptosis seems to be the CHO cell response when the UPR has been unsuccessful in mediating the build‐up of misfolded proteins following temperature shift.

## 4 Conclusions

At 31°C, both producer cell lines exhibit enhanced specific productivity compared to 37°C. This increase in final titre is driven by prolonged culture viability under hypothermic conditions, where slower growth extends the production window and ultimately boosts final yield, with the increase in specific productivity likely driven by increased mRNA stability or enhanced recombinant gene transcription. The type of recombinant product being produced can affect the mechanisms of ER stress response, with the IgG producer and EPO producer having different expression of transport proteins at 31°C, including several secretory proteins (SEC. 12, SEC. 23, SEC. 24). The inclusion of the ubiquitinated proteome in this extensive profiling study provides new insights into the stress pathways of CHO cells, in response to an industrially relevant culture method. Enriching ubiquitinated peptides before mass spectrometry not only reveals proteins that are undetectable in whole‐cell lysates but also uncovers ubiquitination events on proteins previously identified without any indication of posttranslational modification. The prevalence of pathways related to protein binding, enzyme binding and organic cyclic compound binding in the ubiquitinated proteome indicates that these pathways are under tight cellular regulation during mild hypothermic stress. This is also seen in pathways related to cellular organisation on day 4, suggesting that proteins needed to redirect cell mechanisms during mild hypothermia are tightly regulated. Moreover, it may reflect the targeted degradation of proteins that have fulfilled their roles, indicating that by 48 h post‐temperature shift, cells may have already adapted and are actively clearing proteins no longer required. The differing expression of proteins between the whole cell proteome and ubiquitinated proteome shows that modified forms of proteins are differently regulated, and both forms of proteins play a role in the cellular response to hypothermic stress. The ubiquitinated proteome shows a more in‐depth view of the ER, and of the role of ubiquitin in the regulation of the cell's response to mild hypothermia. It has been found that proteins needed for correct ubiquitin regulation in the ER can themselves be subject to ubiquitination and given that this was seen in all three cell lines, this is likely a fundamental part of the CHO cell stress response. Furthermore, the data indicates that ubiquitination serves as a key regulatory strategy throughout different stages of cell culture, enabling precise control over protein function and turnover during adaptation.

This study underscores the importance of ubiquitin‐dependent regulation in cellular adaptation to hypothermia and the data generated is a valuable resource for investigating the molecular underpinnings of CHO cell responses to environmental stress, highlighting the need for posttranslational modification inclusive studies to truly understand proteome activity. These insights may guide the development of strategies to enhance CHO cell resilience and productivity in bioprocessing applications. The potential roles of proteins found in this study can be explored through cell line adaptation to under‐ or over‐expressing protein targets of interest. A genome‐wide CRISPR/Cas9 screening platform developed for CHO‐K1 cells identified 180 genes linked to resistance to hyperosmotic stress, and two novel target genes from that screen, *Zfr* and *Pnp*, were then knocked out in rCHO cells and a corresponding increased resistance to hyperosmotic stress was observed, improving mAb production (S. H. Kim et al. 2023). In three IgG producing CHO cell lines, the overexpression of Sar1A, which is needed for COPII vesicle formation, antibody production was increased by the modification of the secretion process (Tsunoda et al. 2024). These studies highlight the need for in‐depth profiling of CHO stress responses, to uncover novel targets for genetic engineering. ER stress marker proteins have also been used to develop reporter systems, for the detection of real‐time changes in the UPR. Both BiP and IRE1 were used to create fluorescent monitoring systems in producer CHO cell lines, producing a GSR mAb and IgG, respectively (Kyeong and Lee 2022; Roy et al. 2017). These systems could aid in engineering strategies and cell line selection for improved production of biotherapeutics, and our results allow for a greater insight into potential targets for further development of such reporter systems.

To build on the findings of this study, future research should include profiling the ubiquitinated proteome of other CHO cell lines and exploring the functional consequences of specific ubiquitination events and their potential for targeted modulation to optimise biomanufacturing outcomes.

## Author Contributions

David Ryan contributed to investigation, methodology, validation, and writing of the manuscript. Christiana‐Kondylo Sideri contributed to investigation and validation of CHO‐DP12 cell culture and productivity assay. Michael Henry contributed to the methodology and validation of the mass spectrometry data. Selvaprakash Karuppuchamy contributed to the methodology and validation of the ubiquitin enrichment and mass spectrometry analysis. Esen Efeoglu contributed to methodology and review of the manuscript. Paula Meleady contributed to conceptualisation, funding acquisition, project administration, supervision and review and editing of the manuscript. Authors have read and approved the submitted version.

## Conflicts of Interest

The authors declare no conflicts of interest.

## Supporting information

Supplementary Materials 1.

**Table 1:** KEGG pathways enriched in differentially expressed proteins in CHO‐K1, CHO‐SK15‐EPO and CHO‐DP12 on day 4 and day 7. **Table 2:** The KEGG pathways, molecular functions and processes enriched in **ubiquitinated differentially expressed proteins** in CHO‐K1, CHO‐SK15‐EPO and CHO‐DP12 on day 4 and day 7.

Supplementary Materials‐3.

## Data Availability

The mass spectrometry proteomics data has been submitted to and is available through the ProteomeXchange Consortium via the PRIDE (Perez‐Riverol et al. 2025) partner repository with the data set identifier PXD063085 and 10.6019/PXD063085.
