# Supplementary material for: Proteomic and Ubiquitinated Proteome Insights Into ER Stress Responses in Chinese Hamster Ovary Cells Under Mild Hypothermic Conditions
Source: Biotechnol Bioeng. 2025 Oct 28;123(1):5–25. doi: 10.1002/bit.70081 (PMC12699125; doi:10.1002/bit.70081)
Supplement: Supplementary file 2 — Table 1: KEGG pathways enriched in differentially expressed proteins in CHO‐K1, CHO‐SK15‐EPO and CHO‐DP12 on day 4 and day 7. Table 2: The KEGG pathways, molecular functions and processes enriched in ubiquitinated differentially expressed proteins in CHO‐K1, CHO‐SK15‐EPO and CHO‐DP12 on day 4 and day 7. [file BIT-123-5-s001.docx]

Supplementary Material

**Table 1:** KEGG pathways enriched in differentially expressed proteins in CHO-K1, CHO-SK15-EPO and CHO-DP12 on day 4 and day 7.

Day 4

|  |  | CHO-K1 | | | CHO-SK15-EPO | | | CHO-DP12 | | |
| --- | --- | --- | --- | --- | --- | --- | --- | --- | --- | --- |
| Term ID | Pathway | Count | Strength | FDR | Count | Strength | FDR | Count | Strength | FDR |
| cge01100 | Metabolic pathways | 129 | 0.48 | 8.60E-25 | 115 | 0.45 | 1.27E-19 | 122 | 0.37 | 3.78E-15 |
| cge01200 | Carbon metabolism | 37 | 0.98 | 1.23E-19 | 30 | 0.91 | 3.51E-14 | 26 | 0.74 | 2.23E-09 |
| cge05014 | Amyotrophic lateral sclerosis | 54 | 0.72 | 1.33E-18 | 21 | 0.32 | 0.0131 | 70 | 0.74 | 1.01E-24 |
| cge05012 | Parkinson disease | 42 | 0.79 | 1.12E-16 | - | - | - | 46 | 0.74 | 2.99E-16 |
| cge05020 | Prion disease | 38 | 0.71 | 6.11E-13 | - | - | - | 45 | 0.7 | 9.07E-15 |
| cge05010 | Alzheimer disease | 43 | 0.62 | 4.03E-12 | 20 | 0.31 | 0.0188 | 47 | 0.58 | 1.29E-11 |
| cge03040 | Spliceosome | 28 | 0.82 | 8.58E-12 | 21 | 0.71 | 5.38E-07 | 40 | 0.89 | 4.27E-18 |
| cge03013 | R- transport | 29 | 0.76 | 5.07E-11 | 22 | 0.66 | 1.42E-06 | 39 | 0.8 | 1.64E-15 |
| cge05016 | Huntington disease | 37 | 0.63 | 8.61E-11 | - | - | - | 48 | 0.66 | 1.38E-14 |
| cge03050 | Proteasome | 16 | 1.06 | 1.01E-09 | - | - | - | 17 | 1 | 2.23E-09 |
| cge05132 | Salmonella infection | 29 | 0.65 | 6.30E-09 | 18 | 0.47 | 0.0015 | 19 | 0.38 | 0.0056 |
| cge00020 | Citrate cycle (TCA cycle) | 13 | 1.11 | 2.15E-08 | 9 | 0.97 | 7.61E-05 | 13 | 1.02 | 2.02E-07 |
| cge01230 | Biosynthesis of amino acids | 18 | 0.86 | 2.84E-08 | 15 | 0.8 | 5.10E-06 | 10 | 0.51 | 0.0112 |
| cge04141 | Protein processing in endoplasmic reticulum | 24 | 0.7 | 2.84E-08 | 27 | 0.77 | 4.45E-10 | 27 | 0.66 | 2.12E-08 |
| cge00280 | Valine, leucine and isoleucine degradation | 14 | 0.95 | 1.78E-07 | 11 | 0.86 | 5.31E-05 | 10 | 0.72 | 0.00091 |
| cge00230 | Purine metabolism | 19 | 0.75 | 2.46E-07 | 10 | 0.49 | 0.0158 | 11 | 0.43 | 0.0215 |
| cge05100 | Bacterial invasion of epithelial cells | 16 | 0.85 | 2.46E-07 | 7 | 0.51 | 0.0419 | 12 | 0.63 | 0.00091 |
| cge00630 | Glyoxylate and dicarboxylate metabolism | 11 | 1.04 | 1.15E-06 | 9 | 0.97 | 7.61E-05 | 7 | 0.75 | 0.0044 |
| cge00970 | Aminoacyl-tR- biosynthesis | 12 | 0.95 | 1.65E-06 | 15 | 1.07 | 8.85E-09 | 9 | 0.74 | 0.0013 |
| cge04530 | Tight junction | 19 | 0.68 | 1.70E-06 | 13 | 0.54 | 0.0023 | 15 | 0.5 | 0.0021 |
| cge05203 | Viral carcinogenesis | 22 | 0.61 | 2.09E-06 | 19 | 0.57 | 7.86E-05 | 19 | 0.46 | 0.001 |
| cge03010 | Ribosome | 25 | 0.56 | 2.45E-06 | - | - | - | 38 | 0.66 | 1.81E-11 |
| cge05017 | Spinocerebellar ataxia | 18 | 0.66 | 6.44E-06 | - | - | - | 20 | 0.62 | 1.10E-05 |
| cge00030 | Pentose phosphate pathway | 10 | 0.98 | 9.22E-06 | 7 | 0.84 | 0.002 | 5 | 0.59 | 0.0478 |
| cge00010 | Glycolysis / Gluconeogenesis | 13 | 0.8 | 1.06E-05 | 10 | 0.71 | 0.0011 | 9 | 0.55 | 0.0112 |
| cge04810 | Regulation of actin cytoskeleton | 21 | 0.56 | 1.72E-05 | 17 | 0.49 | 0.0012 | 20 | 0.46 | 0.00091 |
| cge05205 | Proteoglycans in cancer | 20 | 0.58 | 1.72E-05 | 15 | 0.48 | 0.0031 | 15 | 0.37 | 0.0163 |
| cge00520 | Amino sugar and nucleotide sugar metabolism | 11 | 0.86 | 2.33E-05 | 7 | 0.68 | 0.0093 | 8 | 0.63 | 0.008 |
| cge03018 | R- degradation | 13 | 0.75 | 2.92E-05 | 8 | 0.56 | 0.017 | 8 | 0.45 | 0.043 |
| cge04210 | Apoptosis | 15 | 0.64 | 7.63E-05 | 11 | 0.52 | 0.0073 | 14 | 0.52 | 0.002 |
| cge00240 | Pyrimidine metabolism | 10 | 0.81 | 0.00013 | - | - | - | 7 | 0.57 | 0.0215 |
| cge03015 | mR- surveillance pathway | 13 | 0.68 | 0.00013 | - | - | - | 14 | 0.62 | 0.00034 |
| cge04612 | Antigen processing and presentation | 10 | 0.77 | 0.00024 | - | - | - | 8 | 0.59 | 0.0113 |
| cge00620 | Pyruvate metabolism | 8 | 0.9 | 0.00027 | 8 | 0.92 | 0.00037 | 9 | 0.86 | 0.00027 |
| cge00640 | Propanoate metabolism | 8 | 0.88 | 0.00032 | 11 | 1.04 | 2.59E-06 | 6 | 0.67 | 0.0162 |
| cge04114 | Oocyte meiosis | 13 | 0.63 | 0.00033 | - | - | - | 16 | 0.63 | 9.01E-05 |
| cge04670 | Leukocyte transendothelial migration | 13 | 0.62 | 0.00036 | - | - | - | 10 | 0.42 | 0.0319 |
| cge05135 | Yersinia infection | 14 | 0.59 | 0.00041 | - | - | - | - | - | - |
| cge00190 | Oxidative phosphorylation | 15 | 0.55 | 0.00047 | 10 | 0.4 | 0.0448 | 21 | 0.61 | 7.71E-06 |
| cge04066 | HIF-1 sig-ling pathway | 13 | 0.61 | 0.00047 | 10 | 0.51 | 0.0131 | - | - | - |
| cge05160 | Hepatitis C | 17 | 0.51 | 0.00047 | 12 | 0.38 | 0.033 | 16 | 0.4 | 0.0089 |
| cge05418 | Fluid shear stress and atherosclerosis | 14 | 0.57 | 0.00049 | 16 | 0.65 | 7.53E-05 | 17 | 0.57 | 0.00022 |
| cge00480 | Glutathione metabolism | 10 | 0.71 | 0.00054 | 11 | 0.77 | 0.00018 | 14 | 0.77 | 1.63E-05 |
| cge05170 | Human immunodeficiency virus 1 infection | 19 | 0.47 | 0.00057 | 16 | 0.41 | 0.0079 | 22 | 0.44 | 0.00067 |
| cge00072 | Synthesis and degradation of ketone bodies | 5 | 1.17 | 0.00068 | - | - | - | 4 | 0.99 | 0.0112 |
| cge01523 | Antifolate resistance | 7 | 0.89 | 0.00069 | - | - | - | - | - | - |
| cge00071 | Fatty acid degradation | 8 | 0.77 | 0.0011 | - | - | - | 9 | 0.74 | 0.0013 |
| cge00983 | Drug metabolism - other enzymes | 10 | 0.66 | 0.0011 | 8 | 0.59 | 0.0133 | 9 | 0.53 | 0.0132 |
| cge00650 | Butanoate metabolism | 6 | 0.93 | 0.0014 | - | - | - | - | - | - |
| cge04110 | Cell cycle | 13 | 0.55 | 0.0014 | - | - | - | 12 | 0.42 | 0.0163 |
| cge04962 | Vasopressin-regulated water reabsorption | 8 | 0.75 | 0.0014 | - | - | - | 7 | 0.61 | 0.0162 |
| cge00051 | Fructose and mannose metabolism | 7 | 0.81 | 0.0017 | 7 | 0.83 | 0.0021 | 8 | 0.78 | 0.0016 |
| cge04151 | PI3K-Akt sig-ling pathway | 23 | 0.37 | 0.0017 | 23 | 0.39 | 0.0019 | 21 | 0.25 | 0.0467 |
| cge00900 | Terpenoid backbone biosynthesis | 6 | 0.88 | 0.0021 | - | - | - | 5 | 0.72 | 0.022 |
| cge04144 | Endocytosis | 18 | 0.42 | 0.0022 | 20 | 0.48 | 0.00045 | 26 | 0.49 | 3.02E-05 |
| cge04152 | AMPK sig-ling pathway | 12 | 0.54 | 0.0022 | 15 | 0.66 | 8.64E-05 | - | - | - |
| cge04722 | Neurotrophin sig-ling pathway | 11 | 0.55 | 0.003 | - | - | - | - | - | - |
| cge01212 | Fatty acid metabolism | 8 | 0.67 | 0.0038 | - | - | - | - | - | - |
| cge04260 | Cardiac muscle contraction | 8 | 0.67 | 0.0038 | - | - | - | - | - | - |
| cge00100 | Steroid biosynthesis | 5 | 0.92 | 0.0042 | - | - | - | - | - | - |
| cge00670 | One carbon pool by folate | 5 | 0.92 | 0.0042 | - | - | - | - | - | - |
| cge01210 | 2-Oxocarboxylic acid metabolism | 5 | 0.92 | 0.0042 | 4 | 0.84 | 0.0249 | 5 | 0.83 | 0.0112 |
| cge00052 | Galactose metabolism | 6 | 0.79 | 0.0045 | 7 | 0.88 | 0.0015 | 5 | 0.62 | 0.0422 |
| cge05206 | MicroR-s in cancer | 13 | 0.47 | 0.0045 | 11 | 0.42 | 0.0268 | - | - | - |
| cge04932 | Non-alcoholic fatty liver disease | 13 | 0.47 | 0.0047 | - | - | - | 18 | 0.52 | 0.00038 |
| cge04520 | Adherens junction | 8 | 0.64 | 0.0048 | - | - | - | 7 | 0.5 | 0.0423 |
| cge03030 | D- replication | 6 | 0.77 | 0.0051 | - | - | - | 8 | 0.81 | 0.0012 |
| cge03320 | PPAR sig-ling pathway | 9 | 0.58 | 0.0053 | 9 | 0.6 | 0.007 | - | - | - |
| cge04510 | Focal adhesion | 14 | 0.44 | 0.0053 | 13 | 0.43 | 0.0136 | 16 | 0.41 | 0.0076 |
| cge05142 | Chagas disease | 10 | 0.54 | 0.0055 | - | - | - | - | - | - |
| cge04071 | Sphingolipid sig-ling pathway | 11 | 0.5 | 0.0057 | - | - | - | 12 | 0.46 | 0.0112 |
| cge05034 | Alcoholism | 12 | 0.48 | 0.0057 | - | - | - | 15 | 0.49 | 0.0024 |
| cge00270 | Cysteine and methionine metabolism | 7 | 0.67 | 0.0063 | - | - | - | 8 | 0.64 | 0.0076 |
| cge04015 | Rap1 sig-ling pathway | 14 | 0.41 | 0.008 | - | - | - | 14 | 0.33 | 0.0398 |
| cge00380 | Tryptophan metabolism | 6 | 0.71 | 0.0085 | - | - | - | - | - | - |
| cge04714 | Thermogenesis | 16 | 0.38 | 0.0085 | 18 | 0.45 | 0.002 | 26 | 0.5 | 2.21E-05 |
| cge05169 | Epstein-Barr virus infection | 15 | 0.39 | 0.0089 | - | - | - | 15 | 0.3 | 0.0439 |
| cge04261 | Adrenergic sig-ling in cardiomyocytes | 10 | 0.5 | 0.0096 | - | - | - | 11 | 0.45 | 0.0162 |
| cge00510 | N-Glycan biosynthesis | 7 | 0.62 | 0.0106 | 6 | 0.57 | 0.0387 | - | - | - |
| cge05134 | Legionellosis | 7 | 0.62 | 0.0106 | - | - | - | - | - | - |
| cge05133 | Pertussis | 7 | 0.61 | 0.0115 | - | - | - | - | - | - |
| cge04146 | Peroxisome | 8 | 0.55 | 0.0122 | 7 | 0.51 | 0.0393 | - | - | - |
| cge04062 | Chemokine sig-ling pathway | 13 | 0.4 | 0.0132 | - | - | - | - | - | - |
| cge04145 | Phagosome | 11 | 0.44 | 0.0134 | 13 | 0.53 | 0.0024 | 15 | 0.49 | 0.0023 |
| cge04730 | Long-term depression | 7 | 0.59 | 0.0136 | - | - | - | 8 | 0.56 | 0.0151 |
| cge05165 | Human papillomavirus infection | 19 | 0.31 | 0.0172 | 28 | 0.49 | 1.87E-05 | - | - | - |
| cge04360 | Axon guidance | 12 | 0.4 | 0.0195 | - | - | - | - | - | - |
| cge05211 | Re-l cell carcinoma | 7 | 0.56 | 0.0195 | - | - | - | 9 | 0.58 | 0.0084 |
| cge04915 | Estrogen sig-ling pathway | 10 | 0.44 | 0.0198 | - | - | - | 13 | 0.47 | 0.0076 |
| cge05161 | Hepatitis B | 12 | 0.39 | 0.0199 | - | - | - | - | - | - |
| cge05164 | Influenza A | 12 | 0.39 | 0.0199 | - | - | - | 15 | 0.4 | 0.0111 |
| cge05230 | Central carbon metabolism in cancer | 7 | 0.55 | 0.0205 | 10 | 0.72 | 0.00085 | - | - | - |
| cge05215 | Prostate cancer | 8 | 0.5 | 0.0214 | - | - | - | - | - | - |
| cge04918 | Thyroid hormone synthesis | 7 | 0.54 | 0.0219 | - | - | - | 8 | 0.51 | 0.0236 |
| cge04390 | Hippo sig-ling pathway | 11 | 0.4 | 0.022 | - | - | - | - | - | - |
| cge05145 | Toxoplasmosis | 9 | 0.46 | 0.0221 | - | - | - | - | - | - |
| cge04660 | T cell receptor sig-ling pathway | 8 | 0.49 | 0.0222 | - | - | - | - | - | - |
| cge04666 | Fc gamma R-mediated phagocytosis | 8 | 0.49 | 0.0238 | - | - | - | - | - | - |
| cge04922 | Glucagon sig-ling pathway | 8 | 0.47 | 0.0273 | 10 | 0.59 | 0.0048 | - | - | - |
| cge03008 | Ribosome biogenesis in eukaryotes | 7 | 0.51 | 0.0291 | 11 | 0.72 | 4.00E-04 | 8 | 0.48 | 0.0336 |
| cge04919 | Thyroid hormone sig-ling pathway | 9 | 0.43 | 0.0293 | 12 | 0.58 | 0.0021 | - | - | - |
| cge05200 | Pathways in cancer | 25 | 0.23 | 0.0302 | 28 | 0.3 | 0.0066 | 33 | 0.27 | 0.0076 |
| cge05163 | Human cytomegalovirus infection | 14 | 0.33 | 0.031 | 16 | 0.4 | 0.0091 | 16 | 0.3 | 0.0424 |
| cge04217 | Necroptosis | 11 | 0.37 | 0.033 | - | - | - | 14 | 0.39 | 0.0162 |
| cge05220 | Chronic myeloid leukemia | 7 | 0.49 | 0.0352 | 8 | 0.56 | 0.0161 | - | - | - |
| cge05235 | PD-L1 expression and PD-1 checkpoint pathway in cancer | 7 | 0.48 | 0.0376 | 7 | 0.5 | 0.0447 | - | - | - |
| cge05221 | Acute myeloid leukemia | 6 | 0.52 | 0.0397 | 7 | 0.61 | 0.017 | - | - | - |
| cge04540 | Gap junction | 7 | 0.47 | 0.0398 | - | - | - | 11 | 0.58 | 0.0029 |
| cge00500 | Starch and sucrose metabolism | 4 | 0.69 | 0.0405 | - | - | - | - | - | - |
| cge04621 | NOD-like receptor sig-ling pathway | 11 | 0.35 | 0.0405 | - | - | - | - | - | - |
| cge00513 | Various types of N-glycan biosynthesis | 5 | 0.57 | 0.0461 | - | - | - | - | - | - |
| cge04014 | Ras sig-ling pathway | 13 | 0.31 | 0.0463 | - | - | - | - | - | - |
| cge04137 | Mitophagy - animal | 6 | 0.5 | 0.0483 | - | - | - | - | - | - |
| cge04971 | Gastric acid secretion | 6 | 0.5 | 0.0483 | - | - | - | 7 | 0.48 | 0.047 |
| cge04142 | Lysosome | - | - | - | 16 | 0.65 | 7.61E-05 | 17 | 0.57 | 0.00023 |
| cge04910 | Insulin sig-ling pathway | - | - | - | 14 | 0.59 | 0.00069 | - | - | - |
| cge05225 | Hepatocellular carcinoma | - | - | - | 15 | 0.5 | 0.002 | - | - | - |
| cge04012 | ErbB sig-ling pathway | - | - | - | 9 | 0.61 | 0.0066 | - | - | - |
| cge01524 | Platinum drug resistance | - | - | - | 8 | 0.62 | 0.0091 | 10 | 0.62 | 0.0031 |
| cge05412 | Arrhythmogenic right ventricular cardiomyopathy | - | - | - | 7 | 0.65 | 0.0131 | - | - | - |
| cge00980 | Metabolism of xenobiotics by cytochrome P450 | - | - | - | 7 | 0.64 | 0.0135 | 10 | 0.69 | 0.0013 |
| cge00860 | Porphyrin and chlorophyll metabolism | - | - | - | 5 | 0.78 | 0.0158 | - | - | - |
| cge04218 | Cellular senescence | - | - | - | 11 | 0.44 | 0.0197 | - | - | - |
| cge05214 | Glioma | - | - | - | 7 | 0.59 | 0.0197 | - | - | - |
| cge05410 | Hypertrophic cardiomyopathy | - | - | - | 7 | 0.57 | 0.025 | - | - | - |
| cge04213 | Longevity regulating pathway - multiple species | - | - | - | 7 | 0.56 | 0.0268 | - | - | - |
| cge05222 | Small cell lung cancer | - | - | - | 8 | 0.51 | 0.0268 | - | - | - |
| cge04150 | mTOR sig-ling pathway | - | - | - | 11 | 0.41 | 0.0303 | - | - | - |
| cge04512 | ECM-receptor interaction | - | - | - | 7 | 0.54 | 0.033 | - | - | - |
| cge05414 | Dilated cardiomyopathy | - | - | - | 7 | 0.54 | 0.033 | - | - | - |
| cge04912 | GnRH sig-ling pathway | - | - | - | 7 | 0.53 | 0.0344 | 8 | 0.48 | 0.0336 |
| cge04120 | Ubiquitin mediated proteolysis | - | - | - | - | - | - | 15 | 0.5 | 0.002 |
| cge03430 | Mismatch repair | - | - | - | - | - | - | 6 | 0.86 | 0.0037 |
| cge04216 | Ferroptosis | - | - | - | - | - | - | 7 | 0.65 | 0.0112 |
| cge04961 | Endocrine and other factor-regulated calcium reabsorption | - | - | - | - | - | - | 7 | 0.64 | 0.0117 |
| cge00330 | Arginine and proline metabolism | - | - | - | - | - | - | 7 | 0.63 | 0.0132 |
| cge04723 | Retrograde endocan-binoid sig-ling | - | - | - | - | - | - | 13 | 0.39 | 0.0204 |
| cge05204 | Chemical carcinogenesis | - | - | - | - | - | - | 8 | 0.52 | 0.022 |
| cge04728 | Dopaminergic sy-pse | - | - | - | - | - | - | 11 | 0.42 | 0.0221 |
| cge00770 | Pantothe-te and CoA biosynthesis | - | - | - | - | - | - | 4 | 0.74 | 0.0411 |
| cge00982 | Drug metabolism - cytochrome P450 | - | - | - | - | - | - | 6 | 0.55 | 0.0422 |
| cge04914 | Progesterone-mediated oocyte maturation | - | - | - | - | - | - | 8 | 0.45 | 0.043 |
| cge04720 | Long-term potentiation | - | - | - | - | - | - | 6 | 0.54 | 0.0439 |
| cge04713 | Circadian entrainment | - | - | - | - | - | - | 8 | 0.44 | 0.0471 |
| cge04923 | Regulation of lipolysis in adipocytes | - | - | - | - | - | - | 6 | 0.53 | 0.0471 |

Day 7

|  |  | CHO-K1 | | | CHO-SK15-EPO | | | CHO-DP12 | | |
| --- | --- | --- | --- | --- | --- | --- | --- | --- | --- | --- |
| Term ID | Pathway | Count | Strength | FDR | Count | Strength | FDR | Count | Strength | FDR |
| cge01100 | Metabolic pathways | 198 | 0.52 | 1.17E-42 | 106 | 0.43 | 9.92E-17 | 164 | 0.56 | 9.71E-41 |
| cge05012 | Parkinson disease | 59 | 0.79 | 1.77E-22 | 15 | 0.38 | 0.0322 | 39 | 0.73 | 1.63E-13 |
| cge01200 | Carbon metabolism | 46 | 0.92 | 5.04E-22 | 18 | 0.7 | 1.03E-05 | 30 | 0.86 | 2.18E-13 |
| cge05014 | Amyotrophic lateral sclerosis | 67 | 0.66 | 5.50E-20 | 22 | 0.36 | 0.0104 | 51 | 0.66 | 2.58E-15 |
| cge05020 | Prion disease | 52 | 0.7 | 8.11E-17 | - | - | - | 34 | 0.63 | 8.02E-10 |
| cge05010 | Alzheimer disease | 58 | 0.61 | 3.28E-15 | 19 | 0.31 | 0.0434 | 38 | 0.54 | 1.15E-08 |
| cge03013 | R- transport | 38 | 0.73 | 4.04E-13 | 18 | 0.59 | 0.00018 | 26 | 0.68 | 1.76E-08 |
| cge05016 | Huntington disease | 49 | 0.61 | 5.94E-13 | - | - | - | 38 | 0.62 | 1.69E-10 |
| cge03050 | Proteasome | 21 | 1.03 | 8.78E-12 | 10 | 0.89 | 0.00018 | 15 | 1.01 | 1.81E-08 |
| cge04141 | Protein processing in endoplasmic reticulum | 33 | 0.69 | 1.25E-10 | 22 | 0.7 | 7.06E-07 | 33 | 0.81 | 1.79E-13 |
| cge01230 | Biosynthesis of amino acids | 24 | 0.83 | 3.59E-10 | 11 | 0.68 | 0.0016 | 14 | 0.72 | 3.08E-05 |
| cge04142 | Lysosome | 28 | 0.72 | 1.06E-09 | 12 | 0.54 | 0.0093 | 28 | 0.84 | 3.48E-12 |
| cge00280 | Valine, leucine and isoleucine degradation | 19 | 0.93 | 1.76E-09 | 7 | 0.68 | 0.0208 | 13 | 0.89 | 2.94E-06 |
| cge00020 | Citrate cycle (TCA cycle) | 16 | 1.05 | 2.50E-09 | 7 | 0.88 | 0.003 | 12 | 1.05 | 3.88E-07 |
| cge03040 | Spliceosome | 28 | 0.67 | 9.08E-09 | 21 | 0.73 | 7.06E-07 | 20 | 0.64 | 4.08E-06 |
| cge00190 | Oxidative phosphorylation | 27 | 0.66 | 2.47E-08 | - | - | - | 14 | 0.5 | 0.0025 |
| cge05017 | Spinocerebellar ataxia | 26 | 0.67 | 2.89E-08 | - | - | - | 19 | 0.66 | 5.07E-06 |
| cge04714 | Thermogenesis | 33 | 0.54 | 1.26E-07 | - | - | - | 21 | 0.47 | 0.00032 |
| cge00230 | Purine metabolism | 23 | 0.69 | 1.36E-07 | 10 | 0.51 | 0.0257 | 18 | 0.7 | 3.45E-06 |
| cge00640 | Propanoate metabolism | 14 | 0.98 | 1.37E-07 | 5 | 0.71 | 0.0434 | 9 | 0.91 | 9.59E-05 |
| cge00071 | Fatty acid degradation | 15 | 0.9 | 2.65E-07 | - | - | - | 11 | 0.88 | 1.75E-05 |
| cge00983 | Drug metabolism - other enzymes | 18 | 0.77 | 4.16E-07 | 7 | 0.54 | 0.0434 | 14 | 0.78 | 8.34E-06 |
| cge00010 | Glycolysis / Gluconeogenesis | 17 | 0.77 | 1.01E-06 | 9 | 0.68 | 0.0066 | 14 | 0.81 | 5.13E-06 |
| cge00520 | Amino sugar and nucleotide sugar metabolism | 14 | 0.81 | 5.09E-06 | 10 | 0.85 | 0.00022 | 12 | 0.87 | 9.22E-06 |
| cge05132 | Salmonella infection | 28 | 0.49 | 1.16E-05 | 16 | 0.43 | 0.0114 | 19 | 0.44 | 0.0012 |
| cge01212 | Fatty acid metabolism | 14 | 0.76 | 1.48E-05 | - | - | - | 13 | 0.85 | 5.13E-06 |
| cge00052 | Galactose metabolism | 11 | 0.9 | 1.50E-05 | - | - | - | 9 | 0.94 | 6.28E-05 |
| cge00620 | Pyruvate metabolism | 11 | 0.89 | 1.95E-05 | 5 | 0.73 | 0.0415 | 8 | 0.87 | 0.00044 |
| cge04932 | Non-alcoholic fatty liver disease | 22 | 0.55 | 2.10E-05 | - | - | - | 12 | 0.41 | 0.0192 |
| cge00970 | Aminoacyl-tR- biosynthesis | 12 | 0.8 | 3.41E-05 | 9 | 0.86 | 0.00049 | 7 | 0.69 | 0.0067 |
| cge05170 | Human immunodeficiency virus 1 infection | 26 | 0.45 | 9.01E-05 | 14 | 0.37 | 0.0434 | 16 | 0.36 | 0.0134 |
| cge00240 | Pyrimidine metabolism | 12 | 0.74 | 9.71E-05 | 6 | 0.63 | 0.0434 | 12 | 0.87 | 9.22E-06 |
| cge00630 | Glyoxylate and dicarboxylate metabolism | 10 | 0.85 | 9.73E-05 | - | - | - | 5 | 0.67 | 0.0282 |
| cge03030 | D- replication | 10 | 0.85 | 9.73E-05 | - | - | - | 7 | 0.81 | 0.0019 |
| cge01210 | 2-Oxocarboxylic acid metabolism | 8 | 0.98 | 0.00013 | - | - | - | 5 | 0.89 | 0.006 |
| cge00330 | Arginine and proline metabolism | 11 | 0.76 | 0.00014 | - | - | - | - | - | - |
| cge00480 | Glutathione metabolism | 13 | 0.68 | 0.00015 | 7 | 0.59 | 0.0385 | 14 | 0.83 | 3.57E-06 |
| cge00270 | Cysteine and methionine metabolism | 11 | 0.72 | 3.00E-04 | - | - | - | 10 | 0.8 | 0.00018 |
| cge00531 | Glycosaminoglycan degradation | 7 | 1.01 | 3.00E-04 | - | - | - | 6 | 1.06 | 0.00055 |
| cge00650 | Butanoate metabolism | 8 | 0.9 | 3.00E-04 | - | - | - | 8 | 1.02 | 6.42E-05 |
| cge05205 | Proteoglycans in cancer | 21 | 0.45 | 0.00046 | - | - | - | 20 | 0.55 | 4.57E-05 |
| cge04146 | Peroxisome | 13 | 0.61 | 0.00048 | 9 | 0.64 | 0.0102 | 9 | 0.58 | 0.0069 |
| cge00051 | Fructose and mannose metabolism | 9 | 0.77 | 0.00065 | 9 | 0.95 | 0.00018 | 8 | 0.84 | 0.00061 |
| cge04210 | Apoptosis | 16 | 0.52 | 0.00071 | 10 | 0.5 | 0.028 | 13 | 0.55 | 0.0015 |
| cge00380 | Tryptophan metabolism | 9 | 0.74 | 0.00095 | - | - | - | 7 | 0.75 | 0.0034 |
| cge00410 | beta-Alanine metabolism | 8 | 0.8 | 0.001 | - | - | - | 7 | 0.86 | 0.0012 |
| cge05100 | Bacterial invasion of epithelial cells | 12 | 0.57 | 0.0018 | 7 | 0.52 | 0.0482 | 8 | 0.52 | 0.0211 |
| cge04114 | Oocyte meiosis | 14 | 0.51 | 0.002 | - | - | - | - | - | - |
| cge04510 | Focal adhesion | 19 | 0.42 | 0.002 | - | - | - | 12 | 0.35 | 0.0436 |
| cge00030 | Pentose phosphate pathway | 8 | 0.73 | 0.0022 | 5 | 0.71 | 0.0434 | - | - | - |
| cge04723 | Retrograde endocan-binoid sig-ling | 17 | 0.44 | 0.0023 | - | - | - | - | - | - |
| cge00250 | Alanine, aspartate and glutamate metabolism | 8 | 0.72 | 0.0026 | 5 | 0.7 | 0.0434 | 6 | 0.72 | 0.01 |
| cge00072 | Synthesis and degradation of ketone bodies | 5 | 1.02 | 0.0027 | - | - | - | 5 | 1.14 | 0.0011 |
| cge04810 | Regulation of actin cytoskeleton | 20 | 0.39 | 0.0027 | 14 | 0.42 | 0.0242 | 20 | 0.52 | 0.00013 |
| cge05203 | Viral carcinogenesis | 19 | 0.4 | 0.003 | 13 | 0.42 | 0.028 | 16 | 0.45 | 0.0029 |
| cge00511 | Other glycan degradation | 6 | 0.85 | 0.0034 | - | - | - | 4 | 0.8 | 0.0267 |
| cge00670 | One carbon pool by folate | 6 | 0.85 | 0.0034 | - | - | - | 4 | 0.8 | 0.0267 |
| cge00860 | Porphyrin and chlorophyll metabolism | 7 | 0.76 | 0.0034 | 5 | 0.8 | 0.028 | 6 | 0.82 | 0.0044 |
| cge00310 | Lysine degradation | 9 | 0.62 | 0.0042 | - | - | - | - | - | - |
| cge04922 | Glucagon sig-ling pathway | 12 | 0.5 | 0.0053 | 8 | 0.51 | 0.0434 | - | - | - |
| cge04670 | Leukocyte transendothelial migration | 13 | 0.47 | 0.0054 | - | - | - | 10 | 0.48 | 0.0141 |
| cge00513 | Various types of N-glycan biosynthesis | 8 | 0.62 | 0.0072 | - | - | - | 7 | 0.69 | 0.0067 |
| cge00980 | Metabolism of xenobiotics by cytochrome P450 | 9 | 0.58 | 0.0072 | - | - | - | 8 | 0.65 | 0.0054 |
| cge04612 | Antigen processing and presentation | 9 | 0.58 | 0.0072 | 6 | 0.59 | 0.0458 | 9 | 0.7 | 0.0015 |
| cge05418 | Fluid shear stress and atherosclerosis | 14 | 0.42 | 0.0086 | - | - | - | 13 | 0.51 | 0.0028 |
| cge00900 | Terpenoid backbone biosynthesis | 6 | 0.73 | 0.0093 | - | - | - | 6 | 0.86 | 0.0031 |
| cge04962 | Vasopressin-regulated water reabsorption | 8 | 0.6 | 0.0093 | - | - | - | 7 | 0.67 | 0.0081 |
| cge05169 | Epstein-Barr virus infection | 19 | 0.34 | 0.0099 | - | - | - | 18 | 0.44 | 0.0016 |
| cge04145 | Phagosome | 14 | 0.4 | 0.0132 | - | - | - | 15 | 0.55 | 6.00E-04 |
| cge03420 | Nucleotide excision repair | 7 | 0.62 | 0.0139 | - | - | - | - | - | - |
| cge03018 | R- degradation | 10 | 0.49 | 0.0142 | - | - | - | - | - | - |
| cge05211 | Re-l cell carcinoma | 9 | 0.52 | 0.0147 | - | - | - | - | - | - |
| cge00100 | Steroid biosynthesis | 5 | 0.77 | 0.0148 | - | - | - | - | - | - |
| cge01040 | Biosynthesis of unsaturated fatty acids | 6 | 0.68 | 0.0148 | - | - | - | 7 | 0.86 | 0.0012 |
| cge03320 | PPAR sig-ling pathway | 10 | 0.48 | 0.0149 | - | - | - | 9 | 0.55 | 0.0088 |
| cge04066 | HIF-1 sig-ling pathway | 12 | 0.42 | 0.0157 | 9 | 0.48 | 0.0434 | 10 | 0.47 | 0.017 |
| cge04979 | Cholesterol metabolism | 7 | 0.58 | 0.0194 | - | - | - | - | - | - |
| cge00510 | N-Glycan biosynthesis | 8 | 0.53 | 0.0196 | 6 | 0.59 | 0.0458 | 9 | 0.7 | 0.0015 |
| cge04910 | Insulin sig-ling pathway | 13 | 0.39 | 0.0196 | - | - | - | 12 | 0.47 | 0.0072 |
| cge03430 | Mismatch repair | 5 | 0.72 | 0.0205 | - | - | - | - | - | - |
| cge00061 | Fatty acid biosynthesis | 4 | 0.84 | 0.0225 | - | - | - | - | - | - |
| cge04144 | Endocytosis | 19 | 0.29 | 0.0258 | 21 | 0.52 | 0.00021 | - | - | - |
| cge04666 | Fc gamma R-mediated phagocytosis | 10 | 0.43 | 0.0261 | - | - | - | 8 | 0.46 | 0.0383 |
| cge04015 | Rap1 sig-ling pathway | 16 | 0.32 | 0.0263 | - | - | - | - | - | - |
| cge04520 | Adherens junction | 8 | 0.49 | 0.0282 | - | - | - | - | - | - |
| cge04611 | Platelet activation | 11 | 0.4 | 0.0305 | - | - | - | 9 | 0.43 | 0.0364 |
| cge03015 | mR- surveillance pathway | 10 | 0.41 | 0.0319 | - | - | - | 8 | 0.44 | 0.0455 |
| cge04130 | S-RE interactions in vesicular transport | 6 | 0.58 | 0.0319 | 5 | 0.68 | 0.0434 | - | - | - |
| cge04971 | Gastric acid secretion | 8 | 0.47 | 0.0331 | - | - | - | - | - | - |
| cge04110 | Cell cycle | 12 | 0.36 | 0.0358 | - | - | - | 10 | 0.4 | 0.0363 |
| cge00500 | Starch and sucrose metabolism | 5 | 0.63 | 0.0367 | - | - | - | 5 | 0.76 | 0.0153 |
| cge00604 | Glycosphingolipid biosynthesis - ganglio series | 4 | 0.73 | 0.039 | - | - | - | - | - | - |
| cge01524 | Platinum drug resistance | 8 | 0.46 | 0.039 | - | - | - | 10 | 0.68 | 0.0012 |
| cge05204 | Chemical carcinogenesis | 8 | 0.46 | 0.039 | - | - | - | 8 | 0.58 | 0.011 |
| cge05230 | Central carbon metabolism in cancer | 8 | 0.46 | 0.039 | 7 | 0.58 | 0.041 | - | - | - |
| cge00603 | Glycosphingolipid biosynthesis - globo and isoglobo series | 4 | 0.7 | 0.0459 | - | - | - | 4 | 0.83 | 0.0229 |
| cge04120 | Ubiquitin mediated proteolysis | 12 | 0.34 | 0.0462 | - | - | - | - | - | - |
| cge04216 | Ferroptosis | 6 | 0.52 | 0.0472 | - | - | - | - | - | - |
| cge04530 | Tight junction | 12 | 0.34 | 0.0482 | - | - | - | 12 | 0.46 | 0.0093 |
| cge05134 | Legionellosis | 7 | 0.47 | 0.0482 | - | - | - | - | - | - |
| cge05206 | MicroR-s in cancer | - | - | - | 12 | 0.47 | 0.0224 | - | - | - |
| cge03008 | Ribosome biogenesis in eukaryotes | - | - | - | 8 | 0.6 | 0.0242 | 8 | 0.54 | 0.0167 |
| cge04912 | GnRH sig-ling pathway | - | - | - | 8 | 0.6 | 0.0242 | - | - | - |
| cge04919 | Thyroid hormone sig-ling pathway | - | - | - | 10 | 0.51 | 0.0248 | - | - | - |
| cge04918 | Thyroid hormone synthesis | - | - | - | 7 | 0.58 | 0.0422 | 8 | 0.57 | 0.0121 |
| cge00562 | Inositol phosphate metabolism | - | - | - | 7 | 0.57 | 0.0434 | - | - | - |
| cge00600 | Sphingolipid metabolism | - | - | - | 6 | 0.61 | 0.0434 | - | - | - |
| cge05034 | Alcoholism | - | - | - | 10 | 0.43 | 0.0434 | - | - | - |
| cge05216 | Thyroid cancer | - | - | - | 5 | 0.7 | 0.0434 | - | - | - |
| cge05412 | Arrhythmogenic right ventricular cardiomyopathy | - | - | - | 6 | 0.6 | 0.0434 | - | - | - |
| cge04152 | AMPK sig-ling pathway | - | - | - | 9 | 0.45 | 0.0457 | - | - | - |
| cge04540 | Gap junction | - | - | - | - | - | - | 10 | 0.6 | 0.0031 |
| cge04217 | Necroptosis | - | - | - | - | - | - | 14 | 0.45 | 0.0057 |
| cge00770 | Pantothe-te and CoA biosynthesis | - | - | - | - | - | - | 5 | 0.89 | 0.006 |
| cge04614 | Renin-angiotensin system | - | - | - | - | - | - | 5 | 0.87 | 0.0069 |
| cge00062 | Fatty acid elongation | - | - | - | - | - | - | 5 | 0.78 | 0.0134 |
| cge00982 | Drug metabolism - cytochrome P450 | - | - | - | - | - | - | 6 | 0.61 | 0.0244 |
| cge05200 | Pathways in cancer | - | - | - | - | - | - | 27 | 0.24 | 0.0258 |
| cge05416 | Viral myocarditis | - | - | - | - | - | - | 7 | 0.54 | 0.0267 |
| cge05322 | Systemic lupus erythematosus | - | - | - | - | - | - | 8 | 0.46 | 0.0364 |
| cge00040 | Pentose and glucuro-te interconversions | - | - | - | - | - | - | 4 | 0.72 | 0.0404 |
| cge04512 | ECM-receptor interaction | - | - | - | - | - | - | 7 | 0.49 | 0.0416 |

**Table 2:** The KEGG pathways, molecular functions and processes enriched in **ubiquitinated differentially expressed proteins** in CHO-K1, CHO-SK15-EPO and CHO-DP12 on day 4 and day 7.

**Day 4**

|  |  | CHO-SK15-EPO | | | CHO-DP12 | | |
| --- | --- | --- | --- | --- | --- | --- | --- |
| KEGG | Description | Count | Strength | FDR | Count | Strength | FDR |
| cge05012 | Parkinson disease | 7 | 0.87 | 0.02 | 9 | 1.01 | 4.51E-05 |
| cge04120 | Ubiquitin mediated proteolysis | 5 | 0.97 | 0.0271 | - | - | - |
| cge04130 | S-RE interactions in vesicular transport | 3 | 1.28 | 0.0271 | - | - | - |
| cge04145 | Phagosome | 5 | 0.96 | 0.0271 | - | - | - |
| cge04510 | Focal adhesion | 5 | 0.85 | 0.0271 | - | - | - |
| cge04520 | Adherens junction | 4 | 1.2 | 0.0271 | 3 | 1.1 | 0.0432 |
| cge04810 | Regulation of actin cytoskeleton | 6 | 0.88 | 0.0271 | 5 | 0.83 | 0.0246 |
| cge05014 | Amyotrophic lateral sclerosis | 7 | 0.69 | 0.0271 | 9 | 0.82 | 0.00046 |
| cge05100 | Bacterial invasion of epithelial cells | 4 | 1.1 | 0.0271 | - | - | - |
| cge05132 | Salmonella infection | 6 | 0.83 | 0.0271 | - | - | - |
| cge05135 | Yersinia infection | 5 | 1 | 0.0271 | - | - | - |
| cge05170 | Human immunodeficiency virus 1 infection | 6 | 0.82 | 0.0271 | - | - | - |
| cge03050 | Proteasome | 3 | 1.19 | 0.0295 | 8 | 1.65 | 1.44E-08 |
| cge05017 | Spinocerebellar ataxia | - | - | - | 8 | 1.2 | 1.30E-05 |
| cge03010 | Ribosome | - | - | - | 9 | 1 | 4.51E-05 |
| cge05203 | Viral carcinogenesis | - | - | - | 8 | 1.06 | 4.86E-05 |
| cge05016 | Huntington disease | - | - | - | 9 | 0.91 | 0.00014 |
| cge05020 | Prion disease | - | - | - | 8 | 0.92 | 0.00035 |
| cge05034 | Alcoholism | - | - | - | 6 | 1.06 | 0.00069 |
| cge05010 | Alzheimer disease | - | - | - | 8 | 0.78 | 0.0023 |
| cge05169 | Epstein-Barr virus infection | - | - | - | 6 | 0.88 | 0.0053 |
| cge05322 | Systemic lupus erythematosus | - | - | - | 4 | 1.08 | 0.0121 |
| cge05205 | Proteoglycans in cancer | - | - | - | 5 | 0.87 | 0.018 |
| cge04530 | Tight junction | - | - | - | 4 | 0.89 | 0.0432 |

|  |  | CHO-K1 | | | CHO-SK15-EPO | | | CHO-DP12 | | |
| --- | --- | --- | --- | --- | --- | --- | --- | --- | --- | --- |
| Term ID | Function | Count | Strength | FDR | Count | Strength | FDR | Count | Strength | FDR |
| GO:0005488 | Binding | 293 | 0.13 | 1.20E-11 | 111 | 0.19 | 2.28E-11 | 98 | 0.17 | 1.76E-06 |
| GO:0005515 | Protein binding | 183 | 0.17 | 2.64E-07 | 79 | 0.3 | 1.32E-09 | 67 | 0.25 | 2.50E-05 |
| GO:0000166 | Nucleotide binding | 75 | 0.28 | 7.37E-05 | 28 | 0.34 | 0.0303 | - | - | - |
| GO:0036094 | Small molecule binding | 81 | 0.26 | 7.37E-05 | - | - | - | - | - | - |
| GO:0043168 | Anion binding | 76 | 0.25 | 0.00033 | - | - | - | - | - | - |
| GO:0008092 | Cytoskeletal protein binding | 43 | 0.36 | 0.00037 | - | - | - | - | - | - |
| GO:0017076 | Purine nucleotide binding | 64 | 0.27 | 0.00052 | - | - | - | - | - | - |
| GO:0032553 | Ribonucleotide binding | 64 | 0.27 | 0.00052 | - | - | - | - | - | - |
| GO:0032555 | Purine ribonucleotide binding | 63 | 0.27 | 0.00057 | - | - | - | - | - | - |
| GO:0097159 | Organic cyclic compound binding | 140 | 0.15 | 0.00057 | 55 | 0.24 | 0.0051 | 52 | 0.24 | 0.0041 |
| GO:1901363 | Heterocyclic compound binding | 139 | 0.16 | 0.00057 | 54 | 0.23 | 0.0067 | 52 | 0.24 | 0.0035 |
| GO:0035639 | Purine ribonucleoside triphosphate binding | 61 | 0.27 | 0.00066 | 24 | 0.35 | 0.0478 | - | - | - |
| GO:0097367 | Carbohydrate derivative binding | 69 | 0.25 | 0.00066 | - | - | - | - | - | - |
| GO:0017111 | Nucleoside-triphosphatase activity | 28 | 0.42 | 0.0014 | - | - | - | 12 | 0.57 | 0.0333 |
| GO:0043167 | Ion binding | 129 | 0.15 | 0.0014 | - | - | - | - | - | - |
| GO:0019899 | Enzyme binding | 67 | 0.24 | 0.0018 | 34 | 0.43 | 6.93E-05 | 27 | 0.36 | 0.0167 |
| GO:0044877 | Protein-containing complex binding | 50 | 0.29 | 0.0018 | - | - | - | 23 | 0.46 | 0.0029 |
| GO:0003824 | Catalytic activity | 131 | 0.13 | 0.0086 | - | - | - | - | - | - |
| GO:0016787 | Hydrolase activity | 63 | 0.2 | 0.023 | - | - | - | - | - | - |
| GO:0016887 | ATP hydrolysis activity | 16 | 0.47 | 0.0269 | - | - | - | - | - | - |
| GO:0140657 | ATP-dependent activity | 24 | 0.36 | 0.0269 | - | - | - | - | - | - |
| GO:0008574 | Plus-end-directed microtubule motor activity | 4 | 1.14 | 0.0444 | - | - | - | - | - | - |
| GO:0030554 | Adenyl nucleotide binding | 47 | 0.23 | 0.0456 | - | - | - | - | - | - |
| GO:0003723 | R- binding | - | - | - | 21 | 0.42 | 0.0268 | 19 | 0.4 | 0.0433 |
| GO:0031625 | Ubiquitin protein ligase binding | - | - | - | 9 | 0.69 | 0.0432 | - | - | - |
| GO:0005198 | Structural molecule activity | - | - | - | - | - | - | 20 | 0.59 | 0.00025 |
| GO:0030527 | Structural constituent of chromatin | - | - | - | - | - | - | 5 | 1.11 | 0.0227 |
| GO:0016818 | Hydrolase activity, acting on acid anhydrides, in phosphorus-containing anhydrides | - | - | - | - | - | - | 13 | 0.56 | 0.0233 |
| GO:0019904 | Protein domain specific binding | - | - | - | - | - | - | 14 | 0.51 | 0.0333 |

|  |  | CHO-K1 | | | CHO-SK15-EPO | | | CHO-DP12 | | |
| --- | --- | --- | --- | --- | --- | --- | --- | --- | --- | --- |
| Term ID | Process | Count | Strength | FDR | Count | Strength | FDR | Count | Strength | FDR |
| GO:0009987 | Cellular process | 322 | 0.1 | 110 | 15994 | 0.12 | 0.00026 | 106 | 0.13 | 7.61E-06 |
| GO:0016043 | Cellular component organization | 132 | 0.17 | 49 | 5621 | 0.22 | 0.0279 | 48 | 0.24 | 0.007 |
| GO:0071840 | Cellular component organization or biogenesis | 137 | 0.16 | 52 | 5887 | 0.23 | 0.0146 | 51 | 0.25 | 0.0031 |
| GO:0006996 | Organelle organization | 92 | 0.2 | 39 | 3632 | 0.32 | 0.0098 | 41 | 0.37 | 0.00017 |
| GO:0007010 | Cytoskeleton organization | 44 | 0.32 | - | - | - | - | - | - | - |
| GO:0048522 | Positive regulation of cellular process | 131 | 0.15 | - | - | - | - | - | - | - |
| GO:0046907 | Intracellular transport | 43 | 0.32 | - | - | - | - | - | - | - |
| GO:0030162 | Regulation of proteolysis | 29 | 0.4 | - | - | - | - | - | - | - |
| GO:0048518 | Positive regulation of biological process | 140 | 0.14 | 56 | 6368 | 0.23 | 0.0113 | - | - | - |
| GO:0051179 | Localization | 106 | 0.17 | 44 | 4471 | 0.28 | 0.0113 | - | - | - |
| GO:0051649 | Establishment of localization in cell | 51 | 0.28 | - | - | - | - | - | - | - |
| GO:0051641 | Cellular localization | 70 | 0.22 | 33 | 2661 | 0.38 | 0.004 | - | - | - |
| GO:0006810 | Transport | 89 | 0.18 | 37 | 3660 | 0.29 | 0.0201 | - | - | - |
| GO:0051234 | Establishment of localization | 92 | 0.18 | - | - | - | - | - | - | - |
| GO:0031323 | Regulation of cellular metabolic process | 123 | 0.14 | - | - | - | - | - | - | - |
| GO:0008104 | Protein localization | - | - | 28 | 1924 | 0.45 | 0.0036 | - | - | - |
| GO:0033036 | Macromolecule localization | - | - | 31 | 2366 | 0.4 | 0.0037 | - | - | - |
| GO:0009607 | Response to biotic stimulus | - | - | 20 | 1261 | 0.48 | 0.0113 | - | - | - |
| GO:0016192 | Vesicle-mediated transport | - | - | 20 | 1231 | 0.5 | 0.0113 | - | - | - |
| GO:0023051 | Regulation of sig-ling | - | - | 36 | 3347 | 0.32 | 0.0113 | - | - | - |
| GO:0031349 | Positive regulation of defense response | - | - | 9 | 241 | 0.86 | 0.0113 | - | - | - |
| GO:0044419 | Biological process involved in interspecies interaction between organisms | - | - | 21 | 1351 | 0.48 | 0.0113 | - | - | - |
| GO:0048583 | Regulation of response to stimulus | - | - | 40 | 3843 | 0.3 | 0.0113 | - | - | - |
| GO:0010033 | Response to organic substance | - | - | 32 | 2827 | 0.34 | 0.0134 | 29 | 0.32 | 0.0211 |
| GO:0051707 | Response to other organism | - | - | 19 | 1213 | 0.48 | 0.0146 | - | - | - |
| GO:0071705 | Nitrogen compound transport | - | - | 21 | 1456 | 0.44 | 0.0146 | 20 | 0.45 | 0.009 |
| GO:0010646 | Regulation of cell communication | - | - | 35 | 3331 | 0.31 | 0.0171 | - | - | - |
| GO:0019538 | Protein metabolic process | - | - | 42 | 4367 | 0.27 | 0.0171 | 44 | 0.32 | 0.00056 |
| GO:0015031 | Protein transport | - | - | 17 | 1062 | 0.49 | 0.0225 | - | - | - |
| GO:1901564 | Organonitrogen compound metabolic process | - | - | 48 | 5394 | 0.23 | 0.0225 | 51 | 0.29 | 0.00034 |
| GO:0051246 | Regulation of protein metabolic process | - | - | 30 | 2708 | 0.33 | 0.024 | - | - | - |
| GO:0065007 | Biological regulation | - | - | 87 | 12586 | 0.12 | 0.0279 | - | - | - |
| GO:0050794 | Regulation of cellular process | - | - | 80 | 11253 | 0.14 | 0.0342 | - | - | - |
| GO:0006897 | Endocytosis | - | - | 10 | 422 | 0.66 | 0.0369 | - | - | - |
| GO:0006909 | Phagocytosis | - | - | 6 | 135 | 0.93 | 0.0382 | - | - | - |
| GO:0044085 | Cellular component biogenesis | - | - | 30 | 2815 | 0.31 | 0.0382 | 32 | 0.37 | 0.0023 |
| GO:0048523 | Negative regulation of cellular process | - | - | 43 | 4752 | 0.24 | 0.0382 | - | - | - |
| GO:0060255 | Regulation of macromolecule metabolic process | - | - | 51 | 6089 | 0.21 | 0.0402 | - | - | - |
| GO:0051050 | Positive regulation of transport | - | - | 16 | 1045 | 0.47 | 0.0422 | - | - | - |
| GO:0009605 | Response to exter-l stimulus | - | - | 26 | 2319 | 0.33 | 0.0484 | - | - | - |
| GO:0043170 | Macromolecule metabolic process | - | - | 52 | 6322 | 0.2 | 0.0484 | 53 | 0.24 | 0.004 |
| GO:0044260 | Cellular macromolecule metabolic process | - | - | - | - | - | - | 38 | 0.44 | 1.81E-05 |
| GO:0019941 | Modification-dependent protein catabolic process | - | - | - | - | - | - | 16 | 0.77 | 7.92E-05 |
| GO:0044237 | Cellular metabolic process | - | - | - | - | - | - | 62 | 0.26 | 0.00017 |
| GO:0006511 | Ubiquitin-dependent protein catabolic process | - | - | - | - | - | - | 15 | 0.75 | 0.00018 |
| GO:0030163 | Protein catabolic process | - | - | - | - | - | - | 17 | 0.67 | 2.00E-04 |
| GO:0044238 | Primary metabolic process | - | - | - | - | - | - | 65 | 0.24 | 2.00E-04 |
| GO:0071704 | Organic substance metabolic process | - | - | - | - | - | - | 66 | 0.22 | 0.00047 |
| GO:0008152 | Metabolic process | - | - | - | - | - | - | 68 | 0.21 | 0.00056 |
| GO:0006807 | Nitrogen compound metabolic process | - | - | - | - | - | - | 60 | 0.24 | 0.00069 |
| GO:0044249 | Cellular biosynthetic process | - | - | - | - | - | - | 30 | 0.4 | 0.0016 |
| GO:1901576 | Organic substance biosynthetic process | - | - | - | - | - | - | 30 | 0.38 | 0.0031 |
| GO:1903829 | Positive regulation of protein localization | - | - | - | - | - | - | 12 | 0.7 | 0.004 |
| GO:0022607 | Cellular component assembly | - | - | - | - | - | - | 29 | 0.38 | 0.0042 |
| GO:0032880 | Regulation of protein localization | - | - | - | - | - | - | 16 | 0.54 | 0.0064 |
| GO:0042176 | Regulation of protein catabolic process | - | - | - | - | - | - | 10 | 0.75 | 0.0064 |
| GO:0045862 | Positive regulation of proteolysis | - | - | - | - | - | - | 10 | 0.76 | 0.0064 |
| GO:0060341 | Regulation of cellular localization | - | - | - | - | - | - | 17 | 0.53 | 0.0064 |
| GO:0006508 | Proteolysis | - | - | - | - | - | - | 18 | 0.49 | 0.0079 |
| GO:0010498 | Proteasomal protein catabolic process | - | - | - | - | - | - | 10 | 0.73 | 0.0079 |
| GO:0051276 | Chromosome organization | - | - | - | - | - | - | 16 | 0.53 | 0.0079 |
| GO:0006334 | Nucleosome assembly | - | - | - | - | - | - | 6 | 1.04 | 0.009 |
| GO:0043434 | Response to peptide hormone | - | - | - | - | - | - | 10 | 0.72 | 0.0092 |
| GO:0045116 | Protein neddylation | - | - | - | - | - | - | 3 | 1.79 | 0.0101 |
| GO:0007049 | Cell cycle | - | - | - | - | - | - | 17 | 0.5 | 0.0105 |
| GO:1901698 | Response to nitrogen compound | - | - | - | - | - | - | 17 | 0.48 | 0.0143 |
| GO:0044248 | Cellular catabolic process | - | - | - | - | - | - | 20 | 0.42 | 0.02 |
| GO:0048285 | Organelle fission | - | - | - | - | - | - | 9 | 0.72 | 0.02 |
| GO:0051052 | Regulation of D- metabolic process | - | - | - | - | - | - | 11 | 0.62 | 0.0229 |
| GO:0009894 | Regulation of catabolic process | - | - | - | - | - | - | 15 | 0.5 | 0.0238 |
| GO:0009059 | Macromolecule biosynthetic process | - | - | - | - | - | - | 20 | 0.41 | 0.0292 |
| GO:0006338 | Chromatin remodeling | - | - | - | - | - | - | 8 | 0.74 | 0.034 |
| GO:0044271 | Cellular nitrogen compound biosynthetic process | - | - | - | - | - | - | 20 | 0.39 | 0.0402 |

Day 7

|  |  | CHO-K1 | | | | CHO-SK15-EPO | | | CHO-DP12 | | |
| --- | --- | --- | --- | --- | --- | --- | --- | --- | --- | --- | --- |
| KEGG | Description | Count | Strength | FDR | Count | | Strength | FDR | Count | Strength | FDR |
| cge03010 | Ribosome | 34 | 0.96 | 2.81E-18 | - | | - | - | - | - | - |
| cge04530 | Tight junction | 16 | 0.88 | 3.36E-07 | 5 | | 0.99 | 0.0328 | 6 | 1.44 | 3.44E-05 |
| cge05014 | Amyotrophic lateral sclerosis | 25 | 0.65 | 3.36E-07 | 6 | | 0.64 | 0.0498 | - | - | - |
| cge05017 | Spinocerebellar ataxia | 16 | 0.88 | 3.36E-07 | - | | - | - | - | - | - |
| cge03050 | Proteasome | 10 | 1.13 | 1.44E-06 | 3 | | 1.22 | 0.0328 | - | - | - |
| cge05203 | Viral carcinogenesis | 16 | 0.75 | 6.18E-06 | - | | - | - | - | - | - |
| cge05016 | Huntington disease | 19 | 0.61 | 2.80E-05 | - | | - | - | - | - | - |
| cge05012 | Parkinson disease | 16 | 0.64 | 9.61E-05 | 8 | | 0.95 | 0.0015 | 4 | 1.03 | 0.0193 |
| cge05132 | Salmonella infection | 15 | 0.64 | 0.00018 | - | | - | - | - | - | - |
| cge04151 | PI3K-Akt sig-ling pathway | 18 | 0.54 | 0.00037 | - | | - | - | - | - | - |
| cge05160 | Hepatitis C | 13 | 0.66 | 0.00037 | - | | - | - | - | - | - |
| cge04670 | Leukocyte transendothelial migration | 10 | 0.78 | 0.00042 | 4 | | 0.99 | 0.0328 | 4 | 1.37 | 0.0034 |
| cge04261 | Adrenergic sig-ling in cardiomyocytes | 10 | 0.77 | 0.00047 | 4 | | 0.98 | 0.0328 | - | - | - |
| cge04141 | Protein processing in endoplasmic reticulum | 12 | 0.67 | 0.00053 | - | | - | - | - | - | - |
| cge05020 | Prion disease | 15 | 0.58 | 0.00053 | - | | - | - | - | - | - |
| cge05165 | Human papillomavirus infection | 17 | 0.53 | 0.00055 | - | | - | - | - | - | - |
| cge03013 | R- transport | 12 | 0.65 | 0.00071 | - | | - | - | - | - | - |
| cge04144 | Endocytosis | 14 | 0.58 | 0.00073 | 6 | | 0.83 | 0.0328 | - | - | - |
| cge05212 | Pancreatic cancer | 8 | 0.84 | 0.00073 | - | | - | - | - | - | - |
| cge05142 | Chagas disease | 9 | 0.76 | 0.00083 | - | | - | - | - | - | - |
| cge04146 | Peroxisome | 8 | 0.82 | 0.00089 | 4 | | 1.13 | 0.0328 | - | - | - |
| cge05010 | Alzheimer disease | 17 | 0.49 | 0.0011 | 6 | | 0.65 | 0.0498 | - | - | - |
| cge04120 | Ubiquitin mediated proteolysis | 10 | 0.68 | 0.0013 | 5 | | 0.99 | 0.0328 | - | - | - |
| cge04520 | Adherens junction | 7 | 0.85 | 0.0015 | 3 | | 1.1 | 0.0498 | - | - | - |
| cge04728 | Dopaminergic sy-pse | 9 | 0.69 | 0.0022 | 4 | | 0.95 | 0.0328 | - | - | - |
| cge05230 | Central carbon metabolism in cancer | 7 | 0.82 | 0.0022 | - | | - | - | - | - | - |
| cge05200 | Pathways in cancer | 20 | 0.41 | 0.0026 | - | | - | - | - | - | - |
| cge05205 | Proteoglycans in cancer | 11 | 0.59 | 0.0026 | - | | - | - | 4 | 1.14 | 0.0124 |
| cge01523 | Antifolate resistance | 5 | 1.02 | 0.0027 | - | | - | - | - | - | - |
| cge05135 | Yersinia infection | 9 | 0.66 | 0.0028 | - | | - | - | 3 | 1.18 | 0.0314 |
| cge01100 | Metabolic pathways | 41 | 0.26 | 0.0031 | - | | - | - | - | - | - |
| cge04110 | Cell cycle | 9 | 0.66 | 0.0031 | - | | - | - | - | - | - |
| cge05163 | Human cytomegalovirus infection | 12 | 0.53 | 0.0039 | - | | - | - | - | - | - |
| cge03320 | PPAR sig-ling pathway | 7 | 0.74 | 0.0044 | 4 | | 1.11 | 0.0328 | - | - | - |
| cge04145 | Phagosome | 9 | 0.63 | 0.0044 | - | | - | - | 6 | 1.44 | 3.44E-05 |
| cge04540 | Gap junction | 7 | 0.74 | 0.0044 | - | | - | - | 3 | 1.37 | 0.0143 |
| cge05145 | Toxoplasmosis | 8 | 0.68 | 0.0044 | - | | - | - | - | - | - |
| cge04730 | Long-term depression | 6 | 0.79 | 0.0058 | - | | - | - | - | - | - |
| cge05169 | Epstein-Barr virus infection | 11 | 0.53 | 0.006 | - | | - | - | - | - | - |
| cge04071 | Sphingolipid sig-ling pathway | 8 | 0.64 | 0.0068 | - | | - | - | 3 | 1.2 | 0.0296 |
| cge04971 | Gastric acid secretion | 6 | 0.77 | 0.0071 | 3 | | 1.08 | 0.0498 | 3 | 1.46 | 0.0124 |
| cge04961 | Endocrine and other factor-regulated calcium reabsorption | 5 | 0.85 | 0.0086 | - | | - | - | - | - | - |
| cge04915 | Estrogen sig-ling pathway | 8 | 0.61 | 0.0087 | - | | - | - | - | - | - |
| cge05170 | Human immunodeficiency virus 1 infection | 11 | 0.5 | 0.0088 | - | | - | - | - | - | - |
| cge05418 | Fluid shear stress and atherosclerosis | 8 | 0.6 | 0.01 | - | | - | - | 3 | 1.16 | 0.0316 |
| cge04910 | Insulin sig-ling pathway | 8 | 0.6 | 0.0104 | - | | - | - | - | - | - |
| cge05414 | Dilated cardiomyopathy | 6 | 0.72 | 0.0106 | - | | - | - | - | - | - |
| cge00983 | Drug metabolism - other enzymes | 6 | 0.71 | 0.0112 | - | | - | - | - | - | - |
| cge04140 | Autophagy - animal | 8 | 0.59 | 0.0112 | - | | - | - | - | - | - |
| cge05161 | Hepatitis B | 9 | 0.54 | 0.0118 | - | | - | - | - | - | - |
| cge04114 | Oocyte meiosis | 7 | 0.63 | 0.0124 | - | | - | - | - | - | - |
| cge00240 | Pyrimidine metabolism | 5 | 0.78 | 0.0131 | - | | - | - | - | - | - |
| cge05100 | Bacterial invasion of epithelial cells | 6 | 0.69 | 0.0131 | - | | - | - | - | - | - |
| cge04611 | Platelet activation | 7 | 0.62 | 0.0133 | - | | - | - | - | - | - |
| cge00061 | Fatty acid biosynthesis | 3 | 1.13 | 0.0142 | - | | - | - | - | - | - |
| cge02010 | ABC transporters | 4 | 0.9 | 0.0142 | - | | - | - | - | - | - |
| cge04062 | Chemokine sig-ling pathway | 9 | 0.51 | 0.0152 | - | | - | - | - | - | - |
| cge00052 | Galactose metabolism | 4 | 0.88 | 0.0156 | - | | - | - | - | - | - |
| cge05412 | Arrhythmogenic right ventricular cardiomyopathy | 5 | 0.75 | 0.0156 | - | | - | - | - | - | - |
| cge04612 | Antigen processing and presentation | 5 | 0.74 | 0.0165 | - | | - | - | - | - | - |
| cge00230 | Purine metabolism | 7 | 0.59 | 0.0168 | - | | - | - | - | - | - |
| cge01212 | Fatty acid metabolism | 5 | 0.73 | 0.017 | - | | - | - | - | - | - |
| cge01230 | Biosynthesis of amino acids | 6 | 0.65 | 0.017 | 4 | | 1.09 | 0.0328 | - | - | - |
| cge04390 | Hippo sig-ling pathway | 8 | 0.54 | 0.017 | - | | - | - | - | - | - |
| cge05146 | Amoebiasis | 6 | 0.65 | 0.017 | - | | - | - | - | - | - |
| cge04015 | Rap1 sig-ling pathway | 9 | 0.49 | 0.0171 | - | | - | - | - | - | - |
| cge04152 | AMPK sig-ling pathway | 7 | 0.58 | 0.0171 | - | | - | - | - | - | - |
| cge05215 | Prostate cancer | 6 | 0.64 | 0.0171 | - | | - | - | - | - | - |
| cge04973 | Carbohydrate digestion and absorption | 4 | 0.84 | 0.0187 | - | | - | - | - | - | - |
| cge04371 | Apelin sig-ling pathway | 7 | 0.56 | 0.0205 | - | | - | - | - | - | - |
| cge04922 | Glucagon sig-ling pathway | 6 | 0.62 | 0.0206 | - | | - | - | - | - | - |
| cge03015 | mR- surveillance pathway | 6 | 0.61 | 0.0217 | - | | - | - | - | - | - |
| cge04810 | Regulation of actin cytoskeleton | 9 | 0.47 | 0.0226 | 5 | | 0.82 | 0.0328 | 4 | 1.11 | 0.0143 |
| cge03420 | Nucleotide excision repair | 4 | 0.79 | 0.0239 | - | | - | - | - | - | - |
| cge00100 | Steroid biosynthesis | 3 | 0.97 | 0.0251 | - | | - | - | - | - | - |
| cge01524 | Platinum drug resistance | 5 | 0.67 | 0.0251 | - | | - | - | - | - | - |
| cge05167 | Kaposi sarcoma-associated herpesvirus infection | 9 | 0.46 | 0.0251 | - | | - | - | - | - | - |
| cge05410 | Hypertrophic cardiomyopathy | 5 | 0.67 | 0.0251 | - | | - | - | - | - | - |
| cge04022 | cGMP-PKG sig-ling pathway | 7 | 0.53 | 0.0256 | - | | - | - | - | - | - |
| cge04920 | Adipocytokine sig-ling pathway | 5 | 0.66 | 0.0256 | - | | - | - | - | - | - |
| cge01200 | Carbon metabolism | 7 | 0.52 | 0.0265 | - | | - | - | - | - | - |
| cge04216 | Ferroptosis | 4 | 0.77 | 0.0265 | 3 | | 1.26 | 0.0328 | - | - | - |
| cge05034 | Alcoholism | 7 | 0.51 | 0.0299 | 4 | | 0.88 | 0.0498 | 4 | 1.26 | 0.0066 |
| cge04510 | Focal adhesion | 8 | 0.47 | 0.0311 | - | | - | - | - | - | - |
| cge00040 | Pentose and glucuro-te interconversions | 3 | 0.9 | 0.0346 | - | | - | - | - | - | - |
| cge05162 | Measles | 7 | 0.5 | 0.0346 | - | | - | - | - | - | - |
| cge04721 | Sy-ptic vesicle cycle | 5 | 0.62 | 0.0348 | - | | - | - | - | - | - |
| cge04962 | Vasopressin-regulated water reabsorption | 4 | 0.72 | 0.0348 | - | | - | - | - | - | - |
| cge04066 | HIF-1 sig-ling pathway | 6 | 0.54 | 0.0364 | - | | - | - | - | - | - |
| cge04923 | Regulation of lipolysis in adipocytes | 4 | 0.71 | 0.037 | - | | - | - | - | - | - |
| cge03040 | Spliceosome | 7 | 0.48 | 0.0372 | - | | - | - | - | - | - |
| cge03018 | R- degradation | 5 | 0.6 | 0.0373 | - | | - | - | - | - | - |
| cge00520 | Amino sugar and nucleotide sugar metabolism | 4 | 0.69 | 0.0416 | - | | - | - | - | - | - |
| cge04713 | Circadian entrainment | 5 | 0.59 | 0.0416 | - | | - | - | - | - | - |
| cge04924 | Renin secretion | 4 | 0.69 | 0.0416 | - | | - | - | - | - | - |
| cge04150 | mTOR sig-ling pathway | 7 | 0.46 | 0.0452 | - | | - | - | - | - | - |
| cge04935 | Growth hormone synthesis, secretion and action | 6 | 0.51 | 0.0468 | - | | - | - | - | - | - |
| cge04970 | Salivary secretion | - | - | - | 3 | | 1.08 | 0.0498 | - | - | - |
| cge05322 | Systemic lupus erythematosus | - | - | - | - | | - | - | 3 | 1.33 | 0.0161 |
| cge05206 | MicroR-s in cancer | - | - | - | - | | - | - | 3 | 1.1 | 0.0455 |

|  |  | CHO-K1 | | | CHO-SK15-EPO | | | CHO-DP12 | | |
| --- | --- | --- | --- | --- | --- | --- | --- | --- | --- | --- |
| Term ID | Function | Count | Strength | FDR | Count | Strength | FDR | Count | Strength | FDR |
| GO:0005488 | Binding | 400 | 0.16 | 1.62E-29 | 96 | 0.15 | 2.95E-05 | 44 | 0.19 | 0.003 |
| GO:0097159 | Organic cyclic compound binding | 225 | 0.26 | 1.22E-19 | - | - | - | - | - | - |
| GO:1901363 | Heterocyclic compound binding | 223 | 0.26 | 1.22E-19 | - | - | - | - | - | - |
| GO:0005515 | Protein binding | 254 | 0.22 | 1.56E-17 | 69 | 0.26 | 9.24E-06 | 30 | 0.28 | 0.0194 |
| GO:0036094 | Small molecule binding | 128 | 0.36 | 4.68E-16 | 29 | 0.33 | 0.0375 | - | - | - |
| GO:0043168 | Anion binding | 122 | 0.36 | 4.49E-15 | - | - | - | - | - | - |
| GO:0000166 | Nucleotide binding | 116 | 0.37 | 5.62E-15 | - | - | - | - | - | - |
| GO:0017076 | Purine nucleotide binding | 105 | 0.39 | 1.42E-14 | - | - | - | - | - | - |
| GO:0032555 | Purine ribonucleotide binding | 104 | 0.39 | 2.03E-14 | - | - | - | - | - | - |
| GO:0035639 | Purine ribonucleoside triphosphate binding | 100 | 0.38 | 9.42E-14 | - | - | - | - | - | - |
| GO:0097367 | Carbohydrate derivative binding | 109 | 0.35 | 8.81E-13 | - | - | - | - | - | - |
| GO:0044877 | Protein-containing complex binding | 83 | 0.4 | 3.72E-12 | - | - | - | 12 | 0.56 | 0.0357 |
| GO:0030554 | Adenyl nucleotide binding | 86 | 0.39 | 6.89E-12 | - | - | - | - | - | - |
| GO:0032559 | Adenyl ribonucleotide binding | 85 | 0.39 | 1.21E-11 | - | - | - | - | - | - |
| GO:0005198 | Structural molecule activity | 63 | 0.47 | 1.40E-11 | - | - | - | - | - | - |
| GO:0019899 | Enzyme binding | 104 | 0.33 | 4.66E-11 | 30 | 0.4 | 0.0011 | 16 | 0.51 | 0.014 |
| GO:0005524 | ATP binding | 81 | 0.38 | 6.73E-11 | - | - | - | - | - | - |
| GO:0003824 | Catalytic activity | 194 | 0.2 | 9.35E-11 | - | - | - | - | - | - |
| GO:0003723 | R- binding | 75 | 0.38 | 5.46E-10 | - | - | - | - | - | - |
| GO:0043167 | Ion binding | 182 | 0.2 | 8.01E-10 | - | - | - | - | - | - |
| GO:0016462 | Pyrophosphatase activity | 47 | 0.51 | 1.04E-09 | - | - | - | - | - | - |
| GO:0140657 | ATP-dependent activity | 43 | 0.52 | 5.36E-09 | - | - | - | - | - | - |
| GO:0003735 | Structural constituent of ribosome | 37 | 0.56 | 1.15E-08 | - | - | - | - | - | - |
| GO:0016787 | Hydrolase activity | 98 | 0.3 | 1.15E-08 | - | - | - | - | - | - |
| GO:0017111 | Nucleoside-triphosphatase activity | 43 | 0.5 | 1.15E-08 | - | - | - | - | - | - |
| GO:0140662 | ATP-dependent protein folding chaperone | 12 | 1.09 | 2.39E-07 | - | - | - | - | - | - |
| GO:0003676 | Nucleic acid binding | 126 | 0.23 | 2.52E-07 | - | - | - | - | - | - |
| GO:0016887 | ATP hydrolysis activity | 27 | 0.59 | 6.74E-07 | - | - | - | - | - | - |
| GO:0044183 | Protein folding chaperone | 14 | 0.92 | 6.94E-07 | - | - | - | - | - | - |
| GO:0042802 | Identical protein binding | 84 | 0.28 | 8.90E-07 | - | - | - | - | - | - |
| GO:0044389 | Ubiquitin-like protein ligase binding | 28 | 0.57 | 9.94E-07 | - | - | - | - | - | - |
| GO:0031625 | Ubiquitin protein ligase binding | 27 | 0.58 | 1.18E-06 | - | - | - | - | - | - |
| GO:0101005 | Deubiquitinase activity | 13 | 0.75 | 0.00014 | - | - | - | - | - | - |
| GO:0051082 | Unfolded protein binding | 13 | 0.71 | 0.00034 | - | - | - | - | - | - |
| GO:0019904 | Protein domain specific binding | 39 | 0.34 | 0.00061 | - | - | - | - | - | - |
| GO:0016874 | Ligase activity | 15 | 0.62 | 0.00062 | - | - | - | - | - | - |
| GO:0008289 | Lipid binding | 36 | 0.35 | 0.00075 | - | - | - | - | - | - |
| GO:0019843 | rR- binding | 13 | 0.66 | 0.00086 | - | - | - | - | - | - |
| GO:0032561 | Guanyl ribonucleotide binding | 25 | 0.43 | 0.001 | - | - | - | - | - | - |
| GO:0005525 | GTP binding | 24 | 0.44 | 0.0013 | - | - | - | - | - | - |
| GO:0042626 | ATPase-coupled transmembrane transporter activity | 11 | 0.7 | 0.0017 | - | - | - | - | - | - |
| GO:0005543 | Phospholipid binding | 26 | 0.4 | 0.002 | - | - | - | - | - | - |
| GO:0035615 | Clathrin adaptor activity | 5 | 1.22 | 0.0024 | - | - | - | - | - | - |
| GO:0140492 | Metal-dependent deubiquiti-se activity | 5 | 1.19 | 0.003 | - | - | - | - | - | - |
| GO:0008097 | 5S rR- binding | 7 | 0.9 | 0.0039 | - | - | - | - | - | - |
| GO:0003729 | mR- binding | 21 | 0.43 | 0.0041 | - | - | - | - | - | - |
| GO:0043130 | Ubiquitin binding | 10 | 0.69 | 0.0044 | 8 | 1.21 | 6.96E-05 | - | - | - |
| GO:0008022 | Protein C-terminus binding | 16 | 0.5 | 0.0054 | - | - | - | - | - | - |
| GO:0097157 | pre-mR- intronic binding | 5 | 1.12 | 0.0054 | - | - | - | - | - | - |
| GO:0032182 | Ubiquitin-like protein binding | 11 | 0.63 | 0.0062 | - | - | - | - | - | - |
| GO:0036002 | pre-mR- binding | 7 | 0.84 | 0.0071 | - | - | - | - | - | - |
| GO:0097718 | Disordered domain specific binding | 7 | 0.84 | 0.0071 | - | - | - | - | - | - |
| GO:0030620 | U2 snR- binding | 4 | 1.26 | 0.0094 | - | - | - | - | - | - |
| GO:0031072 | Heat shock protein binding | 11 | 0.58 | 0.0142 | - | - | - | - | - | - |
| GO:0019901 | Protein ki-se binding | 33 | 0.29 | 0.0177 | - | - | - | - | - | - |
| GO:0004843 | Cysteine-type deubiquiti-se activity | 9 | 0.64 | 0.0186 | - | - | - | - | - | - |
| GO:0140096 | Catalytic activity, acting on a protein | 73 | 0.18 | 0.0198 | - | - | - | - | - | - |
| GO:0034634 | Glutathione transmembrane transporter activity | 3 | 1.47 | 0.0201 | - | - | - | - | - | - |
| GO:0019900 | Ki-se binding | 35 | 0.27 | 0.0206 | - | - | - | - | - | - |
| GO:0017056 | Structural constituent of nuclear pore | 5 | 0.95 | 0.0212 | - | - | - | - | - | - |
| GO:0044548 | S100 protein binding | 4 | 1.09 | 0.0268 | - | - | - | - | - | - |
| GO:0036402 | Proteasome-activating activity | 3 | 1.4 | 0.0275 | - | - | - | - | - | - |
| GO:0030623 | U5 snR- binding | 3 | 1.33 | 0.0377 | - | - | - | - | - | - |
| GO:0035091 | Phosphatidylinositol binding | 16 | 0.4 | 0.0434 | - | - | - | - | - | - |
| GO:0016740 | Transferase activity | 71 | 0.16 | 0.0479 | - | - | - | - | - | - |
| GO:0140640 | Catalytic activity, acting on a nucleic acid | 25 | 0.3 | 0.0479 | - | - | - | - | - | - |
| GO:0004749 | Ribose phosphate diphosphoki-se activity | 3 | 1.27 | 0.0484 | - | - | - | - | - | - |
| GO:0019829 | ATPase-coupled cation transmembrane transporter activity | 6 | 0.74 | 0.0495 | - | - | - | - | - | - |
| GO:0033218 | Amide binding | 19 | 0.36 | 0.0495 | - | - | - | - | - | - |
| GO:0051117 | ATPase binding | - | - | - | - | - | - | 5 | 1.38 | 0.0044 |
| GO:0008525 | Phosphatidylcholine transporter activity | - | - | - | - | - | - | 3 | 1.96 | 0.0084 |
| GO:0005200 | Structural constituent of cytoskeleton | - | - | - | - | - | - | 4 | 1.29 | 0.0337 |
| GO:0031267 | Small GTPase binding | - | - | - | - | - | - | 6 | 0.95 | 0.0337 |

|  |  | CHO-K1 | | | CHO-SK15-EPO | | | CHO-DP12 | | |
| --- | --- | --- | --- | --- | --- | --- | --- | --- | --- | --- |
| Term ID | Process | Count | Strength | FDR | Count | Strength | FDR | Count | Strength | FDR |
| GO:0009987 | Cellular process | 437 | 0.13 | 4.84E-31 | 108 | 0.14 | 1.03E-06 | - | - | - |
| GO:0008152 | Metabolic process | 303 | 0.24 | 3.57E-29 | 65 | 0.19 | 0.011 | - | - | - |
| GO:0044238 | Primary metabolic process | 282 | 0.26 | 3.40E-28 | - | - | - | - | - | - |
| GO:0071704 | Organic substance metabolic process | 291 | 0.25 | 3.40E-28 | - | - | - | - | - | - |
| GO:0044237 | Cellular metabolic process | 262 | 0.27 | 2.30E-26 | 58 | 0.23 | 0.005 | - | - | - |
| GO:0006807 | Nitrogen compound metabolic process | 264 | 0.26 | 2.91E-26 | - | - | - | - | - | - |
| GO:1901564 | Organonitrogen compound metabolic process | 213 | 0.29 | 2.84E-22 | - | - | - | - | - | - |
| GO:0043170 | Macromolecule metabolic process | 234 | 0.27 | 1.05E-21 | - | - | - | - | - | - |
| GO:0044260 | Cellular macromolecule metabolic process | 140 | 0.39 | 9.72E-21 | - | - | - | - | - | - |
| GO:0019538 | Protein metabolic process | 179 | 0.31 | 4.02E-19 | - | - | - | - | - | - |
| GO:0034641 | Cellular nitrogen compound metabolic process | 164 | 0.33 | 5.76E-19 | - | - | - | - | - | - |
| GO:0071840 | Cellular component organization or biogenesis | 213 | 0.26 | 1.20E-17 | - | - | - | - | - | - |
| GO:0009058 | Biosynthetic process | 123 | 0.37 | 1.09E-15 | - | - | - | - | - | - |
| GO:0016043 | Cellular component organization | 200 | 0.25 | 2.90E-15 | - | - | - | - | - | - |
| GO:0044271 | Cellular nitrogen compound biosynthetic process | 92 | 0.44 | 2.90E-15 | - | - | - | - | - | - |
| GO:1901576 | Organic substance biosynthetic process | 120 | 0.36 | 3.55E-15 | - | - | - | - | - | - |
| GO:0060341 | Regulation of cellular localization | 70 | 0.52 | 5.05E-15 | - | - | - | 13 | 0.78 | 0.0017 |
| GO:0044249 | Cellular biosynthetic process | 115 | 0.36 | 2.39E-14 | - | - | - | - | - | - |
| GO:0032879 | Regulation of localization | 108 | 0.37 | 5.99E-14 | 29 | 0.41 | 0.0021 | 16 | 0.53 | 0.0067 |
| GO:0032880 | Regulation of protein localization | 64 | 0.53 | 6.59E-14 | - | - | - | 12 | 0.79 | 0.0022 |
| GO:0006996 | Organelle organization | 145 | 0.3 | 1.23E-13 | - | - | - | - | - | - |
| GO:0051179 | Localization | 165 | 0.26 | 4.58E-13 | 45 | 0.31 | 0.0017 | 22 | 0.38 | 0.0144 |
| GO:0051246 | Regulation of protein metabolic process | 118 | 0.34 | 5.42E-13 | - | - | - | - | - | - |
| GO:0030163 | Protein catabolic process | 54 | 0.56 | 1.30E-12 | - | - | - | - | - | - |
| GO:0043043 | Peptide biosynthetic process | 53 | 0.56 | 1.94E-12 | - | - | - | - | - | - |
| GO:0006412 | Translation | 52 | 0.57 | 2.00E-12 | - | - | - | - | - | - |
| GO:0051603 | Proteolysis involved in protein catabolic process | 49 | 0.58 | 3.26E-12 | 16 | 0.71 | 0.00072 | - | - | - |
| GO:0010033 | Response to organic substance | 119 | 0.32 | 3.63E-12 | - | - | - | - | - | - |
| GO:0051234 | Establishment of localization | 146 | 0.28 | 3.63E-12 | 42 | 0.35 | 0.00072 | 21 | 0.43 | 0.0071 |
| GO:0009056 | Catabolic process | 95 | 0.37 | 3.98E-12 | 25 | 0.41 | 0.011 | - | - | - |
| GO:0006518 | Peptide metabolic process | 57 | 0.52 | 4.58E-12 | - | - | - | - | - | - |
| GO:0043603 | Cellular amide metabolic process | 66 | 0.47 | 4.80E-12 | - | - | - | - | - | - |
| GO:0043604 | Amide biosynthetic process | 56 | 0.53 | 5.92E-12 | - | - | - | - | - | - |
| GO:0019941 | Modification-dependent protein catabolic process | 45 | 0.6 | 9.20E-12 | 14 | 0.71 | 0.0021 | - | - | - |
| GO:0033036 | Macromolecule localization | 104 | 0.34 | 1.77E-11 | - | - | - | 17 | 0.54 | 0.0037 |
| GO:0008104 | Protein localization | 91 | 0.37 | 1.81E-11 | 24 | 0.41 | 0.0147 | 16 | 0.61 | 0.0025 |
| GO:0009057 | Macromolecule catabolic process | 60 | 0.49 | 1.81E-11 | - | - | - | - | - | - |
| GO:1901360 | Organic cyclic compound metabolic process | 126 | 0.29 | 4.77E-11 | - | - | - | - | - | - |
| GO:1901566 | Organonitrogen compound biosynthetic process | 79 | 0.4 | 6.60E-11 | - | - | - | - | - | - |
| GO:0044265 | Cellular macromolecule catabolic process | 51 | 0.53 | 7.34E-11 | - | - | - | - | - | - |
| GO:0071705 | Nitrogen compound transport | 75 | 0.41 | 7.34E-11 | - | - | - | 13 | 0.64 | 0.0063 |
| GO:0006810 | Transport | 137 | 0.27 | 7.83E-11 | 39 | 0.34 | 0.0021 | 20 | 0.43 | 0.0106 |
| GO:1901575 | Organic substance catabolic process | 82 | 0.38 | 1.87E-10 | - | - | - | - | - | - |
| GO:0044248 | Cellular catabolic process | 77 | 0.39 | 2.19E-10 | 22 | 0.46 | 0.0074 | - | - | - |
| GO:0006511 | Ubiquitin-dependent protein catabolic process | 42 | 0.58 | 2.73E-10 | 13 | 0.68 | 0.005 | - | - | - |
| GO:0006139 | Nucleobase-containing compound metabolic process | 112 | 0.3 | 3.57E-10 | - | - | - | - | - | - |
| GO:0051128 | Regulation of cellular component organization | 104 | 0.32 | 3.84E-10 | - | - | - | 17 | 0.52 | 0.0063 |
| GO:0046907 | Intracellular transport | 68 | 0.42 | 4.44E-10 | - | - | - | - | - | - |
| GO:0006725 | Cellular aromatic compound metabolic process | 117 | 0.29 | 4.71E-10 | - | - | - | - | - | - |
| GO:0009894 | Regulation of catabolic process | 57 | 0.46 | 5.36E-10 | - | - | - | 10 | 0.7 | 0.0144 |
| GO:1901565 | Organonitrogen compound catabolic process | 60 | 0.45 | 5.36E-10 | - | - | - | - | - | - |
| GO:0048519 | Negative regulation of biological process | 175 | 0.21 | 5.44E-10 | - | - | - | - | - | - |
| GO:0010467 | Gene expression | 100 | 0.32 | 8.50E-10 | - | - | - | - | - | - |
| GO:0009059 | Macromolecule biosynthetic process | 77 | 0.38 | 1.01E-09 | - | - | - | - | - | - |
| GO:0045184 | Establishment of protein localization | 63 | 0.43 | 1.07E-09 | 17 | 0.47 | 0.0358 | 12 | 0.7 | 0.0044 |
| GO:0046483 | Heterocycle metabolic process | 114 | 0.29 | 1.40E-09 | - | - | - | - | - | - |
| GO:0015031 | Protein transport | 59 | 0.44 | 1.47E-09 | - | - | - | 11 | 0.7 | 0.0071 |
| GO:0006886 | Intracellular protein transport | 45 | 0.52 | 1.65E-09 | - | - | - | - | - | - |
| GO:0060255 | Regulation of macromolecule metabolic process | 191 | 0.19 | 1.79E-09 | - | - | - | - | - | - |
| GO:0051641 | Cellular localization | 106 | 0.3 | 2.06E-09 | - | - | - | 17 | 0.49 | 0.0092 |
| GO:0006508 | Proteolysis | 63 | 0.42 | 2.20E-09 | 17 | 0.46 | 0.0389 | - | - | - |
| GO:0044085 | Cellular component biogenesis | 110 | 0.29 | 2.26E-09 | 29 | 0.32 | 0.0382 | - | - | - |
| GO:0080090 | Regulation of primary metabolic process | 183 | 0.2 | 2.96E-09 | - | - | - | - | - | - |
| GO:0042176 | Regulation of protein catabolic process | 32 | 0.64 | 3.31E-09 | - | - | - | 8 | 1.03 | 0.0025 |
| GO:0034645 | Cellular macromolecule biosynthetic process | 59 | 0.43 | 3.38E-09 | - | - | - | - | - | - |
| GO:0019222 | Regulation of metabolic process | 202 | 0.18 | 3.54E-09 | - | - | - | - | - | - |
| GO:0071702 | Organic substance transport | 82 | 0.35 | 3.54E-09 | - | - | - | 16 | 0.63 | 0.0022 |
| GO:0071310 | Cellular response to organic substance | 88 | 0.33 | 6.62E-09 | - | - | - | - | - | - |
| GO:1903829 | Positive regulation of protein localization | 37 | 0.57 | 8.10E-09 | - | - | - | 7 | 0.84 | 0.0323 |
| GO:0048523 | Negative regulation of cellular process | 157 | 0.22 | 8.36E-09 | - | - | - | - | - | - |
| GO:0048518 | Positive regulation of biological process | 194 | 0.18 | 1.27E-08 | - | - | - | - | - | - |
| GO:0051726 | Regulation of cell cycle | 57 | 0.42 | 1.34E-08 | - | - | - | - | - | - |
| GO:0051247 | Positive regulation of protein metabolic process | 71 | 0.36 | 1.83E-08 | - | - | - | - | - | - |
| GO:0051171 | Regulation of nitrogen compound metabolic process | 175 | 0.19 | 2.70E-08 | - | - | - | - | - | - |
| GO:0070887 | Cellular response to chemical stimulus | 102 | 0.28 | 2.79E-08 | - | - | - | - | - | - |
| GO:0051649 | Establishment of localization in cell | 74 | 0.34 | 8.24E-08 | - | - | - | - | - | - |
| GO:0010498 | Proteasomal protein catabolic process | 30 | 0.6 | 1.32E-07 | 10 | 0.73 | 0.0147 | - | - | - |
| GO:0010638 | Positive regulation of organelle organization | 37 | 0.52 | 1.35E-07 | - | - | - | - | - | - |
| GO:0007049 | Cell cycle | 56 | 0.4 | 1.53E-07 | - | - | - | - | - | - |
| GO:0065007 | Biological regulation | 319 | 0.1 | 1.60E-07 | - | - | - | - | - | - |
| GO:0022607 | Cellular component assembly | 96 | 0.28 | 1.74E-07 | 27 | 0.34 | 0.038 | - | - | - |
| GO:0051049 | Regulation of transport | 79 | 0.31 | 2.12E-07 | 24 | 0.41 | 0.0134 | 13 | 0.52 | 0.0389 |
| GO:0048522 | Positive regulation of cellular process | 175 | 0.18 | 2.36E-07 | - | - | - | - | - | - |
| GO:0050789 | Regulation of biological process | 304 | 0.11 | 2.49E-07 | - | - | - | - | - | - |
| GO:1901362 | Organic cyclic compound biosynthetic process | 50 | 0.41 | 3.28E-07 | - | - | - | - | - | - |
| GO:0061077 | Chaperone-mediated protein folding | 14 | 0.95 | 4.92E-07 | - | - | - | - | - | - |
| GO:1904951 | Positive regulation of establishment of protein localization | 27 | 0.6 | 5.50E-07 | - | - | - | - | - | - |
| GO:0070201 | Regulation of establishment of protein localization | 36 | 0.5 | 7.13E-07 | - | - | - | - | - | - |
| GO:0009893 | Positive regulation of metabolic process | 130 | 0.22 | 7.41E-07 | - | - | - | - | - | - |
| GO:0051130 | Positive regulation of cellular component organization | 57 | 0.37 | 8.32E-07 | - | - | - | - | - | - |
| GO:1901698 | Response to nitrogen compound | 55 | 0.38 | 8.74E-07 | - | - | - | - | - | - |
| GO:0031647 | Regulation of protein stability | 29 | 0.56 | 9.34E-07 | - | - | - | - | - | - |
| GO:0010604 | Positive regulation of macromolecule metabolic process | 121 | 0.22 | 9.49E-07 | - | - | - | - | - | - |
| GO:0031323 | Regulation of cellular metabolic process | 167 | 0.17 | 2.32E-06 | - | - | - | - | - | - |
| GO:1904814 | Regulation of protein localization to chromosome, telomeric region | 8 | 1.34 | 3.20E-06 | - | - | - | - | - | - |
| GO:0016192 | Vesicle-mediated transport | 56 | 0.35 | 3.22E-06 | - | - | - | 11 | 0.64 | 0.0168 |
| GO:0055086 | Nucleobase-containing small molecule metabolic process | 35 | 0.48 | 3.39E-06 | - | - | - | - | - | - |
| GO:0065008 | Regulation of biological quality | 125 | 0.21 | 4.10E-06 | - | - | - | - | - | - |
| GO:0033043 | Regulation of organelle organization | 55 | 0.35 | 4.81E-06 | - | - | - | - | - | - |
| GO:0032273 | Positive regulation of protein polymerization | 14 | 0.86 | 5.01E-06 | - | - | - | - | - | - |
| GO:0050794 | Regulation of cellular process | 286 | 0.1 | 5.01E-06 | - | - | - | - | - | - |
| GO:1903828 | Negative regulation of protein localization | 20 | 0.67 | 5.01E-06 | - | - | - | - | - | - |
| GO:0006950 | Response to stress | 108 | 0.23 | 5.47E-06 | - | - | - | - | - | - |
| GO:0009117 | Nucleotide metabolic process | 32 | 0.49 | 6.56E-06 | - | - | - | - | - | - |
| GO:0070647 | Protein modification by small protein conjugation or removal | 43 | 0.4 | 9.50E-06 | - | - | - | - | - | - |
| GO:0051173 | Positive regulation of nitrogen compound metabolic process | 107 | 0.22 | 1.00E-05 | - | - | - | - | - | - |
| GO:0042221 | Response to chemical | 138 | 0.19 | 1.14E-05 | - | - | - | - | - | - |
| GO:2001252 | Positive regulation of chromosome organization | 15 | 0.78 | 1.14E-05 | - | - | - | - | - | - |
| GO:0036211 | Protein modification process | 97 | 0.24 | 1.18E-05 | - | - | - | - | - | - |
| GO:1901998 | Toxin transport | 10 | 1.04 | 1.19E-05 | - | - | - | - | - | - |
| GO:0033044 | Regulation of chromosome organization | 21 | 0.62 | 1.21E-05 | - | - | - | - | - | - |
| GO:0022402 | Cell cycle process | 42 | 0.4 | 1.23E-05 | - | - | - | - | - | - |
| GO:0010243 | Response to organonitrogen compound | 49 | 0.36 | 1.24E-05 | - | - | - | - | - | - |
| GO:0043933 | Protein-containing complex organization | 62 | 0.31 | 1.42E-05 | - | - | - | - | - | - |
| GO:0031329 | Regulation of cellular catabolic process | 39 | 0.42 | 1.45E-05 | - | - | - | - | - | - |
| GO:0045732 | Positive regulation of protein catabolic process | 18 | 0.68 | 1.45E-05 | - | - | - | 5 | 1.12 | 0.0215 |
| GO:0061024 | Membrane organization | 37 | 0.43 | 1.45E-05 | 14 | 0.62 | 0.0077 | - | - | - |
| GO:0019693 | Ribose phosphate metabolic process | 29 | 0.5 | 1.49E-05 | - | - | - | - | - | - |
| GO:0061136 | Regulation of proteasomal protein catabolic process | 18 | 0.68 | 1.51E-05 | - | - | - | - | - | - |
| GO:1904851 | Positive regulation of establishment of protein localization to telomere | 7 | 1.37 | 1.51E-05 | - | - | - | - | - | - |
| GO:0023051 | Regulation of sig-ling | 110 | 0.21 | 1.56E-05 | - | - | - | - | - | - |
| GO:0043412 | Macromolecule modification | 101 | 0.22 | 1.74E-05 | - | - | - | - | - | - |
| GO:1903050 | Regulation of proteolysis involved in protein catabolic process | 20 | 0.63 | 1.79E-05 | - | - | - | - | - | - |
| GO:0051640 | Organelle localization | 32 | 0.46 | 1.89E-05 | - | - | - | - | - | - |
| GO:0051050 | Positive regulation of transport | 48 | 0.36 | 2.02E-05 | - | - | - | - | - | - |
| GO:0010646 | Regulation of cell communication | 109 | 0.21 | 2.12E-05 | - | - | - | - | - | - |
| GO:0043161 | Proteasome-mediated ubiquitin-dependent protein catabolic process | 24 | 0.55 | 2.12E-05 | - | - | - | - | - | - |
| GO:0031334 | Positive regulation of protein-containing complex assembly | 18 | 0.67 | 2.14E-05 | - | - | - | 5 | 1.1 | 0.0245 |
| GO:0033554 | Cellular response to stress | 64 | 0.3 | 2.50E-05 | - | - | - | - | - | - |
| GO:0010564 | Regulation of cell cycle process | 37 | 0.42 | 2.65E-05 | - | - | - | - | - | - |
| GO:0006457 | Protein folding | 20 | 0.61 | 2.91E-05 | - | - | - | - | - | - |
| GO:1905475 | Regulation of protein localization to membrane | 18 | 0.66 | 2.94E-05 | - | - | - | - | - | - |
| GO:1902531 | Regulation of intracellular sig-l transduction | 66 | 0.29 | 2.97E-05 | - | - | - | - | - | - |
| GO:0065009 | Regulation of molecular function | 99 | 0.22 | 3.03E-05 | - | - | - | - | - | - |
| GO:0010605 | Negative regulation of macromolecule metabolic process | 92 | 0.23 | 3.18E-05 | - | - | - | - | - | - |
| GO:0050821 | Protein stabilization | 21 | 0.59 | 3.60E-05 | - | - | - | - | - | - |
| GO:0032388 | Positive regulation of intracellular transport | 18 | 0.65 | 3.73E-05 | - | - | - | - | - | - |
| GO:1904375 | Regulation of protein localization to cell periphery | 15 | 0.73 | 4.06E-05 | - | - | - | - | - | - |
| GO:0090304 | Nucleic acid metabolic process | 80 | 0.25 | 4.61E-05 | - | - | - | - | - | - |
| GO:0009165 | Nucleotide biosynthetic process | 19 | 0.62 | 4.81E-05 | - | - | - | - | - | - |
| GO:0009892 | Negative regulation of metabolic process | 97 | 0.22 | 4.91E-05 | - | - | - | - | - | - |
| GO:0010629 | Negative regulation of gene expression | 42 | 0.37 | 5.39E-05 | - | - | - | - | - | - |
| GO:0034654 | Nucleobase-containing compound biosynthetic process | 38 | 0.39 | 5.81E-05 | - | - | - | - | - | - |
| GO:0033365 | Protein localization to organelle | 37 | 0.4 | 6.79E-05 | - | - | - | - | - | - |
| GO:0045862 | Positive regulation of proteolysis | 24 | 0.52 | 6.84E-05 | - | - | - | - | - | - |
| GO:0018130 | Heterocycle biosynthetic process | 40 | 0.38 | 7.08E-05 | - | - | - | - | - | - |
| GO:1903076 | Regulation of protein localization to plasma membrane | 13 | 0.77 | 7.38E-05 | - | - | - | - | - | - |
| GO:0065003 | Protein-containing complex assembly | 54 | 0.31 | 7.69E-05 | - | - | - | - | - | - |
| GO:0009719 | Response to endogenous stimulus | 57 | 0.3 | 8.84E-05 | - | - | - | - | - | - |
| GO:0072594 | Establishment of protein localization to organelle | 23 | 0.52 | 1.00E-04 | - | - | - | - | - | - |
| GO:0044281 | Small molecule metabolic process | 64 | 0.27 | 0.00011 | - | - | - | - | - | - |
| GO:0051052 | Regulation of D- metabolic process | 30 | 0.44 | 0.00011 | - | - | - | 7 | 0.8 | 0.0496 |
| GO:0006163 | Purine nucleotide metabolic process | 26 | 0.47 | 0.00015 | - | - | - | - | - | - |
| GO:0034341 | Response to interferon-gamma | 14 | 0.71 | 0.00015 | - | - | - | - | - | - |
| GO:0019637 | Organophosphate metabolic process | 42 | 0.35 | 0.00016 | - | - | - | - | - | - |
| GO:0072521 | Purine-containing compound metabolic process | 27 | 0.46 | 0.00016 | - | - | - | - | - | - |
| GO:0044087 | Regulation of cellular component biogenesis | 45 | 0.33 | 0.00017 | - | - | - | 12 | 0.75 | 0.0025 |
| GO:0010256 | Endomembrane system organization | 28 | 0.45 | 0.00018 | - | - | - | - | - | - |
| GO:0009259 | Ribonucleotide metabolic process | 26 | 0.47 | 2.00E-04 | - | - | - | - | - | - |
| GO:0009896 | Positive regulation of catabolic process | 28 | 0.44 | 2.00E-04 | - | - | - | - | - | - |
| GO:0032386 | Regulation of intracellular transport | 22 | 0.52 | 2.00E-04 | - | - | - | - | - | - |
| GO:0044089 | Positive regulation of cellular component biogenesis | 30 | 0.43 | 2.00E-04 | - | - | - | - | - | - |
| GO:0046390 | Ribose phosphate biosynthetic process | 16 | 0.63 | 0.00021 | - | - | - | - | - | - |
| GO:1902905 | Positive regulation of supramolecular fiber organization | 16 | 0.63 | 0.00022 | - | - | - | - | - | - |
| GO:0080134 | Regulation of response to stress | 51 | 0.3 | 0.00023 | - | - | - | - | - | - |
| GO:0030162 | Regulation of proteolysis | 35 | 0.38 | 0.00024 | - | - | - | - | - | - |
| GO:0051223 | Regulation of protein transport | 29 | 0.43 | 0.00024 | - | - | - | - | - | - |
| GO:0051495 | Positive regulation of cytoskeleton organization | 17 | 0.6 | 0.00024 | - | - | - | - | - | - |
| GO:0090407 | Organophosphate biosynthetic process | 27 | 0.45 | 0.00024 | - | - | - | - | - | - |
| GO:2000573 | Positive regulation of D- biosynthetic process | 12 | 0.76 | 0.00024 | - | - | - | - | - | - |
| GO:0034504 | Protein localization to nucleus | 17 | 0.6 | 0.00025 | - | - | - | - | - | - |
| GO:0002181 | Cytoplasmic translation | 15 | 0.64 | 3.00E-04 | - | - | - | - | - | - |
| GO:0032204 | Regulation of telomere mainte-nce | 12 | 0.75 | 3.00E-04 | - | - | - | - | - | - |
| GO:0008361 | Regulation of cell size | 18 | 0.57 | 0.00032 | - | - | - | - | - | - |
| GO:0051276 | Chromosome organization | 42 | 0.33 | 0.00032 | - | - | - | - | - | - |
| GO:1901700 | Response to oxygen-containing compound | 61 | 0.27 | 0.00034 | - | - | - | - | - | - |
| GO:0009966 | Regulation of sig-l transduction | 93 | 0.2 | 0.00036 | - | - | - | - | - | - |
| GO:0032535 | Regulation of cellular component size | 25 | 0.46 | 0.00036 | - | - | - | - | - | - |
| GO:0032212 | Positive regulation of telomere mainte-nce via telomerase | 8 | 0.98 | 0.00038 | - | - | - | - | - | - |
| GO:0000278 | Mitotic cell cycle | 32 | 0.39 | 4.00E-04 | - | - | - | - | - | - |
| GO:0006417 | Regulation of translation | 28 | 0.42 | 0.00049 | - | - | - | - | - | - |
| GO:2000278 | Regulation of D- biosynthetic process | 14 | 0.65 | 5.00E-04 | - | - | - | - | - | - |
| GO:0043254 | Regulation of protein-containing complex assembly | 24 | 0.46 | 0.00054 | - | - | - | 7 | 0.91 | 0.014 |
| GO:0032210 | Regulation of telomere mainte-nce via telomerase | 9 | 0.87 | 0.00055 | - | - | - | - | - | - |
| GO:1901701 | Cellular response to oxygen-containing compound | 45 | 0.31 | 0.00055 | - | - | - | - | - | - |
| GO:0044419 | Biological process involved in interspecies interaction between organisms | 52 | 0.28 | 0.00061 | - | - | - | - | - | - |
| GO:0050790 | Regulation of catalytic activity | 75 | 0.22 | 0.00061 | - | - | - | - | - | - |
| GO:0009150 | Purine ribonucleotide metabolic process | 24 | 0.45 | 0.00064 | - | - | - | - | - | - |
| GO:0080135 | Regulation of cellular response to stress | 32 | 0.38 | 0.00067 | - | - | - | - | - | - |
| GO:0034097 | Response to cytokine | 37 | 0.34 | 0.00072 | - | - | - | - | - | - |
| GO:0051222 | Positive regulation of protein transport | 20 | 0.5 | 0.00074 | - | - | - | - | - | - |
| GO:0031325 | Positive regulation of cellular metabolic process | 98 | 0.19 | 0.00076 | - | - | - | - | - | - |
| GO:0031399 | Regulation of protein modification process | 58 | 0.26 | 0.00079 | - | - | - | - | - | - |
| GO:0007339 | Binding of sperm to zo- pellucida | 7 | 1.01 | 0.00086 | - | - | - | - | - | - |
| GO:0032271 | Regulation of protein polymerization | 16 | 0.57 | 0.00086 | - | - | - | - | - | - |
| GO:2000112 | Regulation of cellular macromolecule biosynthetic process | 30 | 0.39 | 0.00086 | - | - | - | - | - | - |
| GO:0006998 | Nuclear envelope organization | 8 | 0.91 | 0.00093 | - | - | - | - | - | - |
| GO:0071495 | Cellular response to endogenous stimulus | 45 | 0.3 | 0.001 | - | - | - | - | - | - |
| GO:0044093 | Positive regulation of molecular function | 57 | 0.26 | 0.0011 | - | - | - | - | - | - |
| GO:0032446 | Protein modification by small protein conjugation | 32 | 0.36 | 0.0012 | - | - | - | - | - | - |
| GO:0048583 | Regulation of response to stimulus | 113 | 0.17 | 0.0012 | - | - | - | - | - | - |
| GO:0051338 | Regulation of transferase activity | 37 | 0.33 | 0.0013 | - | - | - | - | - | - |
| GO:0006997 | Nucleus organization | 13 | 0.63 | 0.0014 | - | - | - | - | - | - |
| GO:0016032 | Viral process | 13 | 0.63 | 0.0014 | - | - | - | - | - | - |
| GO:1901135 | Carbohydrate derivative metabolic process | 41 | 0.31 | 0.0014 | - | - | - | - | - | - |
| GO:0009988 | Cell-cell recognition | 8 | 0.88 | 0.0015 | - | - | - | - | - | - |
| GO:0031326 | Regulation of cellular biosynthetic process | 116 | 0.16 | 0.0015 | - | - | - | - | - | - |
| GO:2000058 | Regulation of ubiquitin-dependent protein catabolic process | 14 | 0.6 | 0.0015 | - | - | - | - | - | - |
| GO:0006793 | Phosphorus metabolic process | 66 | 0.23 | 0.0016 | - | - | - | - | - | - |
| GO:0009889 | Regulation of biosynthetic process | 118 | 0.16 | 0.0016 | - | - | - | - | - | - |
| GO:0023056 | Positive regulation of sig-ling | 61 | 0.24 | 0.0016 | - | - | - | - | - | - |
| GO:0006897 | Endocytosis | 23 | 0.43 | 0.0017 | - | - | - | 7 | 0.91 | 0.0144 |
| GO:0032434 | Regulation of proteasomal ubiquitin-dependent protein catabolic process | 12 | 0.66 | 0.0017 | - | - | - | - | - | - |
| GO:0051129 | Negative regulation of cellular component organization | 33 | 0.35 | 0.0017 | - | - | - | 10 | 0.82 | 0.0037 |
| GO:1901800 | Positive regulation of proteasomal protein catabolic process | 11 | 0.69 | 0.0017 | - | - | - | - | - | - |
| GO:0006796 | Phosphate-containing compound metabolic process | 65 | 0.23 | 0.002 | - | - | - | - | - | - |
| GO:0010556 | Regulation of macromolecule biosynthetic process | 111 | 0.16 | 0.002 | - | - | - | - | - | - |
| GO:0031116 | Positive regulation of microtubule polymerization | 7 | 0.94 | 0.002 | - | - | - | - | - | - |
| GO:1905666 | Regulation of protein localization to endosome | 5 | 1.22 | 0.002 | - | - | - | 3 | 1.99 | 0.0063 |
| GO:0007163 | Establishment or mainte-nce of cell polarity | 17 | 0.51 | 0.0021 | - | - | - | 6 | 1.05 | 0.0106 |
| GO:0022613 | Ribonucleoprotein complex biogenesis | 29 | 0.37 | 0.0021 | - | - | - | - | - | - |
| GO:0045785 | Positive regulation of cell adhesion | 24 | 0.41 | 0.0021 | - | - | - | - | - | - |
| GO:0051054 | Positive regulation of D- metabolic process | 20 | 0.46 | 0.0022 | - | - | - | - | - | - |
| GO:0006606 | Protein import into nucleus | 11 | 0.67 | 0.0024 | - | - | - | - | - | - |
| GO:0010647 | Positive regulation of cell communication | 60 | 0.23 | 0.0024 | - | - | - | - | - | - |
| GO:0032206 | Positive regulation of telomere mainte-nce | 9 | 0.77 | 0.0024 | - | - | - | - | - | - |
| GO:1900180 | Regulation of protein localization to nucleus | 13 | 0.6 | 0.0024 | - | - | - | - | - | - |
| GO:0071346 | Cellular response to interferon-gamma | 11 | 0.67 | 0.0025 | - | - | - | - | - | - |
| GO:1903320 | Regulation of protein modification by small protein conjugation or removal | 17 | 0.51 | 0.0025 | - | - | - | - | - | - |
| GO:0060284 | Regulation of cell development | 28 | 0.37 | 0.0028 | - | - | - | - | - | - |
| GO:0031324 | Negative regulation of cellular metabolic process | 72 | 0.2 | 0.0031 | - | - | - | - | - | - |
| GO:0031397 | Negative regulation of protein ubiquiti-tion | 10 | 0.7 | 0.0031 | - | - | - | - | - | - |
| GO:0051051 | Negative regulation of transport | 24 | 0.4 | 0.0031 | - | - | - | - | - | - |
| GO:0045653 | Negative regulation of megakaryocyte differentiation | 4 | 1.4 | 0.0033 | - | - | - | - | - | - |
| GO:2000643 | Positive regulation of early endosome to late endosome transport | 4 | 1.4 | 0.0033 | - | - | - | - | - | - |
| GO:0007010 | Cytoskeleton organization | 48 | 0.26 | 0.0034 | - | - | - | - | - | - |
| GO:0009895 | Negative regulation of catabolic process | 19 | 0.46 | 0.0034 | - | - | - | - | - | - |
| GO:0051984 | Positive regulation of chromosome segregation | 6 | 1 | 0.0034 | - | - | - | - | - | - |
| GO:0006694 | Steroid biosynthetic process | 10 | 0.69 | 0.0035 | - | - | - | - | - | - |
| GO:0030155 | Regulation of cell adhesion | 33 | 0.33 | 0.0036 | - | - | - | - | - | - |
| GO:1903047 | Mitotic cell cycle process | 26 | 0.38 | 0.0037 | - | - | - | - | - | - |
| GO:0009124 | Nucleoside monophosphate biosynthetic process | 7 | 0.88 | 0.0038 | - | - | - | - | - | - |
| GO:0006164 | Purine nucleotide biosynthetic process | 13 | 0.58 | 0.0039 | - | - | - | - | - | - |
| GO:0010941 | Regulation of cell death | 59 | 0.23 | 0.004 | - | - | - | - | - | - |
| GO:0043434 | Response to peptide hormone | 21 | 0.42 | 0.0041 | - | - | - | - | - | - |
| GO:0071345 | Cellular response to cytokine stimulus | 32 | 0.33 | 0.0041 | - | - | - | - | - | - |
| GO:0072665 | Protein localization to vacuole | 8 | 0.79 | 0.0041 | - | - | - | - | - | - |
| GO:0051172 | Negative regulation of nitrogen compound metabolic process | 74 | 0.2 | 0.0042 | - | - | - | - | - | - |
| GO:2000641 | Regulation of early endosome to late endosome transport | 5 | 1.12 | 0.0044 | - | - | - | - | - | - |
| GO:0009260 | Ribonucleotide biosynthetic process | 13 | 0.57 | 0.0046 | - | - | - | - | - | - |
| GO:0050793 | Regulation of developmental process | 79 | 0.19 | 0.0046 | - | - | - | - | - | - |
| GO:0006403 | R- localization | 13 | 0.57 | 0.0048 | - | - | - | - | - | - |
| GO:0042177 | Negative regulation of protein catabolic process | 10 | 0.67 | 0.0048 | - | - | - | - | - | - |
| GO:1904377 | Positive regulation of protein localization to cell periphery | 8 | 0.78 | 0.0049 | - | - | - | - | - | - |
| GO:0010628 | Positive regulation of gene expression | 45 | 0.26 | 0.0052 | - | - | - | - | - | - |
| GO:0016126 | Sterol biosynthetic process | 7 | 0.85 | 0.0052 | - | - | - | - | - | - |
| GO:0072666 | Establishment of protein localization to vacuole | 7 | 0.85 | 0.0052 | - | - | - | - | - | - |
| GO:0010468 | Regulation of gene expression | 129 | 0.13 | 0.0057 | - | - | - | - | - | - |
| GO:0002376 | Immune system process | 62 | 0.21 | 0.0058 | - | - | - | - | - | - |
| GO:0031396 | Regulation of protein ubiquiti-tion | 15 | 0.51 | 0.0058 | - | - | - | - | - | - |
| GO:0031497 | Chromatin assembly | 13 | 0.55 | 0.0061 | - | - | - | - | - | - |
| GO:1905477 | Positive regulation of protein localization to membrane | 10 | 0.66 | 0.0061 | - | - | - | - | - | - |
| GO:0006281 | D- repair | 23 | 0.39 | 0.0062 | - | - | - | - | - | - |
| GO:0006605 | Protein targeting | 15 | 0.5 | 0.0062 | - | - | - | - | - | - |
| GO:0045185 | Mainte-nce of protein location | 9 | 0.7 | 0.0062 | - | - | - | - | - | - |
| GO:2001242 | Regulation of intrinsic apoptotic sig-ling pathway | 12 | 0.58 | 0.0062 | - | - | - | - | - | - |
| GO:0050896 | Response to stimulus | 201 | 0.09 | 0.0064 | - | - | - | - | - | - |
| GO:0051983 | Regulation of chromosome segregation | 10 | 0.65 | 0.0064 | - | - | - | - | - | - |
| GO:0009725 | Response to hormone | 33 | 0.31 | 0.0069 | - | - | - | - | - | - |
| GO:0019220 | Regulation of phosphate metabolic process | 50 | 0.24 | 0.0069 | - | - | - | - | - | - |
| GO:0031330 | Negative regulation of cellular catabolic process | 15 | 0.5 | 0.0069 | - | - | - | - | - | - |
| GO:0051301 | Cell division | 21 | 0.4 | 0.0069 | - | - | - | - | - | - |
| GO:0070849 | Response to epidermal growth factor | 7 | 0.83 | 0.0069 | - | - | - | - | - | - |
| GO:2001233 | Regulation of apoptotic sig-ling pathway | 20 | 0.42 | 0.0069 | - | - | - | - | - | - |
| GO:0009156 | Ribonucleoside monophosphate biosynthetic process | 6 | 0.92 | 0.007 | - | - | - | - | - | - |
| GO:0051085 | Chaperone cofactor-dependent protein refolding | 6 | 0.92 | 0.007 | - | - | - | - | - | - |
| GO:0071108 | Protein K48-linked deubiquiti-tion | 6 | 0.92 | 0.007 | - | - | - | - | - | - |
| GO:0042325 | Regulation of phosphorylation | 45 | 0.25 | 0.0075 | - | - | - | - | - | - |
| GO:0045595 | Regulation of cell differentiation | 55 | 0.22 | 0.0075 | - | - | - | - | - | - |
| GO:0016050 | Vesicle organization | 16 | 0.47 | 0.0079 | - | - | - | - | - | - |
| GO:0090218 | Positive regulation of lipid ki-se activity | 6 | 0.91 | 0.0079 | - | - | - | - | - | - |
| GO:0051656 | Establishment of organelle localization | 20 | 0.41 | 0.008 | - | - | - | - | - | - |
| GO:0030838 | Positive regulation of actin filament polymerization | 7 | 0.81 | 0.0081 | - | - | - | - | - | - |
| GO:0042327 | Positive regulation of phosphorylation | 33 | 0.3 | 0.0081 | - | - | - | - | - | - |
| GO:0009408 | Response to heat | 11 | 0.6 | 0.0082 | - | - | - | - | - | - |
| GO:0051248 | Negative regulation of protein metabolic process | 39 | 0.27 | 0.0082 | - | - | - | - | - | - |
| GO:0060627 | Regulation of vesicle-mediated transport | 28 | 0.33 | 0.0082 | - | - | - | - | - | - |
| GO:0016567 | Protein ubiquiti-tion | 28 | 0.33 | 0.0087 | - | - | - | - | - | - |
| GO:0008037 | Cell recognition | 10 | 0.63 | 0.0088 | - | - | - | - | - | - |
| GO:0090066 | Regulation of a-tomical structure size | 26 | 0.35 | 0.0089 | - | - | - | - | - | - |
| GO:0060712 | Spongiotrophoblast layer development | 4 | 1.22 | 0.0091 | - | - | - | - | - | - |
| GO:0001558 | Regulation of cell growth | 21 | 0.39 | 0.0098 | - | - | - | - | - | - |
| GO:0019058 | Viral life cycle | 10 | 0.62 | 0.0099 | 6 | 1.01 | 0.0199 | - | - | - |
| GO:0030100 | Regulation of endocytosis | 15 | 0.48 | 0.01 | - | - | - | - | - | - |
| GO:0051664 | Nuclear pore localization | 3 | 1.57 | 0.01 | - | - | - | - | - | - |
| GO:2000026 | Regulation of multicellular organismal development | 50 | 0.23 | 0.0102 | - | - | - | - | - | - |
| GO:0007059 | Chromosome segregation | 17 | 0.44 | 0.0105 | - | - | - | - | - | - |
| GO:0019219 | Regulation of nucleobase-containing compound metabolic process | 107 | 0.14 | 0.0105 | - | - | - | - | - | - |
| GO:0045807 | Positive regulation of endocytosis | 10 | 0.61 | 0.011 | - | - | - | - | - | - |
| GO:0051347 | Positive regulation of transferase activity | 25 | 0.35 | 0.0112 | - | - | - | - | - | - |
| GO:1902533 | Positive regulation of intracellular sig-l transduction | 38 | 0.27 | 0.0113 | - | - | - | - | - | - |
| GO:0045834 | Positive regulation of lipid metabolic process | 12 | 0.54 | 0.0115 | - | - | - | - | - | - |
| GO:0006259 | D- metabolic process | 31 | 0.3 | 0.012 | - | - | - | - | - | - |
| GO:0006356 | Regulation of transcription by R- polymerase I | 6 | 0.86 | 0.012 | - | - | - | - | - | - |
| GO:0032507 | Mainte-nce of protein location in cell | 7 | 0.77 | 0.0123 | - | - | - | - | - | - |
| GO:0043067 | Regulation of programmed cell death | 52 | 0.22 | 0.0123 | - | - | - | - | - | - |
| GO:0016070 | R- metabolic process | 52 | 0.22 | 0.0124 | - | - | - | - | - | - |
| GO:1901652 | Response to peptide | 22 | 0.37 | 0.0132 | - | - | - | - | - | - |
| GO:1903078 | Positive regulation of protein localization to plasma membrane | 7 | 0.76 | 0.0133 | - | - | - | - | - | - |
| GO:0010720 | Positive regulation of cell development | 19 | 0.4 | 0.0135 | - | - | - | - | - | - |
| GO:0031099 | Regeneration | 12 | 0.53 | 0.0136 | - | - | - | - | - | - |
| GO:0031401 | Positive regulation of protein modification process | 38 | 0.26 | 0.0136 | - | - | - | - | - | - |
| GO:1905668 | Positive regulation of protein localization to endosome | 4 | 1.15 | 0.0139 | - | - | - | - | - | - |
| GO:1902115 | Regulation of organelle assembly | 14 | 0.48 | 0.0141 | - | - | - | - | - | - |
| GO:1902532 | Negative regulation of intracellular sig-l transduction | 24 | 0.35 | 0.0142 | - | - | - | - | - | - |
| GO:0042981 | Regulation of apoptotic process | 51 | 0.22 | 0.0144 | - | - | - | - | - | - |
| GO:0009607 | Response to biotic stimulus | 44 | 0.24 | 0.0145 | - | - | - | - | - | - |
| GO:0043403 | Skeletal muscle tissue regeneration | 6 | 0.84 | 0.0145 | - | - | - | - | - | - |
| GO:0048585 | Negative regulation of response to stimulus | 52 | 0.22 | 0.0145 | - | - | - | - | - | - |
| GO:0048589 | Developmental growth | 23 | 0.35 | 0.0145 | - | - | - | - | - | - |
| GO:1901699 | Cellular response to nitrogen compound | 28 | 0.31 | 0.0145 | - | - | - | - | - | - |
| GO:0007338 | Single fertilization | 10 | 0.59 | 0.0154 | - | - | - | - | - | - |
| GO:0031400 | Negative regulation of protein modification process | 24 | 0.34 | 0.0154 | - | - | - | - | - | - |
| GO:0050708 | Regulation of protein secretion | 16 | 0.44 | 0.0157 | - | - | - | - | - | - |
| GO:0010770 | Positive regulation of cell morphogenesis involved in differentiation | 9 | 0.63 | 0.0158 | - | - | - | - | - | - |
| GO:0042908 | Xenobiotic transport | 6 | 0.83 | 0.0158 | - | - | - | - | - | - |
| GO:0045814 | Negative regulation of gene expression, epigenetic | 8 | 0.68 | 0.0158 | - | - | - | - | - | - |
| GO:0046931 | Pore complex assembly | 5 | 0.95 | 0.0158 | - | - | - | - | - | - |
| GO:0009967 | Positive regulation of sig-l transduction | 51 | 0.22 | 0.0164 | - | - | - | - | - | - |
| GO:0031331 | Positive regulation of cellular catabolic process | 20 | 0.38 | 0.0174 | - | - | - | - | - | - |
| GO:0000910 | Cytokinesis | 9 | 0.62 | 0.0176 | - | - | - | - | - | - |
| GO:0050767 | Regulation of neurogenesis | 21 | 0.36 | 0.0177 | - | - | - | - | - | - |
| GO:0051668 | Localization within membrane | 24 | 0.34 | 0.0177 | - | - | - | - | - | - |
| GO:0051716 | Cellular response to stimulus | 165 | 0.1 | 0.0177 | - | - | - | - | - | - |
| GO:1901137 | Carbohydrate derivative biosynthetic process | 24 | 0.34 | 0.0177 | - | - | - | - | - | - |
| GO:0045787 | Positive regulation of cell cycle | 18 | 0.4 | 0.0193 | - | - | - | - | - | - |
| GO:0045087 | In-te immune response | 25 | 0.32 | 0.0197 | - | - | - | - | - | - |
| GO:1903706 | Regulation of hemopoiesis | 18 | 0.39 | 0.0204 | - | - | - | - | - | - |
| GO:0022411 | Cellular component disassembly | 16 | 0.42 | 0.0205 | - | - | - | - | - | - |
| GO:0051647 | Nucleus localization | 6 | 0.8 | 0.0207 | - | - | - | - | - | - |
| GO:0071364 | Cellular response to epidermal growth factor stimulus | 6 | 0.8 | 0.0207 | - | - | - | - | - | - |
| GO:0051321 | Meiotic cell cycle | 13 | 0.48 | 0.0211 | - | - | - | - | - | - |
| GO:0022414 | Reproductive process | 47 | 0.22 | 0.0212 | - | - | - | - | - | - |
| GO:0045638 | Negative regulation of myeloid cell differentiation | 8 | 0.66 | 0.0215 | - | - | - | - | - | - |
| GO:0009152 | Purine ribonucleotide biosynthetic process | 11 | 0.53 | 0.0217 | - | - | - | - | - | - |
| GO:0031507 | Heterochromatin assembly | 7 | 0.72 | 0.0217 | - | - | - | - | - | - |
| GO:0043085 | Positive regulation of catalytic activity | 40 | 0.24 | 0.0217 | - | - | - | - | - | - |
| GO:0072659 | Protein localization to plasma membrane | 13 | 0.48 | 0.0217 | - | - | - | - | - | - |
| GO:0032870 | Cellular response to hormone stimulus | 22 | 0.35 | 0.0221 | - | - | - | - | - | - |
| GO:0072528 | Pyrimidine-containing compound biosynthetic process | 5 | 0.9 | 0.0224 | - | - | - | - | - | - |
| GO:0046112 | Nucleobase biosynthetic process | 4 | 1.07 | 0.0231 | - | - | - | - | - | - |
| GO:0015849 | Organic acid transport | 15 | 0.43 | 0.0234 | - | - | - | - | - | - |
| GO:0006898 | Receptor-mediated endocytosis | 11 | 0.53 | 0.0235 | - | - | - | - | - | - |
| GO:0008203 | Cholesterol metabolic process | 9 | 0.6 | 0.0237 | - | - | - | - | - | - |
| GO:0060998 | Regulation of dendritic spine development | 9 | 0.6 | 0.0237 | - | - | - | - | - | - |
| GO:0030036 | Actin cytoskeleton organization | 24 | 0.32 | 0.0242 | - | - | - | - | - | - |
| GO:0070646 | Protein modification by small protein removal | 11 | 0.52 | 0.0244 | - | - | - | - | - | - |
| GO:0032069 | Regulation of nuclease activity | 5 | 0.89 | 0.0249 | - | - | - | - | - | - |
| GO:0045937 | Positive regulation of phosphate metabolic process | 34 | 0.26 | 0.0249 | - | - | - | - | - | - |
| GO:0061003 | Positive regulation of dendritic spine morphogenesis | 5 | 0.89 | 0.0249 | - | - | - | - | - | - |
| GO:0090169 | Regulation of spindle assembly | 5 | 0.89 | 0.0249 | - | - | - | - | - | - |
| GO:0009628 | Response to abiotic stimulus | 41 | 0.23 | 0.0252 | - | - | - | - | - | - |
| GO:0014070 | Response to organic cyclic compound | 35 | 0.26 | 0.0256 | - | - | - | - | - | - |
| GO:0016579 | Protein deubiquiti-tion | 10 | 0.55 | 0.0273 | - | - | - | - | - | - |
| GO:1901617 | Organic hydroxy compound biosynthetic process | 11 | 0.52 | 0.0273 | - | - | - | - | - | - |
| GO:0010592 | Positive regulation of lamellipodium assembly | 5 | 0.88 | 0.0274 | - | - | - | - | - | - |
| GO:0071417 | Cellular response to organonitrogen compound | 25 | 0.31 | 0.0277 | - | - | - | - | - | - |
| GO:1901799 | Negative regulation of proteasomal protein catabolic process | 6 | 0.77 | 0.0281 | - | - | - | - | - | - |
| GO:0040029 | Regulation of gene expression, epigenetic | 10 | 0.54 | 0.0285 | - | - | - | - | - | - |
| GO:0010811 | Positive regulation of cell-substrate adhesion | 10 | 0.54 | 0.0299 | - | - | - | - | - | - |
| GO:0051653 | Spindle localization | 7 | 0.68 | 0.0299 | - | - | - | - | - | - |
| GO:0099175 | Regulation of postsy-pse organization | 9 | 0.58 | 0.0299 | - | - | - | - | - | - |
| GO:0040008 | Regulation of growth | 27 | 0.29 | 0.0302 | - | - | - | - | - | - |
| GO:0007017 | Microtubule-based process | 33 | 0.26 | 0.0307 | - | - | - | - | - | - |
| GO:0043393 | Regulation of protein binding | 13 | 0.46 | 0.0307 | - | - | - | - | - | - |
| GO:0006974 | Cellular response to D- damage stimulus | 28 | 0.29 | 0.0308 | - | - | - | - | - | - |
| GO:0051701 | Biological process involved in interaction with host | 9 | 0.58 | 0.0312 | - | - | - | - | - | - |
| GO:0033047 | Regulation of mitotic sister chromatid segregation | 6 | 0.75 | 0.0329 | - | - | - | - | - | - |
| GO:0120035 | Regulation of plasma membrane bounded cell projection organization | 29 | 0.28 | 0.033 | - | - | - | - | - | - |
| GO:0006325 | Chromatin organization | 24 | 0.31 | 0.034 | - | - | - | - | - | - |
| GO:0051707 | Response to other organism | 41 | 0.23 | 0.0341 | - | - | - | - | - | - |
| GO:1905039 | Carboxylic acid transmembrane transport | 11 | 0.5 | 0.0342 | - | - | - | - | - | - |
| GO:0019856 | Pyrimidine nucleobase biosynthetic process | 3 | 1.27 | 0.0343 | - | - | - | - | - | - |
| GO:0035967 | Cellular response to topologically incorrect protein | 8 | 0.61 | 0.0346 | - | - | - | - | - | - |
| GO:0007346 | Regulation of mitotic cell cycle | 21 | 0.33 | 0.0348 | - | - | - | - | - | - |
| GO:0051235 | Mainte-nce of location | 11 | 0.5 | 0.0352 | - | - | - | - | - | - |
| GO:0140694 | Non-membrane-bounded organelle assembly | 19 | 0.35 | 0.0359 | - | - | - | - | - | - |
| GO:0032435 | Negative regulation of proteasomal ubiquitin-dependent protein catabolic process | 5 | 0.84 | 0.0366 | - | - | - | - | - | - |
| GO:0044403 | Biological process involved in symbiotic interaction | 12 | 0.47 | 0.0366 | - | - | - | - | - | - |
| GO:0089718 | Amino acid import across plasma membrane | 5 | 0.84 | 0.0366 | - | - | - | - | - | - |
| GO:0006914 | Autophagy | 15 | 0.41 | 0.0368 | - | - | - | - | - | - |
| GO:0035966 | Response to topologically incorrect protein | 9 | 0.56 | 0.0371 | - | - | - | - | - | - |
| GO:0009605 | Response to exter-l stimulus | 68 | 0.16 | 0.0374 | - | - | - | - | - | - |
| GO:0050714 | Positive regulation of protein secretion | 10 | 0.52 | 0.0374 | - | - | - | - | - | - |
| GO:0016570 | Histone modification | 20 | 0.34 | 0.0377 | - | - | - | - | - | - |
| GO:0043549 | Regulation of ki-se activity | 28 | 0.28 | 0.0388 | - | - | - | - | - | - |
| GO:0045637 | Regulation of myeloid cell differentiation | 12 | 0.46 | 0.0388 | - | - | - | - | - | - |
| GO:0009168 | Purine ribonucleoside monophosphate biosynthetic process | 4 | 0.98 | 0.0397 | - | - | - | - | - | - |
| GO:0045216 | Cell-cell junction organization | 12 | 0.46 | 0.0401 | - | - | - | - | - | - |
| GO:0050769 | Positive regulation of neurogenesis | 15 | 0.4 | 0.0406 | - | - | - | - | - | - |
| GO:0051960 | Regulation of nervous system development | 23 | 0.31 | 0.0406 | - | - | - | - | - | - |
| GO:0042254 | Ribosome biogenesis | 20 | 0.34 | 0.0418 | - | - | - | - | - | - |
| GO:0032077 | Positive regulation of deoxyribonuclease activity | 3 | 1.22 | 0.0422 | - | - | - | - | - | - |
| GO:0010810 | Regulation of cell-substrate adhesion | 13 | 0.44 | 0.0423 | - | - | - | - | - | - |
| GO:0010506 | Regulation of autophagy | 15 | 0.4 | 0.0427 | - | - | - | - | - | - |
| GO:0051098 | Regulation of binding | 18 | 0.36 | 0.0428 | - | - | - | - | - | - |
| GO:2000113 | Negative regulation of cellular macromolecule biosynthetic process | 11 | 0.48 | 0.0428 | - | - | - | - | - | - |
| GO:0006623 | Protein targeting to vacuole | 5 | 0.82 | 0.0429 | - | - | - | - | - | - |
| GO:0030010 | Establishment of cell polarity | 10 | 0.51 | 0.0429 | - | - | - | - | - | - |
| GO:0048584 | Positive regulation of response to stimulus | 63 | 0.17 | 0.0429 | - | - | - | - | - | - |
| GO:0051239 | Regulation of multicellular organismal process | 80 | 0.15 | 0.0429 | - | - | - | - | - | - |
| GO:0072595 | Mainte-nce of protein localization in organelle | 5 | 0.82 | 0.0429 | - | - | - | - | - | - |
| GO:0098813 | Nuclear chromosome segregation | 13 | 0.43 | 0.0429 | - | - | - | - | - | - |
| GO:1902229 | Regulation of intrinsic apoptotic sig-ling pathway in response to D- damage | 5 | 0.82 | 0.0429 | - | - | - | - | - | - |
| GO:0007589 | Body fluid secretion | 7 | 0.64 | 0.0435 | - | - | - | - | - | - |
| GO:0006206 | Pyrimidine nucleobase metabolic process | 4 | 0.96 | 0.0441 | - | - | - | - | - | - |
| GO:0051156 | Glucose 6-phosphate metabolic process | 4 | 0.96 | 0.0441 | - | - | - | - | - | - |
| GO:0071216 | Cellular response to biotic stimulus | 12 | 0.45 | 0.0445 | - | - | - | - | - | - |
| GO:0002064 | Epithelial cell development | 12 | 0.45 | 0.0461 | - | - | - | 5 | 1.06 | 0.0345 |
| GO:0010975 | Regulation of neuron projection development | 23 | 0.3 | 0.0461 | - | - | - | - | - | - |
| GO:0051236 | Establishment of R- localization | 10 | 0.51 | 0.0461 | - | - | - | - | - | - |
| GO:0032873 | Negative regulation of stress-activated MAPK cascade | 5 | 0.8 | 0.0462 | - | - | - | - | - | - |
| GO:0061640 | Cytoskeleton-dependent cytokinesis | 8 | 0.58 | 0.0467 | - | - | - | - | - | - |
| GO:0070848 | Response to growth factor | 21 | 0.32 | 0.0478 | - | - | - | - | - | - |
| GO:0009314 | Response to radiation | 19 | 0.34 | 0.0487 | - | - | - | - | - | - |
| GO:0015931 | Nucleobase-containing compound transport | 12 | 0.45 | 0.0487 | - | - | - | - | - | - |
| GO:0071214 | Cellular response to abiotic stimulus | 15 | 0.39 | 0.0487 | - | - | - | - | - | - |
| GO:0090068 | Positive regulation of cell cycle process | 14 | 0.41 | 0.0487 | - | - | - | - | - | - |
| GO:0007034 | Vacuolar transport | 10 | 0.5 | 0.049 | - | - | - | - | - | - |
| GO:0046887 | Positive regulation of hormone secretion | 10 | 0.5 | 0.049 | - | - | - | - | - | - |
| GO:0006015 | 5-phosphoribose 1-diphosphate biosynthetic process | 3 | 1.17 | 0.0497 | - | - | - | - | - | - |
| GO:0006695 | Cholesterol biosynthetic process | 5 | 0.79 | 0.0497 | - | - | - | - | - | - |
| GO:0007032 | Endosome organization | 6 | 0.7 | 0.0497 | - | - | - | - | - | - |
| GO:0007097 | Nuclear migration | 5 | 0.79 | 0.0497 | - | - | - | - | - | - |
| GO:0032570 | Response to progesterone | 5 | 0.79 | 0.0497 | - | - | - | - | - | - |
| GO:0043534 | Blood vessel endothelial cell migration | 5 | 0.79 | 0.0497 | - | - | - | - | - | - |
| GO:0045116 | Protein neddylation | 3 | 1.17 | 0.0497 | - | - | - | - | - | - |
| GO:0060996 | Dendritic spine development | 5 | 0.79 | 0.0497 | - | - | - | - | - | - |
| GO:0090235 | Regulation of metaphase plate congression | 3 | 1.17 | 0.0497 | - | - | - | - | - | - |
| GO:1903051 | Negative regulation of proteolysis involved in protein catabolic process | 7 | 0.63 | 0.0497 | - | - | - | - | - | - |
| GO:0031623 | Receptor inter-lization | - | - | - | - | - | - | 4 | 1.46 | 0.0096 |
